# Extinction-recruited prefrontal-hypothalamic pathway encodes positive affect to sustain fear extinction in posttraumatic stress mouse model

**DOI:** 10.1172/JCI206270

**Published:** 2026-08-04

**Authors:** Ze-Jie Lin, Xin-Rong Wu, Ming-Yang Wei, Zheng-Kai Lao, Xiang Lan, Yan-Jiao Wu, Wei-Guang Li, Tian-Le Xu, Li-Na Huang, Xue Gu

**Affiliations:** 1 Department of Anesthesiology, Shanghai General Hospital;; 2 Department of Anatomy and Physiology, Shanghai Key Laboratory of Emotions and Affective Disorders (LEAD); and; 3 Department of Neurology and Institute of Neurology, Ruijin Hospital, Shanghai Jiao Tong University School of Medicine, Shanghai, China.; 4 Department of Rehabilitation Medicine, Huashan Hospital, Center for Clinical Neuro-AI, Institute for Translational Brain Research, State Key Laboratory of Brain Function and Disorders, Ministry of Education Frontiers Center for Brain Science, Fudan University, Shanghai, China.; 5 Department of Anesthesiology, Songjiang Research Institute, Songjiang Hospital Affiliated to Shanghai Jiao Tong University School of Medicine, Shanghai, China.

**Keywords:** Cell biology, Neuroscience, Memory, Mouse models, Psychiatric diseases

## Abstract

Effective psychotherapeutic interventions for posttraumatic stress disorder (PTSD) rely on fear extinction to suppress maladaptive fear responses, yet their long-term efficacy is limited by high relapse rates. Notably, extinction involves not only fear inhibition but also affective engagement. However, whether and how internal affective components contribute to extinction retrieval and long-term persistence remain unclear. Here, we demonstrate that positive affective experiences arising during extinction govern the long-term persistence of extinction and resistance to spontaneous recovery. We identify a subpopulation of medial prefrontal cortex (mPFC) extinction neurons projecting to supramammillary nucleus glutamatergic (SuM^Glu^) neurons that encode positive affective experience during extinction and selectively govern long-term extinction persistence. This ensemble is spatially, anatomically, and transcriptionally distinct from mPFC extinction neurons projecting to zona incerta somatostatin-expressing (ZI^SST^) neurons, which primarily support extinction retrieval. Transcriptomic profiling reveals enrichment of sirtuin 1 (*Sirt1*) within SuM-projecting extinction ensembles, and bidirectional manipulation of SIRT1 alters extinction relapse vulnerability in a PTSD mouse model. These findings provide a cortical-hypothalamic framework incorporating molecular features that governs the long-term persistence of fear extinction and resistance to relapse through positive affective processes.

## Abstract 2

A prefrontal-hypothalamic circuit links positive affect to the long-term persistence of fear extinction, helping prevent PTSD relapse.

## Graphical abstract

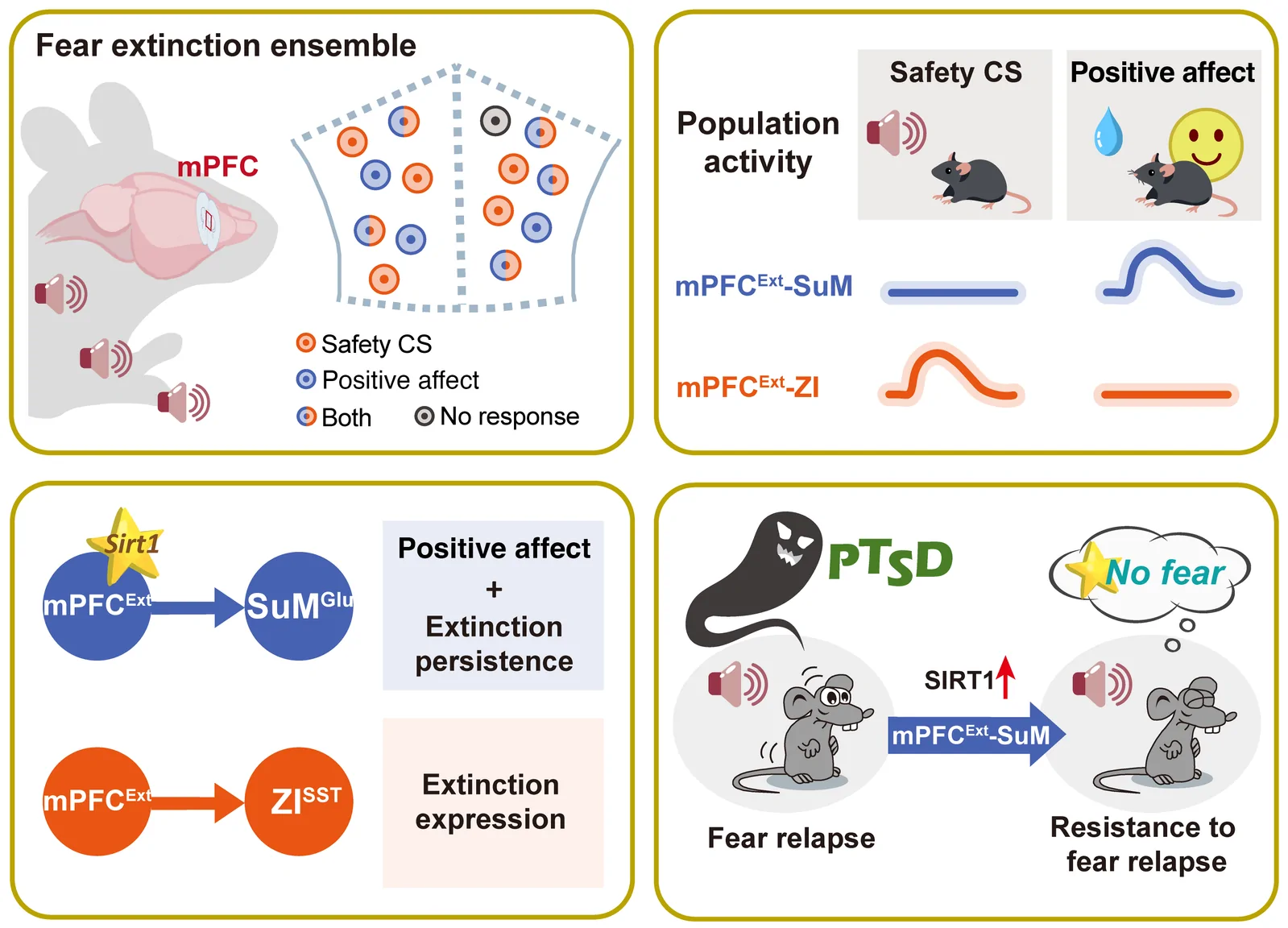

## Introduction

Fear extinction enables the adaptive mitigation of traumatic memories and is fundamental to exposure-based psychotherapy for anxiety disorders and posttraumatic stress disorder (PTSD) (1, 2). Although extinction learning can effectively reduce fear expression in the short term, its therapeutic efficacy is often limited by relapse, as extinguished fear responses can reemerge over time (3, 4). This vulnerability indicates that initial extinction expression and fear suppression alone are insufficient to ensure the persistence of extinction memory. Emotional dysregulation is closely associated with relapse in stress-related disorders (5–7), suggesting a potential role for affective processes in extinction persistence. This raises the question of whether and how internal affective processes govern extinction retrieval and persistence.

Fear extinction is increasingly recognized as the process of inhibitory learning to suppress the original fear memory, while simultaneously incorporating sensory, contextual, and affective components through the acquisition of safety information and behavioral flexibility (8, 9). Previous studies have shown that extinction engages circuits associated with positive valence and reward (10, 11). These findings suggest that successful extinction may require the recruitment of neural ensembles that are not only capable of suppressing fear but also responsive to positive emotional experiences, raising the possibility that positive affective processes contribute to extinction memory.

The medial prefrontal cortex (mPFC) is a key locus in the neural circuitry underlying fear and extinction, which governs various aspects of affective processing, perception of threatening state, and adaptive behaviors (12, 13). Importantly, the mPFC contains heterogeneous neuronal populations that project to diverse downstream targets involved in motivation, reward, emotion, and cognition, thereby exerting top-down control of behaviors (14–17). In addition, these projection-defined neurons receive long-range inputs from the thalamus, amygdala, and hippocampus, enabling hierarchical regulation of complex brain function (18, 19). Dissecting projection-defined prefrontal ensembles is therefore critical for understanding how affective signals shape functional architecture and long-term persistence of extinction memory. Extinction-related neurons have been identified within the mPFC, and reactivation of these specific populations has been suggested as a mechanistic correlate of extinction memory expression (20, 21). However, it remains unclear whether these ensembles exhibit functional heterogeneity in terms of emotional experiences.

In this study, we uncover the functional architecture of extinction ensembles within the mPFC and identify an affect-associated projection-defined ensemble that governs extinction persistence and resistance to relapse. Rather than viewing extinction ensembles as a uniform population, our findings reveal heterogeneity within mPFC extinction–related neurons, with distinct subpopulations differing in their responsiveness to positive affect. A SuM-projecting subset encoding positive affect during extinction functions not to suppress fear directly but to promote extinction persistence and resistance to relapse, whereas a ZI-projecting subset primarily supports extinction retrieval. Transcriptomic profiling identifies SIRT1 as a molecular determinant selectively enriched within SuM-projecting extinction ensembles. Bidirectional manipulation of SIRT1 alters relapse vulnerability in a PTSD mouse model. We demonstrate a cortical-hypothalamic framework in which positive affect–related prefrontal ensembles, endowed with specific molecular characteristics, selectively promote extinction persistence and confer resistance to relapse in trauma-related disorders.

## Results

### Positive affective experience selectively reengages a subset of extinction-tagged ensemble in the mPFC.

To identify neuronal populations recruited during fear extinction, we employed an auditory cued fear extinction paradigm. On day 1, mice received 5 foot shocks paired with a neutral auditory cue (conditioned stimulus, CS) and exhibited characteristic freezing behavior as an indicator of associative fear memory for the CS. On days 2–4, mice underwent extinction training consisting of repeated CS presentations without foot shock (12 CS trials per day), leading to a progressive reduction of freezing behavior (Supplemental Figure 1, A and B; supplemental material available online with this article; https://doi.org/10.1172/JCI206270DS1). We used the targeted recombination in active populations (TRAP) strategy in FosTRAP2:H2B-GFP mice (22, 23) and administered 4-hydroxytamoxifen (4-OHT) 30 minutes before day 4, inducing persistent H2B-GFP expression in extinction-activated neurons (Ext-tagged ensemble) (Supplemental Figure 1, C–E). The different parts of the mPFC, namely prelimbic (PL) and infralimbic (IL) cortex, have been separated and are thought to play distinct roles in the process of fear and extinction (24, 25). However, TRAP labelling indicated the presence of extinction ensembles in both PL and IL regions (Supplemental Figure 1F). Extinguished fear can be readily relapsed when individuals are presented with CS in conditioning context, a process termed as fear renewal (26). Behaviorally, the fear renewal test evoked a much higher fear response than the extinction retrieval test (Supplemental Figure 1B). Compared with fear renewal, Ext-tagged neurons were robustly reactivated during extinction retrieval (Supplemental Figure 1, G–I).

To further validate the functional relevance of extinction-tagged neurons, we performed optogenetic manipulation of mPFC extinction (mPFC^Ext^) ensemble. We injected a Cre-dependent AAV coding for halorhodopsin (NpHR) or channelrhodopsin-2 (ChR2) into the mPFC of FosTRAP2 mice (Figure 1, A and B). Inhibition of mPFC^Ext^ ensemble impaired retrieval performance, whereas activation facilitated extinction retrieval (Figure 1, C and F). In addition to their role in safety-related memory retrieval, manipulation of mPFC^Ext^ ensemble also bidirectionally influenced affect-related behaviors. Optogenetic inhibition of the mPFC^Ext^ ensemble abolished animals’ natural preference for sucrose water (Figure 1, D and E). Activation of the extinction ensemble induced a robust real-time place preference (RTPP) (Figure 1G), without affecting exploratory behaviors or basal anxiety (Supplemental Figure 2). These results confirm that extinction-tagged neurons participate in both extinction retrieval and positive affective behaviors at the population level.

We next asked whether extinction-tagged neurons are uniformly engaged across behavioral states or whether they contain subpopulations differentially responsive to positive affective experience. To address this question, we examined the activity of Ext-tagged ensemble during a positive affective condition. Water-deprived mice were allowed to consume water, a manipulation known to reliably evoke a positive affective state. To further characterize the activity at the single-cell level, we performed in vivo optrode recordings in the mPFC. A Cre-dependent AAV encoding ChR2 was injected into the mPFC of FosTRAP2 mice. Subsequently, we monitored the activity of ChR2-tagged extinction neurons during extinction retrieval and a positive experiential state (water intake) (Figure 2, A and B). Forty-three neurons were classified as ChR2-tagged neurons with short latencies of light-evoked spikes (Figure 2, C and D). These tagged neurons showed heterogeneous firing patterns under different behavioral conditions, with some preferentially associated with extinction retrieval, and others selectively engaged by positive affective experience (Figure 2, E and F). During extinction retrieval, a subset of extinction-tagged neurons showed a robust increase in firing activity (72.09%). Among the remaining non-responsive tagged neurons (27.91%), a considerable number responded to water intake. Meanwhile, responses to water intake varied markedly across the same population. Some neurons increased firing during water intake (55.81%), while others remained unchanged (44.19%). Among neurons nonresponsive to water intake, the majority responded to extinction retrieval (Figure 2G). These results indicate substantial heterogeneity in affective responsiveness among extinction-tagged neurons and suggest that extinction retrieval and positive affective experience engage partially overlapping but distinct subpopulations within the mPFC^Ext^ ensemble. Notably, we identified a subset of extinction-tagged neurons that was activated during positive experience while showing little or no response during extinction retrieval. Together, these observations reveal activity heterogeneity within mPFC^Ext^ ensemble, including a subset selectively engaged by positive experience but decoupled from extinction retrieval.

### Differential engagement of projection-specific mPFC^Ext^ ensemble during extinction retrieval and positive affective experience.

The activity heterogeneity observed within the extinction-tagged ensemble raises the possibility that distinct subpopulations may be differentially engaged by extinction retrieval and positive affective experience. We therefore asked whether this reflects the presence of a unique subpopulation, which is preferentially engaged by positive affective experience. To address this question, we first traced the axonal projections of mPFC^Ext^ ensemble using anterograde tracing. In addition, we confirmed that mPFC^Ext^ neurons are glutamatergic (Figure 3A). The neurons we mainly tagged are the pyramidal neurons in mPFC layer 5, which are the core outputs of the mPFC. These pyramidal neurons play a crucial role in cognitive, emotional, and behavioral regulation through specific projections and synaptic connections (27). Many downstream targets of mPFC^Ext^ ensemble were observed in the hypothalamus (medial preoptic area [MPO], lateral hypothalamic area [LHA], zona incerta [ZI], supramammillary nucleus [SuM]), thalamus (anteromedial nucleus [AM], mediodorsal nucleus of thalamus [MD]), and periaqueductal grey (PAG) (Figure 3B). Most downstream regions were involved in various aspects of memory or emotional processing or conditioned reward (28–31).

To functionally assess projection-specific dynamics, we injected AAV-CreOn-FlpOn-GCaMP6s into the mPFC and Retro-Flp into different downstream regions of FosTRAP2 mice (Supplemental Figure 3A). During extinction retrieval, the most prominently increased calcium signals occurred in ZI-projecting neurons, whereas these signals were suppressed during fear renewal (Supplemental Figure 3, B and C). While the majority of projectors were robustly activated during extinction retrieval, we unexpectedly found a distinct subpopulation (SuM-projecting mPFC^Ext^ ensemble) that exhibited minimal responses to the extinction retrieval. Strikingly, this subset was selectively activated during water consumption (Supplemental Figure 3, B–E). In addition, the basolateral amygdala (BLA), a key downstream target of the mPFC (Supplemental Figure 4A), plays an important and complex role in regulating fear and extinction learning (32, 33). Retrograde tracing revealed that BLA-projecting neurons are layer-specific in the mPFC, mainly distributed in layers 2 and 3 (Supplemental Figure 4B). Fiber photometry showed that the activity of BLA-projecting mPFC^Ext^ neurons increased during extinction retrieval but did not respond to water intake (Supplemental Figure 4, C–F). Then, we injected Retro-DIO-mCherry into the ZI or SuM of FosTRAP2 mice. Following extinction retrieval or water intake, c-Fos staining showed increased reactivation in ZI-projecting mPFC^Ext^ neurons during extinction retrieval, while SuM-projecting mPFC^Ext^ neurons were preferentially activated by water intake (Supplemental Figure 5, A–C). These results identify a projection-specific subpopulation of mPFC^Ext^ ensembles that encodes positive emotional experience but does not directly participate in extinction retrieval.

### SuM-projecting mPFC extinction ensembles are selectively engaged by positive affective experience.

We next performed single-unit electrophysiological recordings from opto-tagged neurons by injecting Retro-DIO-ChR2 into ZI or SuM of the FosTRAP2 mice, with optrodes implanted in mPFC (Figure 3, C–E). Short-latency light responses identified 37 ZI- and 32 SuM-projecting neurons (Supplemental Figure 5, D, E, H, and I). As shown by an example ChR2-tagged ZI-projecting mPFC extinction neuron and the population response (Supplemental Figure 5F), the majority of ZI-projecting mPFC^Ext^ ensembles (56.76%, 21 of 37 units) were excited by extinction retrieval (Supplemental Figure 5G). By contrast, a larger fraction (71.87%, 23 of 32 units) of SuM-projecting mPFC^Ext^ ensembles was excited by water intake, whereas a small fraction of the population (21.88%, 7 of 32 units) was excited by extinction retrieval (Supplemental Figure 5, J and K). Hierarchical clustering based on neuronal activities grouped neurons into 2 principal clusters that preferentially responded to extinction retrieval or positive emotional experience (Figure 3, F and G).

To enable visualization of the entire ensemble dynamics across time in trial-averaged neural trajectories, we examined the trajectory of the neural state space of ZI- and SuM-projecting mPFC^Ext^ ensembles during extinction retrieval and positive affective experience by using principal component analysis (PCA) (34, 35). Separating the neural trajectories in the activity space revealed that trajectories in the ZI-projecting mPFC^Ext^ ensembles showed greater trajectory distance during extinction retrieval, while trajectories in the SuM-projecting mPFC^Ext^ ensembles showed significant increases in trajectory distance during water intake (Figure 3, H and I). Furthermore, although population activity in both ZI- and SuM-projecting mPFC^Ext^ ensembles enabled decoding of extinction retrieval and water intake above shuffled controls (Figure 3, J and K), we observed a projection-specific bias in decoding performance. Decoding based on population activity from ZI-projecting ensembles yielded higher accuracy for extinction retrieval, whereas decoding based on population activity from SuM-projecting ensembles yielded higher accuracy for water intake. These findings demonstrate that the activity of ZI- and SuM-projecting mPFC^Ext^ ensembles contains sufficient information to preferentially represent extinction retrieval and positive emotional experience, respectively.

In addition, we found that mice showed pronounced individual variability in water intake after fear conditioning, extinction, and water deprivation. Unsupervised *K*-means clustering of consumption behavior classified mice as low-intake or normal-intake (Figure 3, L and N, and Supplemental Figure 6A). The low-intake mice exhibited markedly reduced water consumption, reflecting reduced behavioral responsiveness to positive affective experience, rather than physiological differences (Supplemental Figure 6, B and C). Notably, among the opto-tagged SuM-projecting subpopulations, the majority of neurons recorded from low-intake mice belonged to Cluster III, which was not responsive to affective value (Figure 3O). Instead, this phenomenon was not observed in ZI-projecting subpopulations. The neurons recorded were randomly distributed across the 3 clusters (Figure 3M). These observations provide converging evidence that a subset of SuM-projecting extinction neurons is selectively associated with the capacity to engage positive affective experience.

### SuM- and ZI-projecting mPFC extinction ensembles represent segregated populations with distinct morphological properties and topographical organizations.

We next examined whether SuM-projecting extinction ensembles represent a neuronal population fundamentally distinct from ZI-projecting extinction ensembles. To holistically compare the anatomical positioning of distinct mPFC^Ext^ ensembles, we performed dual retrograde tracing by injecting Retro-DIO-mCherry and Retro-DIO-EGFP into the ZI and SuM, respectively (Figure 4A). Retrograde tracing showed that mPFC^Ext^ ensembles projecting to the 2 regions concentrated in layer 5, but with minimal colocalization (Figure 4, B and C). Furthermore, the mPFC^Ext^–ZI and mPFC^Ext^–SuM projectors are distributed across different subregions. Most mPFC^Ext^–ZI projectors were distributed in the PL region and cingulate area 1 (Cg1), while most mPFC^Ext^–SuM projectors were distributed in the IL region (Supplemental Figure 7A) (27). To separately label collateral targets of ZI- or SuM-projecting mPFC^Ext^ ensembles, we injected Retro-Flp into the ZI or SuM and AAV-CreOn-FlpOn-EYFP into the mPFC of FosTRAP2 mice. The results showed that mPFC^Ext^–SuM projectors were more inclined to the pallidum region, which was related to motivational behaviors (36), but had negligible collaterals in the ZI (Supplemental Figure 7, B–D). Then, we reconstructed their morphological properties using sparse labeling (Figure 4, D and E). There was no significant difference in dendritic complexity between the 2 groups, while the ZI-projecting mPFC^Ext^ ensemble showed longer apical tuft and larger soma volume (Figure 4, F–H). Taken together, these data suggest that SuM-projecting mPFC^Ext^ ensembles representing positive affective component are anatomically and morphologically distinct from another cluster of neurons.

To better understand synaptic inputs of the different subpopulations, we used projection-specific tracing to map upstream inputs to the ZI- and SuM-innervating mPFC^Ext^ ensembles. We first injected AAV-DIO-RVG and AAV-DIO-H2B-EGFP-TVA into the mPFC of FosTRAP2 mice. After a week, mice were trained and then administered for TRAP labeling. Two weeks later, RV-ENVA-ΔG-DsRed was injected into the ZI or SuM (Figure 4I). Both populations received extensive inputs from cortical regions, thalamus, and hippocampus. However, ZI-projecting mPFC^Ext^ populations received proportionally more inputs from orbitofrontal cortex (ORB), piriform cortex (Pir), and BLA, whereas SuM-projecting mPFC^Ext^ populations received more inputs from central medial nucleus of the thalamus (CM) (Figure 4, J–L). This distinguishable projection-specific connectivity may underlie the ability of the mPFC^Ext^ ensembles to segregate memory retrieval and positive affective component. Overall, these findings demonstrate that the SuM-projecting mPFC^Ext^ neurons encoding positive emotional experiences constitute an anatomically, projectionally, and morphologically distinct neuronal population within the mPFC extinction ensemble.

### mPFC^Ext^ projections to SuM^Glu^ selectively modulate positive affective experience and extinction persistence without engaging extinction expression.

The mPFC^Ext^ ensembles comprise anatomically heterogeneous projectors characterized by a diverse organization of architecture that selectively routes information to downstream brain regions. To dissect the downstream cell type–specific connectivity of mPFC^Ext^ ensembles, the Cre dependent helper virus and anterograde herpes simplex virus–tdTomato (HSV-tdTomato) were injected into the mPFC (Figure 5A). Given that the diversity of GABAergic subpopulations contributes to the functional heterogeneity of the ZI (37, 38), we performed RNAscope and found that parvalbumin-expressing (PV-expressing) and somatostatin-expressing (SST-expressing) neurons constitute the major GABAergic subpopulations within the ZI (Supplemental Figure 8), consistent with previous study (39). Further analysis revealed that SST neurons were the predominant postsynaptic targets of mPFC^Ext^ projections in the ZI (Figure 5B). To directly assess the effect of mPFC^Ext^ projectors on ZI^SST^ neurons, we introduced AAV-DIO-ChrimsonR-mCherry into the mPFC and Flp-dependent AAV-fDIO-GCaMP6m into the ZI of FosTRAP2:SST-Flp mice (Figure 5C). Activation of mPFC^Ext^ terminals via red light illumination in the ZI substantially increased calcium signals in ZI^SST^ neurons (Figure 5D). Meanwhile, we observed that activation of mPFC^Ext^ terminals did not elicit increased calcium signals in ZI PV neurons (Supplemental Figure 9, A–C). Taken together, these results establish that ZI-projecting extinction ensembles primarily innervate SST neurons in the ZI.

The activity heterogeneity of the mPFC^Ext^ neurons led us to examine whether manipulation of specific pathways affects behavioral outputs in a feature-dependent way. Optogenetic inhibition of mPFC^Ext^-ZI projection suppressed extinction retrieval but had no effect on sucrose preference (Figure 5, E–H). These results suggest that the mPFC^Ext^-ZI pathway is essential for reduced fear expression induced by safety-associated CS signals.

Notably, extinction does not permanently eliminate the original fear memory. Instead, extinguished fear can return or relapse spontaneously over time, a phenomenon known as spontaneous recovery and characterized by robust freezing in the extinction-specific context (40, 41). We next assessed the effects of projection-specific manipulations during both extinction retrieval and spontaneous recovery test (SR test). We injected Retro-DIO-ChR2-mCherry (or Retro-DIO-mCherry as the control) and AAV-fDIO-hM4Di-EGFP viruses into the ZI of FosTRAP2:SST-Flp mice and simultaneously implanted optical fibers into the mPFC (Figure 5I). Optogenetic stimulation of mPFC^Ext^–ZI pathway facilitated the expression of extinction memory during extinction retrieval and SR test without eliciting positive affect–related place preference, and this effect was reversed when ZI^SST^ neurons were inhibited (Figure 5, J–L, and Supplemental Figure 10, A and B). These results identify the mPFC^Ext^–ZI^SST^ pathway as a dedicated circuit for extinction expression without engaging positive affective processes.

Similar to the above strategy, HSV-based anterograde tracing and fiber photometry revealed that SuM-projecting mPFC^Ext^ ensembles preferentially innervate glutamatergic (Glu) neurons rather than dopaminergic neurons in the SuM (Figure 6, A–D, and Supplemental Figure 9 D–F). The specificity of targeting glutamatergic neurons was confirmed by RNAscope staining for *Slc17a6^+^* neurons (expressing vesicular glutamate transporter 2, VGLUT2) (Supplemental Figure 10, E and F). We further investigated whether manipulation of mPFC^Ext^–SuM^Glu^ pathway modulates positive affect–related behavior and fear extinction memory. Optogenetic inhibition of mPFC^Ext^–SuM projection disrupted the preference for sucrose without altering extinction retrieval (Figure 6, E–H). Optogenetic activation of mPFC^Ext^–SuM projection also had no effect on extinction retrieval and could facilitate positive affect–related behavior. Intriguingly, stimulating mPFC^Ext^–SuM projection, which was not involved in memory retrieval, significantly reduced fear response during spontaneous recovery. And this reduction in fear response during the spontaneous recovery test was reversed when SuM^Glu^ neurons were inhibited (Figure 6, I–L, Supplemental Figure 10, C and D, and Supplemental Figure 11).

The behavioral effects mediated by SuM-projecting mPFC^Ext^ neurons are completely different between extinction retrieval and long-term retrieval, the latter corresponding to spontaneous recovery. However, we observed no difference in the number of extinction-tagged neurons between these two periods (Supplemental Figure 12, A–C). To rule out delayed differences of ChR2 expression, we performed control experiments using the dual-control system (42) of robust activity marking (RAM) and AAVs expressing doxycycline-dependent (Dox-dependent) CreERT2 and Cre-dependent retrograde ChR2 or mCherry microinjected in mPFC and SuM. The mice were maintained on Dox throughout the experiment, except during extinction training on day 4 to tag the neurons with CreERT2. Five days before extinction retrieval or spontaneous recovery, mice were injected with 4-OHT to induce ChR2 expression in activated SuM-projecting mPFC^Ext^ neurons (Supplemental Figure 12D). We observed results consistent with the above experiments (Supplemental Figure 12, E–H). Next, we verified the RAM system and found that the distribution of SuM-projecting mPFC^Ext^ neurons captured by RAM system was consistent with that of the ensembles labeled by TRAP system (Supplemental Figure 13, A–C). Optogenetic activation of these neurons induced reduced fear levels during spontaneous recovery test and place preference, indicating that extinction neurons labeled by the two approaches are also functionally consistent (Supplemental Figure 13, D and E). Together, these results define the mPFC^Ext^–SuM^Glu^ pathway as a positive affect–related pathway that preferentially supports extinction persistence. SuM-projecting mPFC^Ext^ neurons are dispensable for extinction retrieval but are required for long-term extinction persistence.

### SuM-projecting mPFC extinction ensembles exhibit a distinct molecular phenotype enriched for SIRT1.

Given the role of positive affect–encoding SuM-projecting mPFC^Ext^ ensembles in promoting extinction persistence, we next sought to define their molecular signature using single-nucleus RNA sequencing (snRNA-seq). Dimensionality reduction and unsupervised clustering of the sequencing data identified the expected main cell types present in the mPFC (Figure 7, A and B, and Supplemental Figure 14). Neuronal populations were subsequently extracted and reclustered for detailed analysis. We identified 7 neuronal subclusters, each defined by the expression of canonical marker genes consistent with previous reports (43, 44): layer 2/3 excitatory neurons (L2/3 Exc), 3 subtypes of layer 5 excitatory neurons (L5-1 through L5-3 Exc), layer 6 excitatory neurons (L6 Exc), and 2 subtypes of inhibitory neurons (Sst/Pvalb and Vip) (Figure 7, C and D). We found that the majority of ZI-projecting mPFC^Ext^ ensembles (mCherry^+^) and SuM-projecting mPFC^Ext^ ensembles (EGFP^+^) overlapped with cluster 2 (mPFC layer 5 excitatory neurons) (Figure 7E), confirming our retrograde tracing experiment. Notably, ZI- and SuM-projecting mPFC^Ext^ ensembles exhibited markedly distinct gene expression profiles. Kyoto Encyclopedia of Genes and Genomes (KEGG) pathway analysis revealed that SuM-projecting mPFC^Ext^ ensembles were predominantly enriched in pathways associated with neuronal metabolic processes and neuromodulatory signaling, whereas ZI-projecting mPFC^Ext^ ensembles exhibited enrichment in pathways involved in intracellular signaling cascades and activity-dependent structural regulation (Figure 7F). Volcano plot suggested the involvement of *Sirt1* as a key regulator for selectively enriched genes in SuM-projecting mPFC extinction ensembles (Figure 7, G and H). The target protein SIRT1, a member of histone deacetylase III family, is responsible for deacetylation of lysine in histones and the conservation of DNA in the state of transcriptionally inactive heterochromatin (45). Although SIRT1 plays a pivotal role in metabolism, immune response, life-prolonging effects, and antidepressant effects (46–48), the role of SIRT1 in fear extinction processing remains unclear. These results demonstrate that SuM-projecting extinction ensembles represent a molecularly distinct population characterized by selective SIRT1 enrichment, providing a potential substrate for extinction persistence.

### Activation of mPFC^Ext^-SuM projectors preferentially promotes extinction persistence and resistance to spontaneous recovery, accompanied by increased SIRT1 expression.

Given the enrichment of SIRT1 within SuM-projecting extinction ensembles and prior evidence linking SIRT1 to pain and chronic stress–related behavioral vulnerability (49, 50), we next asked whether manipulation of the mPFC^Ext^–SuM pathway remains effective in counteracting heightened spontaneous recovery in relapse-prone mice. Following fear conditioning, extinction, and water deprivation, a subset of mice with reduced water intake displayed heightened spontaneous recovery compared with normal-intake mice (Supplemental Figure 15). We next examined whether optogenetic activation of mPFC^Ext^ projectors during spontaneous recovery could restore long-term extinction persistence in low-intake mice (Figure 8A and Supplemental Figure 16A). Activation of mPFC^Ext^–ZI or mPFC^Ext^–SuM pathways both enhanced long-term extinction persistence in low-intake and normal-intake mice. Notably, the contribution of activating mPFC^Ext^–SuM pathway was particularly prominent, and it produced a more striking resistance effect in low-intake mice that were prone to spontaneous recovery (Figure 8, B–E, and Supplemental Figure 16B).

Activating mPFC^Ext^–ZI projection facilitated extinction during both extinction retrieval and spontaneous recovery, the latter serving as a delayed test to assess the long-term persistence of extinction memory. In contrast, activating mPFC^Ext^–SuM projection selectively attenuated spontaneous recovery without affecting early extinction retrieval. Considering the heterogeneity of functions, we speculate that their contributions differ fundamentally. Inhibition of SuM-projecting mPFC^Ext^ ensembles had no detectable effect on extinction retrieval, indicating that these neurons are not required for the immediate expression of extinction memory. The selective involvement of SuM-projecting mPFC^Ext^ ensembles during spontaneous recovery — but not early extinction retrieval — raised the possibility that their contribution to extinction memory updating may not occur during CS-driven retrieval itself. Then, we used chemogenetic approaches to activate distinct subpopulations of mPFC^Ext^ ensembles daily following extinction retrieval and discontinued activation prior to the subsequent spontaneous recovery (Figure 8F). Daily activation of SuM-projecting mPFC^Ext^ ensembles during this interval markedly attenuated spontaneous recovery, despite the absence of activation during the test itself. In contrast, applying the daily activation to ZI-projecting mPFC^Ext^ ensembles failed to reduce fear response during spontaneous recovery (Figure 8G and Supplemental Figure 16, C–G). Transcriptomic profiling revealed that *Sirt1* was selectively enriched in SuM-projecting subpopulations, suggesting that *Sirt1* might serve as a population-specific molecular handle linked to extinction persistence. SuM-projecting mPFC^Ext^ ensembles from mice following daily activation displayed elevated SIRT1 expression within SuM-projecting mPFC^Ext^ ensembles (Figure 8, H and I). These findings indicate that sustained engagement of SuM-projecting mPFC^Ext^ neurons between retrieval sessions biases extinction memory toward long-term persistence and is associated with higher SIRT1 expression, whereas prolonged engagement of ZI-projecting mPFC^Ext^ neurons alone does not confer resistance to fear relapse. Together, these results demonstrate that extinction expression and extinction persistence are supported by distinct prefrontal projection pathways operating at different temporal phases: mPFC^Ext^–ZI projectors are required for the expression of extinction whenever extinction memory is available, whereas mPFC^Ext^–SuM projectors selectively shape the long-term persistence of extinction memory. Furthermore, although extinction expression can occur independently of affective engagement, the long-term persistence of extinction memory requires positive affect–associated support mediated by SuM-projecting extinction ensembles.

### SIRT1 in SuM-projecting mPFC^Ext^ ensembles regulates extinction persistence in a PTSD mouse model.

In pathological conditions such as PTSD, relapse of fear following initially successful extinction-based therapy remains a major challenge. PTSD is characterized by maladaptive fear and marked impairments in fear extinction, and extinction memories formed during exposure-based therapies often remain unstable and susceptible to spontaneous recovery in patients with PTSD (51, 52). This clinical phenomenon reflects a deficit in the stability of long-term extinction memory, a core factor that limits the enduring efficacy of exposure-based treatments. To investigate whether SIRT1 within SuM-projecting mPFC^Ext^ ensembles contributes to the regulation of long-term extinction persistence under maladaptive fear conditions, we established a PTSD mouse model using the single prolonged stress (SPS) paradigm, which consists of sequential restraint, forced swimming, and anesthesia (Figure 9A) (53–55). This paradigm recapitulates key features of extinction impairment and heightened susceptibility to spontaneous recovery in PTSD (Figure 9, B and C).

To further examine the contribution of *Sirt1* within SuM-projecting mPFC^Ext^ ensembles to extinction persistence, we performed a loss-of-function experiment selectively targeting the expression of *Sirt1* in mPFC^Ext^–SuM projectors. We employed a Cre-dependent CRISPR-Cas9 strategy combined with the RAM system (56, 57), which tags extinction ensembles in a temporally specific manner using a Dox-dependent Tet-Off system to selectively disrupt SIRT1 in SuM-projecting mPFC^Ext^ ensembles in Cas9 mice (Figure 9D). We introduced Retro-AAV-RAM-TRE-Cre into SuM, AAV delivery of *Sirt1*-targeting single guide RNAs (sgRNAs) or a control AAV containing scramble guide RNAs into mPFC of Cas9 mice (Figure 9E). AAV delivery of *Sirt1*-targeting guide RNAs significantly reduced SIRT1 expression in SuM-projecting mPFC^Ext^ ensembles compared with a control AAV containing scramble guide RNAs (Figure 9F), ensuring the conditional knockout (cKO) efficiency. Then we examined the effect of *Sirt1* cKO on long-term persistence of extinction memory in the PTSD mice. *Sirt1* cKO in SuM-projecting mPFC^Ext^ ensembles markedly increased freezing level during spontaneous recovery in the PTSD mice (Figure 9G), indicating increased relapse vulnerability. Importantly, a similar increase in freezing during spontaneous recovery was also observed in nonstressed mice with *Sirt1* cKO (Supplemental Figure 17), suggesting that *Sirt1* contributes more broadly to extinction memory persistence beyond stress-induced conditions.

To explore the functional role of enhanced SIRT1 expression, we delivered an AAV allowing for Cre-dependent expression of SIRT1 in SuM-projecting mPFC^Ext^ ensembles with the RAM system (Figure 9H). SIRT1-AAV delivery resulted in a significantly higher intensity of SIRT1 immunoreactivity within SuM-projecting mPFC^Ext^ ensembles compared with animals injected with a Cre-dependent EGFP-AAV (control) (Figure 9I). AAV-mediated overexpression of SIRT1 (SIRT1-OE) within SuM-projecting mPFC^Ext^ ensembles robustly reduced freezing levels during spontaneous recovery (Figure 9J). Additionally, SIRT1-OE in the SuM-projecting mPFC neurons did not affect fear acquisition, extinction learning, or extinction retrieval (Supplemental Figure 18). Together, these bidirectional manipulations demonstrate that *Sirt1* acts as a key molecular regulator in SuM-projecting mPFC^Ext^ ensembles, mitigating spontaneous recovery and promoting the long-term persistence of extinction in the PTSD mice. Thus, molecular features enriched in SuM-projecting mPFC^Ext^ ensembles provide a mechanistic substrate through which positive affective experience supports long-term extinction persistence even in a PTSD mouse model.

## Discussion

Exposure-based therapy for PTSD relies on fear extinction, yet relapse remains a major clinical challenge. Gaining insights into the neurological mechanisms underlying fear extinction and its persistence may help address relapse vulnerability in PTSD and guide strategies to enhance the durability of therapeutic effects. This study identifies a positive affect–associated cortical-hypothalamic mechanism that selectively supports extinction persistence, rather than immediate expression. We identified a subpopulation of extinction-tagged neurons (SuM-projecting mPFC^Ext^ ensembles) that is selectively engaged by positive affect during extinction and promotes resistance to spontaneous recovery without directly participating in the expression of extinction. This subpopulation is anatomically, genetically, and functionally distinct from other retrieval-related extinction neurons (ZI-projecting mPFC^Ext^ ensembles), which primarily regulate extinction expression. These findings indicate that extinction failure reflects disruption of a positive affective stabilization process, rather than retrieval impairment alone. Moreover, SIRT1 was selectively enriched within SuM-projecting mPFC^Ext^ ensembles, and bidirectional manipulation of SIRT1 within this pathway altered relapse behavior in a PTSD mouse model, identifying SIRT1 as a molecular regulator of extinction persistence. Together, our study suggests that affect-associated prefrontal-hypothalamic circuitry and its molecular determinants may represent potential therapeutic targets for reducing relapse vulnerability in anxiety and trauma-related disorders (Figure 10).

We performed a variety of viral- and transgenic-based approaches to visualize the downstream target regions of mPFC^Ext^ ensemble. The antegrade tracing results show that mPFC^Ext^ ensembles send extensive long-range excitatory projections to multiple brain regions associated with memory and emotional processing. Consistent with this projection architecture, pathway-defined subpopulations exhibited distinct functional properties, supporting specialized contributions to extinction behavior. These behavioral features were mediated through discrete neural pathways, which are engaged by both environmental and physiological factors to promote behavioral manifestations. Furthermore, RV-assisted retrograde tracing indicated that distinct mPFC^Ext^ populations shared certain common but distinguishable input sources, supporting that mPFC^Ext^ ensembles likely integrate multiple signals to contribute to behavioral outcomes. We hypothesize that certain mPFC^Ext^ neurons have the ability to track various conditions brought about by environmental changes by selectively enhancing the input signals to a specific target while weakening those to other targets. These highlight projection-specific organization within the mPFC^Ext^ ensemble as a key determinant of extinction persistence and relapse vulnerability. In conclusion, the mPFC^Ext^ ensemble functions as an integrative hub, where distinct mPFC^Ext^ subsets process different contents of fear extinction.

Specific experiences or associative memories often contain intrinsic affective component, which can modulate both expression and persistence of perception (58–60). The excessive reinforcement of negative emotional memory represented by conditioned fear memory can not only gradually develop into pathological memory and lead to various emotional disorders, but also perpetuate pain perception and shape pain experience (61). In the case of fear extinction memory, previous studies have shown that it is not merely an inhibitory memory but a newly formed reward memory (10, 11). By contrast, the 2 subsets of mPFC ensemble described here are not interchangeable at the behavioral level. What we believe to be the critical novel finding that we present is functional heterogeneity of mPFC ensemble in fear extinction processing. This observation extends prior work linking extinction to reward-related circuits by demonstrating that positive affective engagement is selectively routed through specific subpopulations within extinction ensembles.

The classical mesolimbic dopamine system, including the ventral tegmental area (VTA) and nucleus accumbens (NAc), has a well-established role in encoding reward, reinforcement, and unpredicted outcomes (62, 63). However, we observed extremely sparse projections from mPFC extinction ensemble in these 2 brain regions. This counterintuitive phenomenon suggests that mesolimbic pathways are not the primary route through which affective signals support extinction memory stability. Here, we identified a key subcortical region in the hypothalamus, the SuM, which plays a critical role in the novelty signal and homeostatic states (64). Previous studies have demonstrated that SuM neurons govern neurogenesis, spatial memory, and locomotion by manipulating hippocampal plasticity (65–67) and through reciprocal connections with rostral areas such as the infralimbic cortex and anterior cingulate cortex to achieve integration of cognitive and emotional aspects of goal-directed behaviors (68). In this study, we unveil a novel function of SuM neurons to modulate positive affect during fear extinction. These results suggest that positive affective modulation of extinction persistence can be dissociated from canonical mesolimbic reward circuits and instead mediated through alternative hypothalamic pathways.

We found SuM-projecting mPFC^Ext^ neurons are engaged during long-term extinction retrieval and preferentially support long-term extinction stability. These results contribute to enriching an emerging concept of memory maintenance and updating, in which positive emotions enhance and stabilize remote memory. Due to synaptic plasticity and homeostatic demands, the function of neural substrates in memory and intrinsic affective processing is experience dependent and dynamic in both short and long periods (69, 70). Our findings indicate that activation of the mPFC^Ext^–SuM pathway during long-term extinction retrieval engages a memory updating process. The positive affective experience–driven synaptic plasticity may reshape the fear-related ensembles with negative emotional connotations. In addition, this study reveals the differential contributions of distinct mPFC^Ext^ projections to the long-term extinction performance. The mPFC^Ext^–ZI pathway facilitates extinction memory expression by regulating the explicit behavioral phenotype during retrieval, while the mPFC^Ext^-SuM pathway introduces an affective context that promotes consolidation-like processes supporting extinction stability. Rather than forming parallel pathways with opposing functions, our results support a hierarchical organization in which different projection-defined ensembles contribute at distinct stages and timescales of extinction processing. Notably, the spontaneous recovery levels of low-intake mice are markedly higher than those of normal mice, and activation of the mPFC^Ext^–SuM can effectively reverse the susceptibility to spontaneous recovery, highlighting the decisive role of positive affective component in the condition of individual differences in long-term extinction persistence. This suggests that future intervention strategies could reduce susceptibility to fear relapse by reconstructing or enhancing positive valence pathways. Strengthening positive affect–associated pathways may therefore represent a mechanistically grounded approach to improving extinction persistence in PTSD.

Molecular processes triggered by experience are reengaged to stabilize and update memory traces following retrieval, reflecting an ongoing requirement for transcription- and protein synthesis-dependent signaling in the maintenance and modification of long-lasting memories (71, 72). *Sirt1* is well positioned to support long-term behavioral adaptation by regulating transcriptional and epigenetic programs associated with cellular plasticity (73). The selective enrichment within SuM-projecting mPFC^Ext^ ensembles suggests that extinction persistence may depend on molecular mechanisms that enable sustained changes in neuronal function, rather than on repeated engagement of retrieval-related circuitry alone. Importantly, *Sirt1* may represent one component of a broader molecular program within SuM-projecting mPFC^Ext^ ensembles that biases extinction toward persistence and resistance to relapse, as demonstrated in a PTSD mouse model. Future studies dissecting downstream targets of *Sirt1* within this pathway may further clarify how affective and transcriptional processes interact to support extinction persistence and mitigate relapse in PTSD.

In summary, our study identifies a previously unrecognized functional dissociation within mPFC extinction ensembles, providing a framework to understand how positive affective processes shape the vulnerability of adaptive memory. These findings refine prevailing models of fear extinction by demonstrating that successful extinction expression does not necessarily guarantee extinction persistence. Our study further unravels the neural mechanism by which the brain forms unified extinction representations through the coordination of diverse attributes, provides insight into relapse vulnerability in anxiety- or trauma-related disorders and suggests potential strategies for intervening in extinction dysfunction.

## Methods

### Sex as a biological variable.

Sex was not considered as a biological variable. Our study examined both male and female animals, and no significant differences were found between sexes.

### Animals.

The following animals were used in this study: C57BL/6J mice, Fos^2A-iCreER^ (FosTRAP2) mice (stock no. 030323, The Jackson Laboratory, USA), SST-Flp mice (stock no. 028579, The Jackson Laboratory, USA), Vglut2-Flp mice (stock no. 030212, The Jackson Laboratory, USA), PV-Flp mice (stock no. 022730, The Jackson Laboratory, USA), CRISPR/Cas9 knock-in mice (stock no. 024857, The Jackson Laboratory, USA), and the lox-stop-lox-H2B-GFP (H2B-GFP^flox^) reporter mice. All mice were group-housed on a 12-hour light/dark cycle with rodent chow and water ad libitum. Adult male and female mice (7–12 weeks old) were used for all experiments.

Detailed information on materials and methods is provided in Supplemental Methods and Supplemental Table 1.

### Statistics.

The data were collected and processed randomly. All behavioral tests and analyses were blindly conducted. Data distributions were tested for normality and variance equality among groups was assessed using Levene’s test. Data are presented as the mean ± SEM unless indicated otherwise. Statistical comparisons were performed using 2-tailed unpaired or paired Student’s *t* tests as well as 1-way ANOVAs or 2-way repeated-measures ANOVAs, where appropriate. For post hoc analysis, we used Tukey’s or Šidák’s multiple-comparison test. *P* values less than 0.05 are considered statistically significant.

### Study approval.

All procedures were conducted in accordance with the *Guide for the Care and Use of Laboratory Animals* (National Academies Press, 2011) and with the approval of the Institutional Animal Care and Use Committee at the Shanghai General Hospital of Shanghai Jiao Tong University School of Medicine (2023AW007).

### Data availability.

All data needed to evaluate the conclusions of the present study are present in the main paper and/or the supplement. Source data for this study are also available in the Supporting Data Values file. All custom code used in this study has been deposited at Zenodo: https://doi.org/10.5281/zenodo.18604679 Any additional information required for reanalyzing the reported data is available from the corresponding author upon request. Single-nucleus RNA-seq data have been uploaded at Gene Expression Omnibus (GEO: GSE319177).

## Author contributions

XG, LNH, TLX, ZJL, and XRW conceived the project, designed the experiments, and interpreted the results. ZJL, XG, and XRW performed the majority of behavioral experiments, animal surgery, immunohistochemistry, and data analysis. MYW and XL assisted with some of the behavioral experiments and data analysis. ZKL and YJW did single-nucleus RNA-seq preparation and analyses. WGL commented on data interpretation. XG, ZJL, LNH, and TLX wrote the manuscript with contributions from all authors. All authors read and approved the final manuscript.

## Conflict of interest

The authors have declared that no conflict of interest exists.

## Funding support

- National Natural Science Foundation of China (32300843 to XG).
- Shanghai Sailing Program of Science and Technology Commission of Shanghai Municipality (23YF1433900 to XG).
- STI2030-Major Projects of China (2021ZD0202800 to TLX).

## Supplementary Material

Supplemental data

Supporting data values

## Figures and Tables

**Figure 1:**
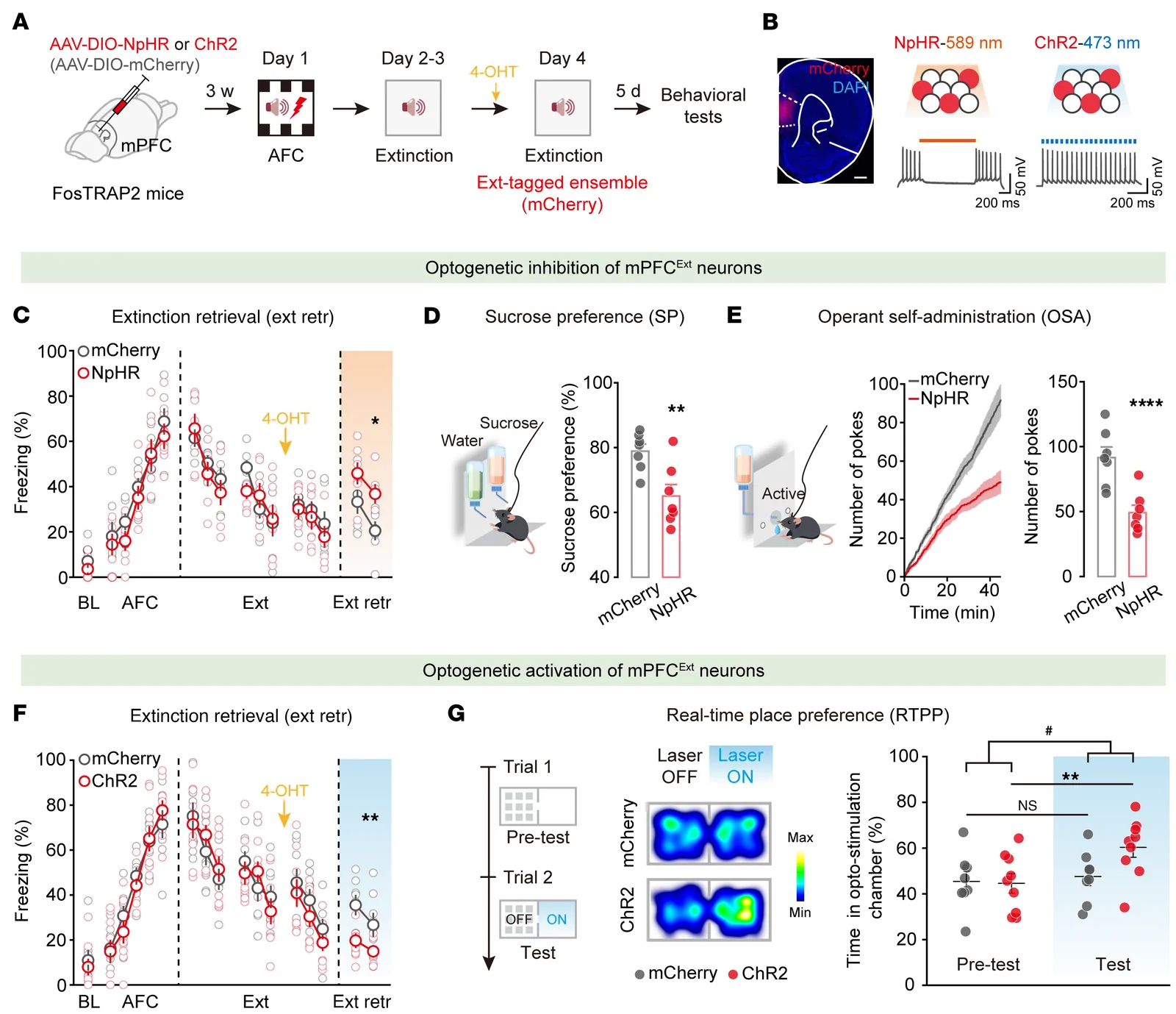
Extinction-tagged mPFC ensembles coordinate extinction retrieval and positive affective experience. (**A**) Schematics of experimental design. Experimental design for mice subject to auditory fear conditioning (AFC), extinction training (Ext), and subsequent behavioral tests. Optogenetic manipulation was delivered during behavioral tests. (**B**) Representative image of mCherry expression. Scale bar: 500 μm. Functional identification of NpHR and ChR2 expressed in mPFC mCherry^+^ neurons. (**C**) Effects of inhibiting extinction ensembles on the time courses of freezing responses to the CS during extinction retrieval (Ext Retr). Statistics are as follows: main effect of AAV, conditioning, *F*_1,12_ = 0.6965, *P* = 0.4203; extinction training, *F*_1,12_ = 0.432, *P* = 0.5234; extinction retrieval, *F*_1,12_ = 6.502, *P* = 0.0255. *n =* 7 mice per group. (**D**) Illustration of sucrose preference (SP) test and the percentage of preference for sucrose. *n =* 7 mice per group. (**E**) Illustration of operant self administration (OSA) test and cumulative curves of the number of active nose pokes. Number of nose pokes. *n =* 7 mice per group. (**F**) Effects of stimulating extinction ensembles on Ext Retr. Time courses of freezing responses to the CS. Statistics are as follows: main effect of AAV, conditioning, *F*_1,15_ = 0.0345, *P* = 0.8552; extinction training, *F*_1,15_ = 0.1378, *P* = 0.7157; extinction retrieval, *F*_1,15_ = 8.857, *P* = 0.0094. mCherry group, *n =* 8 mice; ChR2 group, *n =* 9 mice. (**G**) Illustration of real-time place preference (RTPP) test and heatmaps of the locomotor activity and the percentage of time in opto-stimulation chamber. NS, no significant difference, **P < 0.01, paired Student’s *t* test, ^#^*P* < 0.05, interaction 2-way repeated measures ANOVA. mCherry group, *n =* 8 mice; ChR2 group, *n =* 9 mice. Data are mean ± SEM. *P < 0.05, **P < 0.01. Two-way repeated measures ANOVA (**C** and **F**) and unpaired Student’s *t* test (**D** and **E**).

**Figure 2:**
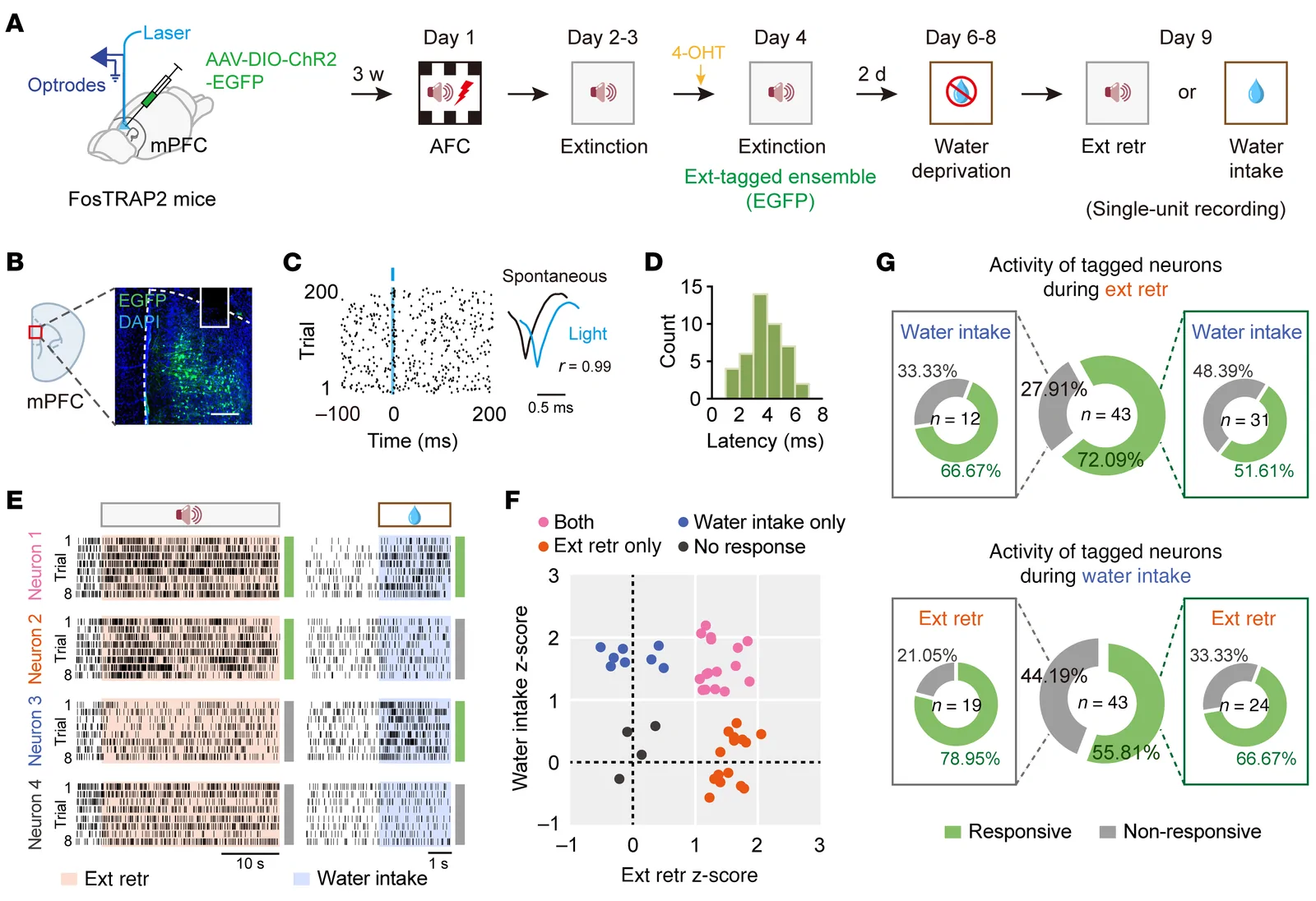
Activity heterogeneity within extinction-tagged ensemble during positive affective experience. (**A**) Schematics of experimental design. Experimental design for mice subject to auditory fear conditioning (AFC), extinction training (Ext), water deprivation, and subsequent extinction retrieval (Ext. Retr.) or water intake. (**B**) Representative image of virus expression. Scale bar: 200 μm. (**C**) Raster plot of representative tagged extinction ensembles. Light-evoked spike waveforms (blue) were similar to spontaneous ones (black). Pearson’s correlation, *r* = 0.99. (**D**) Plot showing short latencies of light-evoked spike for all ChR2-tagged mPFC neurons. (**E**) Raster plots of sample neurons in response to ext. retr. and water intake. Each example neuron (marked in different colors) represents a subtype of ChR2-tagged mPFC neurons. (**F**) Scatterplot of z-score during ext. retr. and water intake in 4 subtypes. *n =* 43 neurons from 19 mice. (**G**) Proportions of tagged extinction neurons activated by ext. retr. and water intake. Insert: proportions of ext. retr.–responsive neurons or nonresponsive neurons activated during water intake and water intake–responsive neurons or nonresponsive neurons activated during ext. retr.

**Figure 3:**
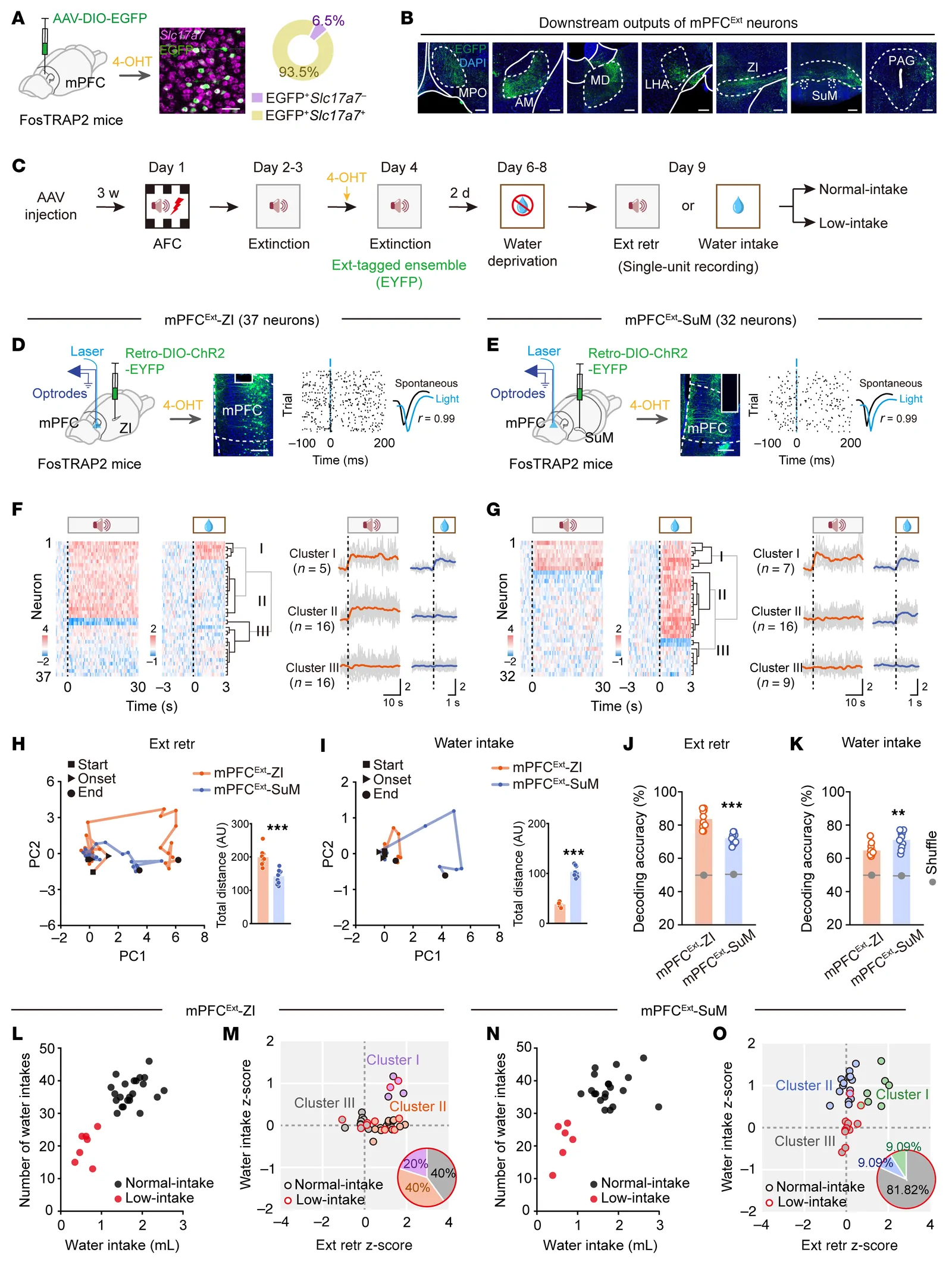
ZI- and SuM-projecting mPFC extinction ensembles respectively encode safety-associated CS and positive affective experience. (**A**) Schematics of experimental design and representative image showing EGFP expression predominantly colocalized with *Slc17a7* (vesicular glutamate transporter 1, VGLUT1). Scale bar: 50 μm. (**B**) Projection targets of mPFC extinction ensembles. Scale bars: 200 μm. (**C**) Schematics of experimental design. (**D** and **E**) Schematics of stereotaxic surgery and representative images of virus expression. Scale bars: 200 μm. Raster plot of representative tagged extinction ensembles. Light-evoked spike waveforms (blue) were similar to spontaneous ones (black). Pearson’s correlation, *r* = 0.99. (**F** and **G**) Three different clusters were identified using hierarchical clustering and average z-score trace of neural responses to ext. retr. and water intake in ChR2-tagged ZI-projecting (**F**) or SuM-projecting (**G**) mPFC extinction ensembles. (**H** and **I**) The trajectories of trial-by-trial population neuronal responses to Ext Retr. (**H**) and water intake (**I**). Insert, neural trajectories of ZI-projecting and SuM-projecting mPFC extinction ensembles in the principal-component analysis (PCA) subspace reveal differences in trajectory distance during Ext Retr and Water intake. (**J** and **K**) Performance of LDA decoders using neuronal responses to Ext Retr (**J**) and Water intake (**K**). (**L** and **N**) Unsupervised *K*-means clustering of water consumption behavior reveals 2 distinct subgroups of mice. (**L**) Normal-intake group, *n =* 22 mice; Low-intake group, *n =* 7 mice. (**N**) Normal-intake group, *n =* 20 mice; Low-intake group, *n =* 6 mice. (**M** and **O**) Scatterplot of z-score during Ext Retr and water intake in 3 clusters of ChR2-tagged ZI-projecting (**M**) or SuM-projecting (**O**) mPFC extinction ensembles. Pie charts summarize the cluster distribution of the recorded neurons in low-intake mice. Data are mean ± SEM. **P < 0.01, ***P < 0.001. Unpaired Student’s *t* test (**H**–**K**).

**Figure 4:**
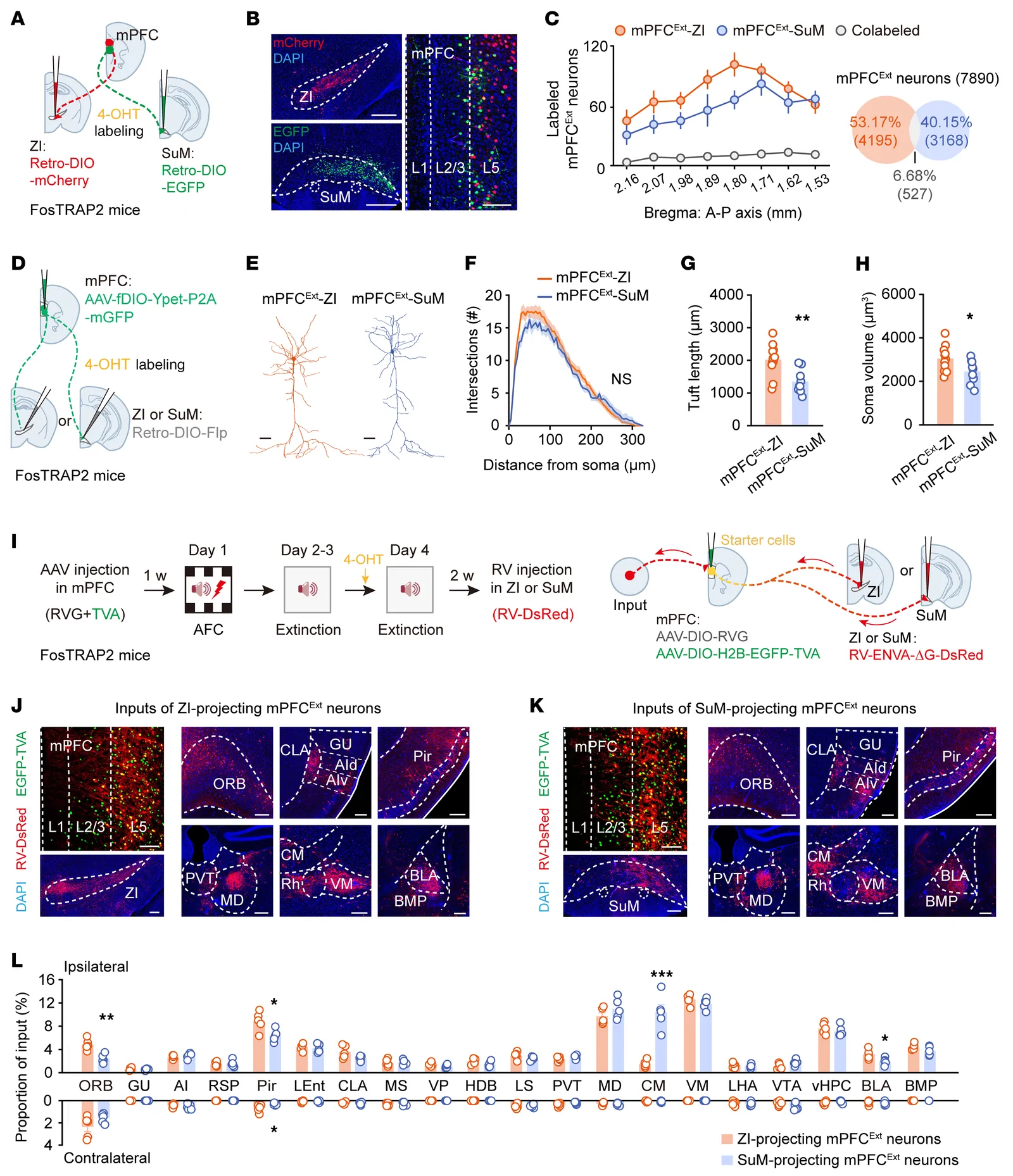
Anatomically segregated ZI- and SuM-projecting mPFC extinction ensembles show distinct morphological properties and upstream inputs. (**A** and **D**) Schematics of experimental design and AAV injections. (**B**) Representative images showing mCherry and EGFP expression. Scale bars: 500 μm. Representative image of retrograde labeling of mPFC extinction ensembles. Scale bar: 200 μm. (**C**) Number and distribution of ZI or SuM-projecting mPFC extinction ensembles. (**E**) Representative examples of reconstructed mPFC^Ext^-ZI and mPFC^Ext^-SuM neurons. Scale bars: 50 μm. (**F**) Sholl analysis of the dendritic complexity. *n =* 11 neurons in mPFC^Ext^-ZI group and *n =* 9 neurons in mPFC^Ext^-SuM group. (**G** and **H**) Apical tuft length (**G**) and soma volume (**H**) of mPFC^Ext^-ZI and mPFC^Ext^-SuM neurons. (**I**) Schematics of experimental design for RV tracing. (**J** and **K**) Representative images of TVA-EGFP and RV-DsRed expression in mPFC and RV-DsRed expression in ZI (**J**) or SuM (**K**). Representative images of the main upstream inputs of mPFC extinction ensembles. Scale bars: 200 μm. (**L**) Distribution of RV-DsRed–labeled neurons. ORB, orbitofrontal cortex; GU, gustatory area; AI, agranular insular cortex; RSP, retrosplenial cortex; Pir, piriform cortex; LEnt, lateral entorhinal cortex; CLA, claustrum; MS, medial septal nucleus; VP, ventral pallidum; HDB, nucleus of the horizontal limb of the diagonal band; LS, lateral septal nucleus; PVT, paraventricular thalamic nucleus; CM, central medial nucleus of the thalamus; VM, ventromedial thalamic nucleus; VTA, ventral tegmental area; vHPC, ventral hippocampus; BLA, basolateral amygdala; BMP, basomedial amygdaloid nucleus, posterior part. *n =* 5 mice per group. Data are mean ± SEM. *P < 0.05, **P < 0.01, ***P < 0.001. Two-way repeated measures ANOVA (**F**) and unpaired Student’s *t* test (**G**, **H**, and **L**).

**Figure 5:**
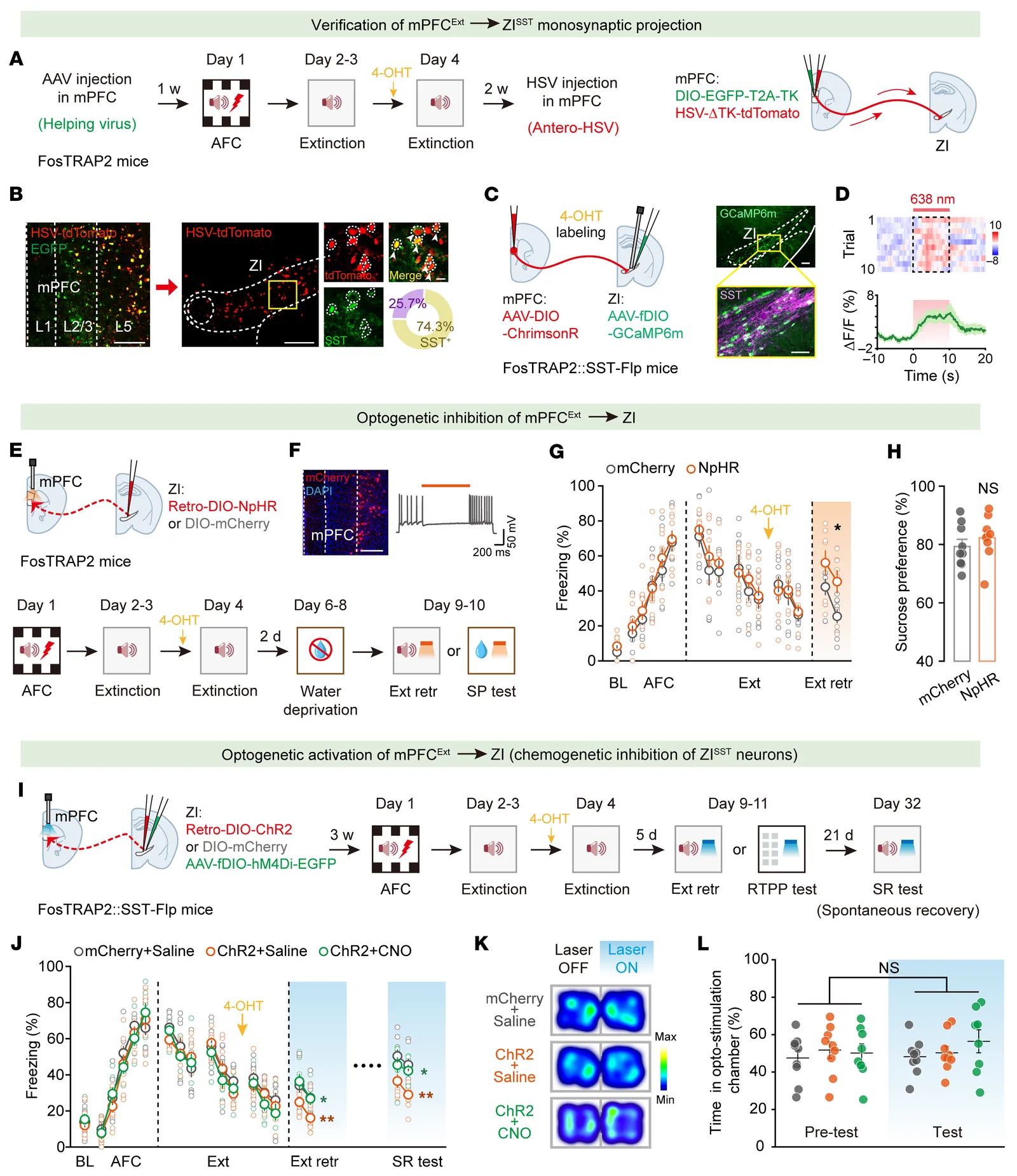
Manipulation of the mPFC^Ext^–ZI^SST^ pathway regulates the expression of extinction memory without altering positive affect–related behavior. (**A** and **I**) Schematics of experimental design. (**B**) HSV-tdTomato expression and SST immunofluorescence in ZI. Scale bars: 200 μm (low magnification) and 20 μm (high magnification). Quantification of colocalization of HSV-tdTomato and SST antibody. (**C**) Schematic of viral injection for fiber photometry. Representative images of GCaMP6m expression and SST immunofluorescence. Scale bars: 200 μm (low magnification) and 100 μm (high magnification). (**D**) Representative heatmaps and averaged fluorescence increased in response to optogenetic stimulation in ZI SST neurons. (**E**) Schematics of experimental design. (**F**) Representative image of mCherry expression in mPFC and functional validation for optogenetic inhibition. Scale bar: 200 μm. (**G**) Effects of inhibiting mPFC^Ext^-ZI projectors on ext. retr. statistics are as follows: main effect of AAV, extinction retrieval, *F*_1,16_ = 4.981, *P* = 0.0403. *n =* 9 mice per group. (**H**) Effects of inhibiting mPFC^Ext^-ZI projectors on sucrose preference. *n =* 9 mice per group. (**J**) Effects of activating mPFC^Ext^–ZI projectors on extinction retrieval and spontaneous recovery. Statistics are as follows: mCherry+Saline versus ChR2+Saline, extinction retrieval, *F*_1,16_ = 9.046, *P* = 0.0083; ChR2+Saline versus ChR2+CNO, extinction retrieval, *F*_1,15_ = 7.300, *P* = 0.0164. mCherry+Saline versus ChR2+Saline, spontaneous recovery, *F*_1,16_ = 14.46, *P* = 0.0016; ChR2+Saline versus ChR2+CNO, spontaneous recovery, *F*_1,15_ = 6.794, *P* = 0.0198. mCherry+Saline group, *n =* 9 mice; ChR2+Saline group, *n =* 9 mice; ChR2+CNO group *n* = 8 mice. (**K** and **L**) Effects of activating mPFC^Ext^–ZI projectors on RTPP. Heatmaps of the locomotor activity (**K**) and the percentage of time in opto-stimulation chamber (**L**). mCherry+Saline group, *n =* 9 mice; ChR2+Saline group, *n =* 9 mice; ChR2+CNO group = 8 mice. Data are mean ± SEM. *P < 0.05, **P < 0.01. Two-way repeated measures ANOVA (**G**, **J**, and **L**) and unpaired Student’s *t* test (**H**).

**Figure 6:**
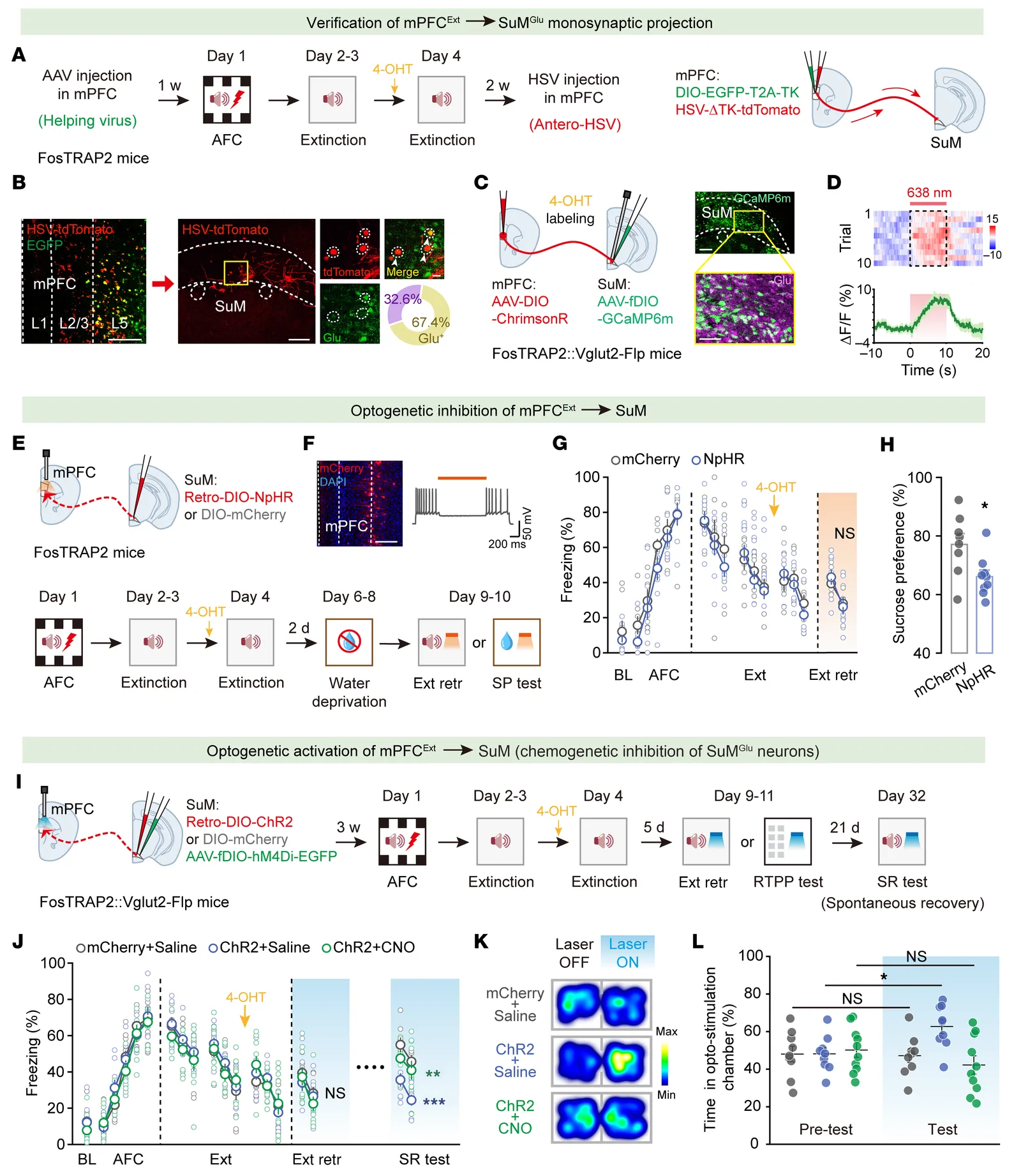
Manipulation of the mPFC^Ext^–SuM^Glu^ pathway regulates positive affect–related behavior and extinction persistence without affecting extinction retrieval. (**A** and **I**) Schematics of experimental design. (**B**) HSV-tdTomato expression and glutamate immunofluorescence in SuM. Scale bars: 200 μm (low magnification) and 20 μm (high magnification). Quantification of colocalization of HSV-tdTomato and glutamate antibody. (**C**) Schematic of viral injection for fiber photometry. Representative images of GCaMP6m expression and glutamate immunofluorescence. Scale bars: 200 μm (low magnification) and 100 μm (high magnification). (**D**) Representative heatmaps and averaged fluorescence increased in response to optogenetic stimulation in SuM glutaminergic neurons. (**E**) Schematics of experimental design. (**F**) Representative image of mCherry expression in mPFC and functional validation for optogenetic inhibition. Scale bar: 200 μm. (**G**) Effects of inhibiting mPFC^Ext^-SuM projectors on ext. retr. statistics are as follows: main effect of AAV, extinction retrieval, *F*_1,15_ = 0.0359, *P* = 0.8523. mCherry group, *n =* 8 mice, NpHR group, *n =* 9 mice. (**H**) Effects of inhibiting mPFC^Ext^-SuM projectors on sucrose preference. mCherry group, *n =* 8 mice; NpHR group, *n =* 9 mice. (**J**) Effects of activating mPFC^Ext^–SuM projectors on extinction retrieval and spontaneous recovery. Statistics are as follows: mCherry+Saline versus ChR2+Saline, extinction retrieval, *F*_1,16_ = 0.5645, *P* = 0.4634; ChR2+Saline versus ChR2+CNO, extinction retrieval, *F*_1,17_ = 0.0081, *P* = 0.9294. mCherry+Saline versus ChR2+Saline, spontaneous recovery, *F*_1,16_ = 24.33, *P* = 0.0002; ChR2+Saline versus ChR2+CNO, spontaneous recovery, *F*_1,17_ = 14.92, *P* = 0.0012. mCherry+Saline group, *n =* 9 mice; ChR2+Saline group, *n =* 9 mice; ChR2+CNO group = 10 mice. (**K** and **L**) Effects of activating mPFC^Ext^–SuM projectors on RTPP. Heatmaps of the locomotor activity (**K**) and the percentage of time in opto-stimulation chamber (**L**). mCherry+Saline group, *n =* 9 mice; ChR2+Saline group, *n =* 9 mice; ChR2+CNO group = 10 mice. Data are mean ± SEM. *P < 0.05, **P < 0.01, ***P < 0.0001. Two-way repeated measures ANOVA (**G** and **J**), unpaired Student’s *t* test (**H**), and paired Student’s *t* test (**L**).

**Figure 7:**
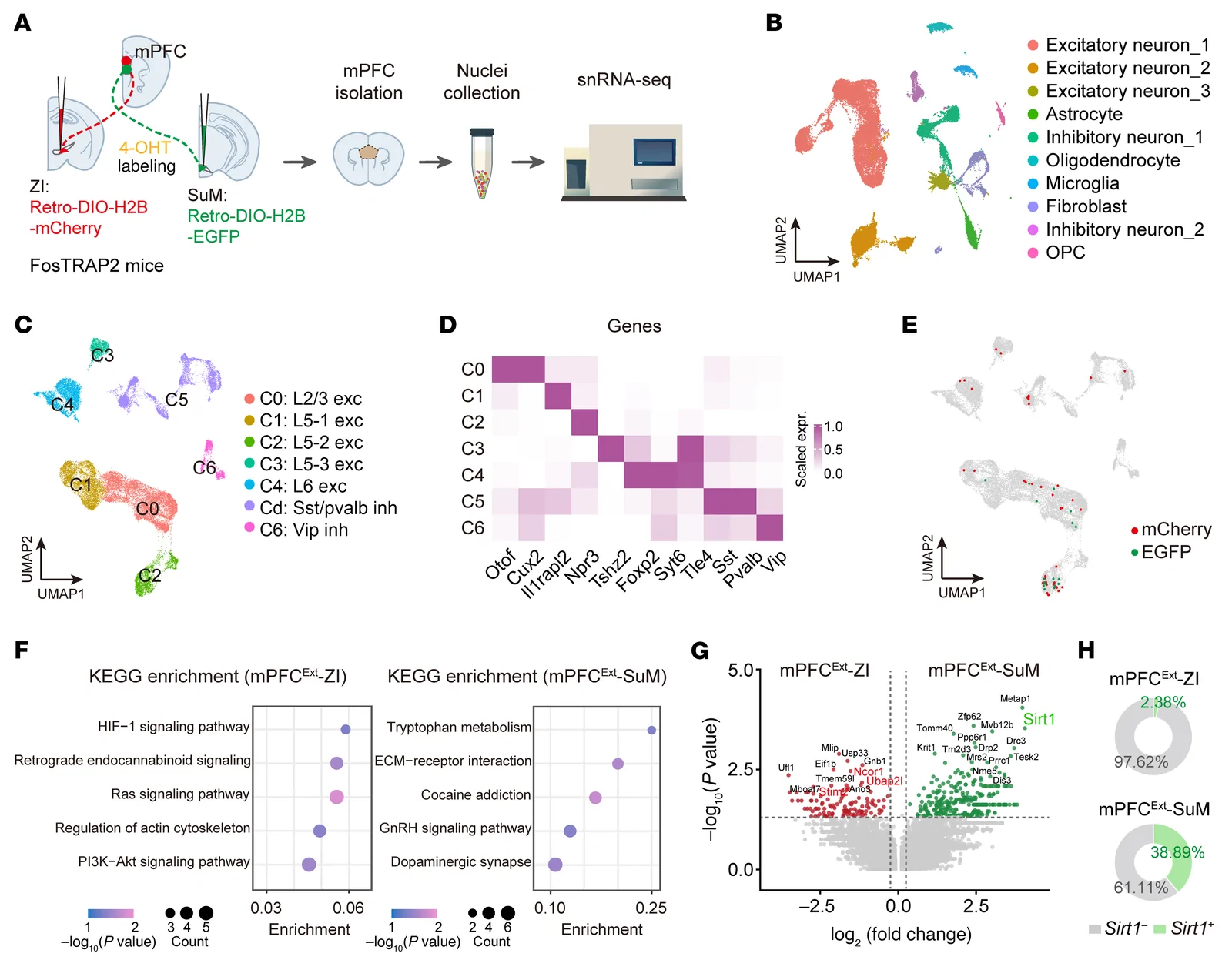
SuM-projecting mPFC extinction ensembles exhibit a distinct molecular phenotype enriched for SIRT1. (**A**) Schematic representation of the experimental design for projection-based snRNA-seq. (**B**) Uniform manifold approximation and projection (UMAP) visualization of nuclei from snRNA-seq with cell-type information. (**C**) UMAP visualization of individual nuclei corresponding to different neuronal cell types. (**D**) Heatmap showing the expression of representative cell-type marker genes. (**E**) Distribution of ZI-projecting mCherry^+^ (red dots) and SuM-projecting EGFP^+^ (green dots) neurons shown on UMAP plot. (**F**) Enriched KEGG pathways of mPFC^Ext^–SuM projectors and mPFC^Ext^–ZI projectors. (**G**) Volcano plot illustrating differentially expressed genes between ZI and SuM-projecting mPFC extinction ensembles. (**H**) Pie charts showing a higher proportion of *Sirt1*^+^ within SuM-projecting mPFC^Ext^ ensembles compared to ZI-projecting mPFC^Ext^ ensembles.

**Figure 8:**
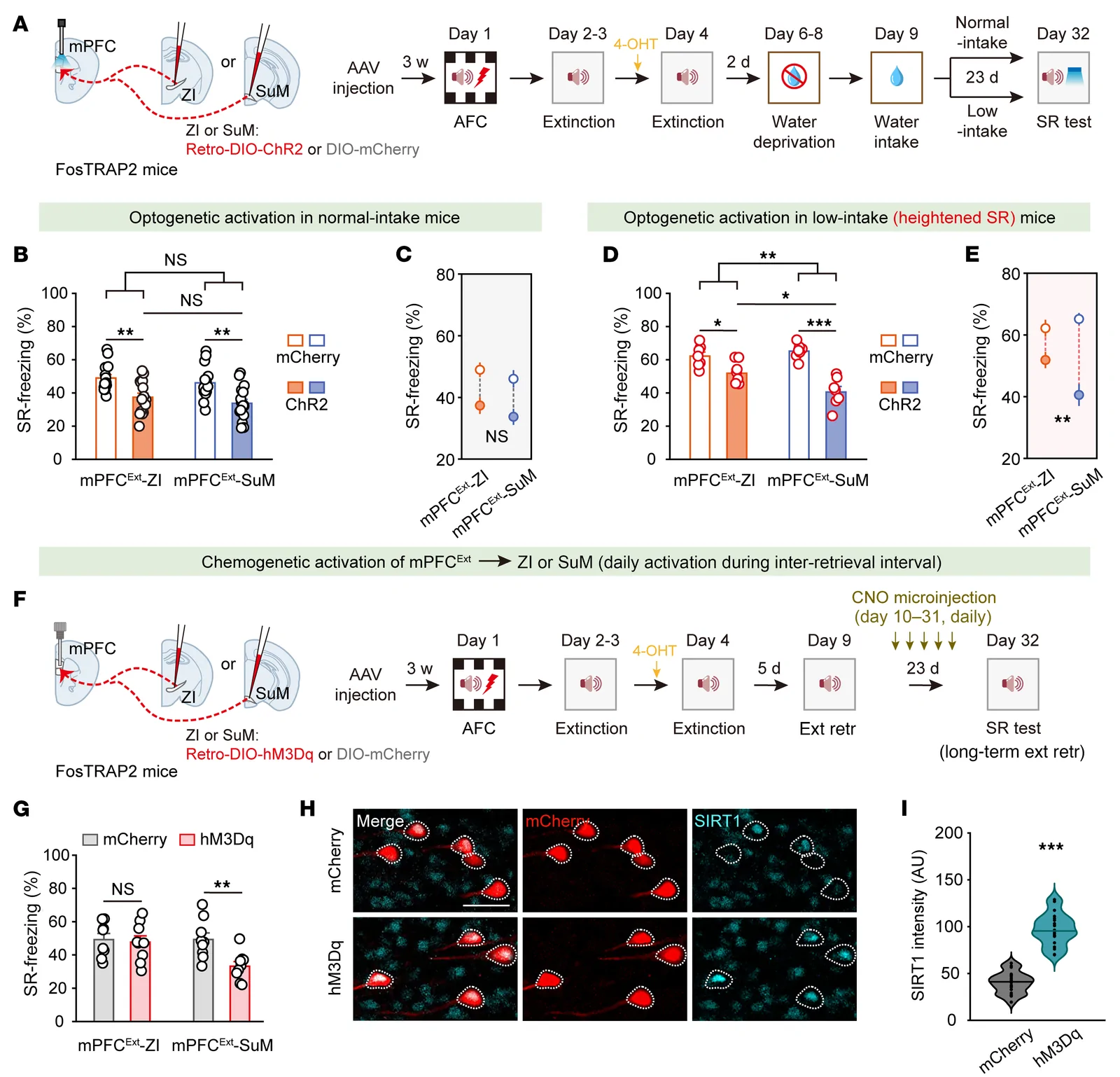
Activation of mPFC^Ext^–SuM projectors preferentially promotes extinction persistence and resistance to spontaneous recovery, accompanied by increased SIRT1 expression. (**A** and **F**) Schematics of AAV injections and schematics of experimental design. CNO was administered daily during days 10–31. No CNO was administered on the day of the SR test (Day 32). (**B** and **C**) Effects of activating mPFC^Ext^–ZI or mPFC^Ext^–SuM projectors on SR test in normal-intake mice. Statistics are as follows: region × virus *F*_1,59_ = 0.02248, *P* = 0.8813. mPFC^Ext^–ZI: mCherry group, *n =* 15 mice; ChR2 group, *n =* 16 mice. mPFC^Ext^–SuM: mCherry group, *n =* 15 mice; ChR2 group, *n =* 17 mice. (**D** and **E**) Effects of activating mPFC^Ext^–ZI or mPFC^Ext^–SuM projectors on SR test in low-intake mice. Statistics are as follows: region × virus *F*_1,25_ = 8.053, *P* = 0.0089. mPFC^Ext^–ZI: mCherry group, *n =* 7 mice; ChR2 group, *n =* 8 mice. mPFC^Ext^–SuM: mCherry group, *n =* 7 mice; ChR2 group, *n =* 7 mice. (**G**) Effects of chemogenetic activating mPFC^Ext^–ZI or mPFC^Ext^–SuM projectors on SR test. mPFC^Ext^–ZI: mCherry group, *n =* 8 mice; hM3Dq group, *n =* 9 mice. mPFC^Ext^–SuM: mCherry group, *n =* 9 mice; hM3Dq group, *n =* 9 mice. (**H** and **I**) Effects of daily chemogenetic activation of mPFC^Ext^–SuM projectors on SIRT1 expression in SuM-projecting mPFC^Ext^ neurons during SR test. Representative images (**H**) and quantification of SIRT1 intensity (**I**). Scale bar: 50 μm. *n =* 20 sections from 5 mice per group. Data are mean ± SEM. *P < 0.05, **P < 0.01, ***P < 0.001. Two-way ANOVA with Tukey’s multiple-comparison test (**B** and **D**), 2-way ANOVA (**C** and **E**) and unpaired Student’s *t* test (**G** and **I**).

**Figure 9:**
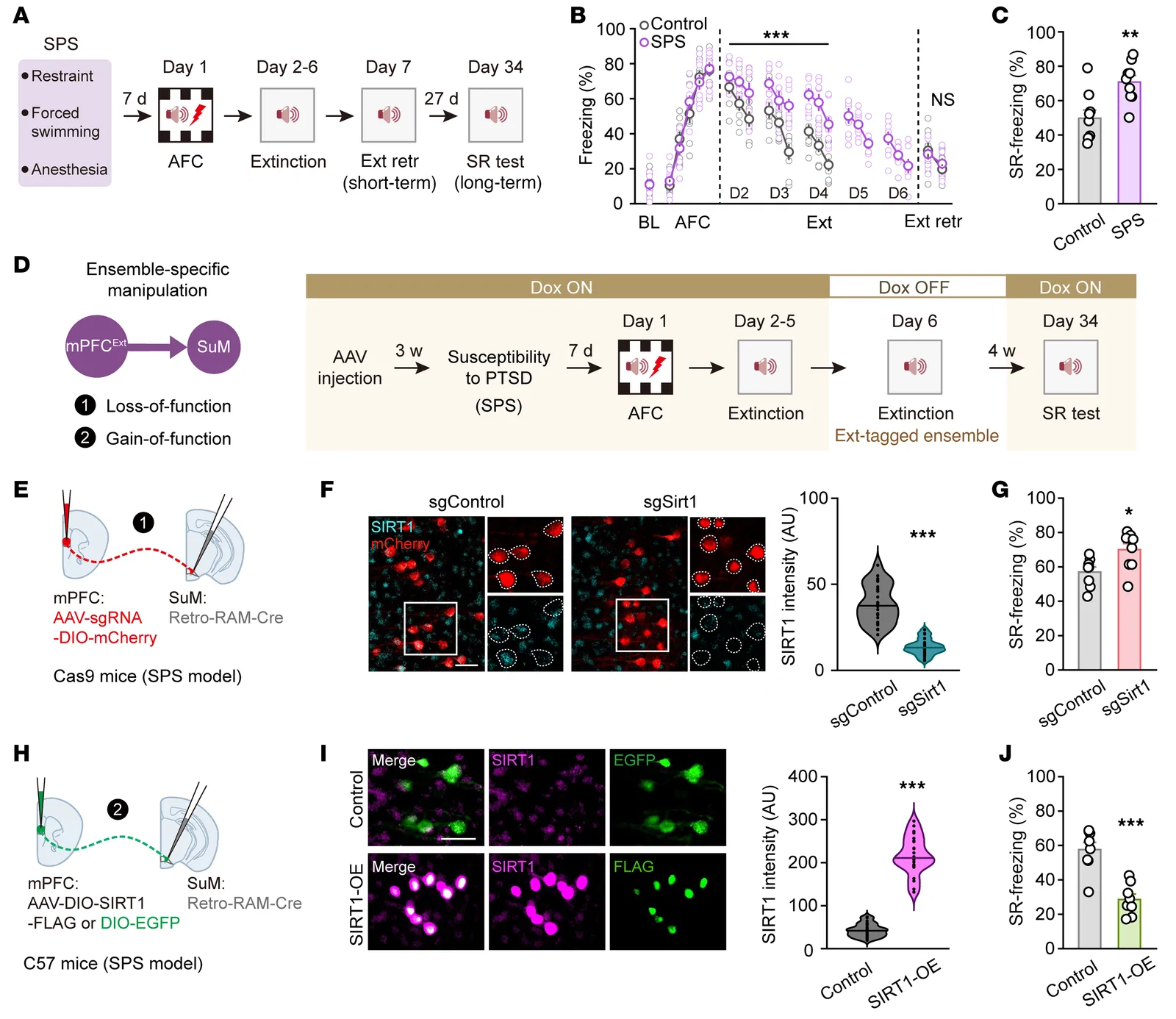
SIRT1 in SuM-projecting mPFC^Ext^ ensembles regulates long-term extinction persistence in a PTSD mouse model. (**A**) Schematics of single prolonged stress (SPS), auditory fear conditioning, extinction training, and subsequent extinction retrieval. (**B**) Time courses of freezing responses to the CS. Statistics are as follows: main effect of treatment, conditioning, *F*_1,16_ = 0.003887, *P* = 0.9511; extinction training (Day 2–Day 4), *F*_1,16_ = 65.41, *P* < 0.0001; extinction retrieval, *F*_1,16_ = 0.02955, *P* = 0.8657. *n =* 9 mice per group. (**C**) Plots showing SR freezing level between normal mice and SPS mice. (**D**) Schematics of experimental design. SuM-projecting mPFC neurons active during extinction training were tagged using the robust activity marking (RAM) system. (**E**) Schematics of AAV bilateral injection and loss-of-function experiment in the PTSD mice. (**F**) Representative images and quantification of SIRT1 intensity in the SuM-projecting mPFC^Ext^ neurons. Scale bar: 50 μm. *n =* 24 sections from 6 mice per group. (**G**) Effects of CRISPR-Cas9–mediated conditional knockout of *Sirt1* in the SuM-projecting mPFC^Ext^ neurons on SR test. sgControl group, *n =* 8 mice; sgSirt1 group, *n =* 9 mice. (**H**) Schematics of AAV injection and gain-of-function experiment in the PTSD mice. (**I**) Representative images and quantification of SIRT1 overexpression efficiency in the SuM-projecting mPFC^Ext^ neurons. Scale bar: 50 μm. *n =* 24 sections from 6 mice per group. (**J**) Effects of AAV-mediated overexpression of SIRT1 in the SuM-projecting mPFC^Ext^ neurons on SR test. Control group, *n =* 8 mice; SIRT1-OE group, *n =* 8 mice. Data are mean ± SEM. *P < 0.05, **P < 0.01, ***P < 0.001. Two-way repeated measures ANOVA (**B**) and unpaired Student’s *t* test (**C**, **F**, **G, I**, and **J**).

**Figure 10:**
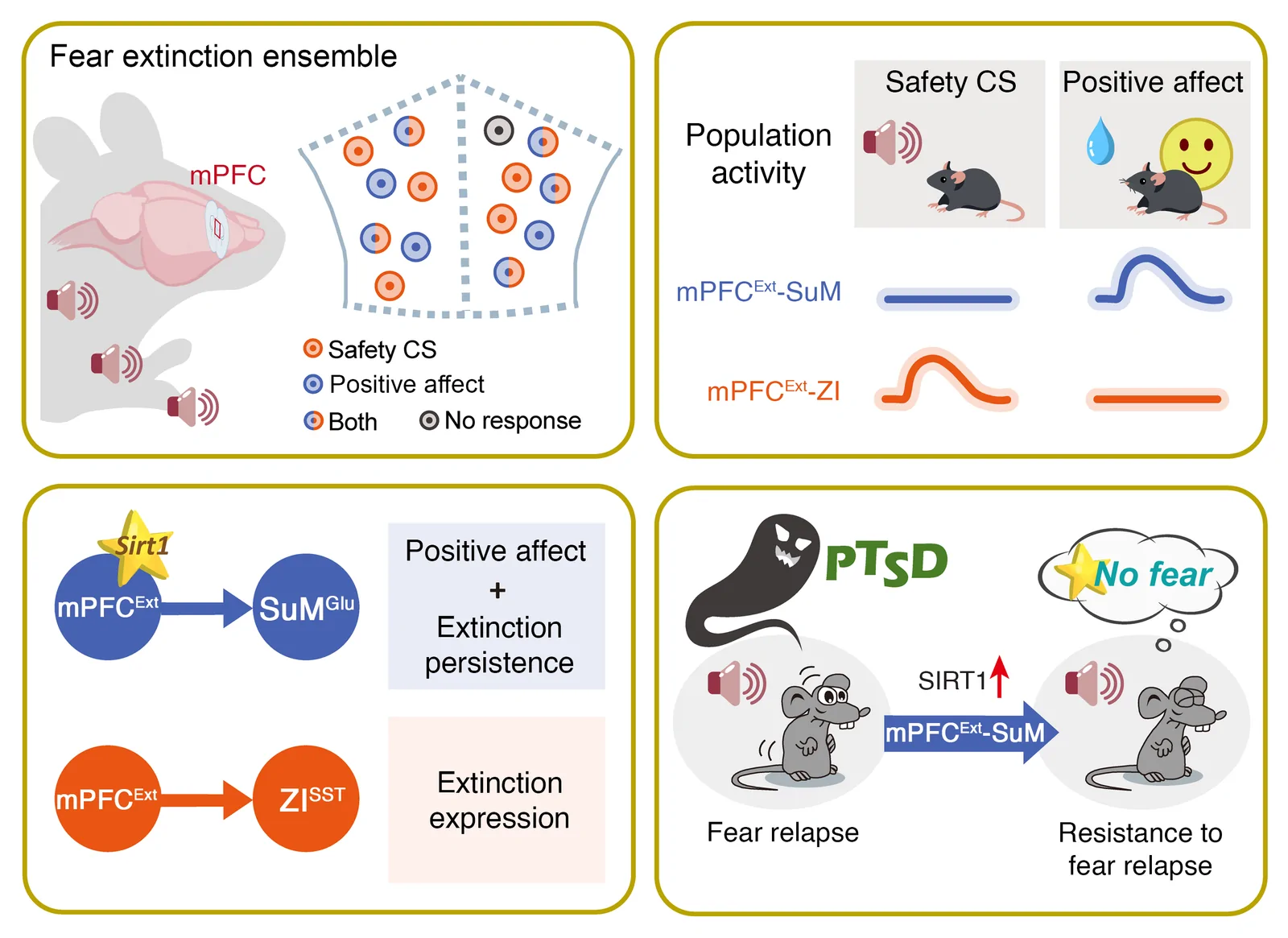
Scheme for an extinction-recruited prefrontal-hypothalamic pathway that encodes positive affect to sustain fear extinction and prevent relapse in a PTSD mouse model. This positive affect–associated circuit-molecular axis may provide a basis for therapeutic modulation to enhance extinction persistence and mitigate relapse vulnerability in trauma-related disorders.

## Version 1 08/04/2026

In-Press Preview

## Version 2 10/15/2026

Electronic publication

## References

[1] Ressler KJ (2022). Post-traumatic stress disorder: clinical and translational neuroscience from cells to circuits. Nat Rev Neurol.

[2] Liu Y (2024). Memory trace for fear extinction: fragile yet reinforceable. Neurosci Bull.

[3] Vervliet B (2013). Fear extinction and relapse: state of the art. Annu Rev Clin Psychol.

[4] Lee H, Kaang BK (2023). How engram mediates learning, extinction, and relapse. Curr Opin Neurobiol.

[5] van Kleef RS (2022). Neural basis of positive and negative emotion regulation in remitted depression. Neuroimage Clin.

[6] Erdmann T (2024). Amygdala reactivity, antidepressant discontinuation, and relapse. JAMA Psychiatry.

[7] Saito Y (2025). Amygdalo-cortical dialogue underlies memory enhancement by emotional association. Neuron.

[8] Dunsmoor JE (2015). Rethinking extinction. Neuron.

[9] Hyde J (2024). Extinction of negative conditioned stimulus valence in human fear conditioning. Behav Res Ther.

[10] Zhang X (2020). Amygdala reward neurons form and store fear extinction memory. Neuron.

[11] Zhang X (2025). Dopamine induces fear extinction by activating the reward-responding amygdala neurons. Proc Natl Acad Sci U S A.

[12] Sierra-Mercado D (2011). Dissociable roles of prelimbic and infralimbic cortices, ventral hippocampus, and basolateral amygdala in the expression and extinction of conditioned fear. Neuropsychopharmacology.

[13] Maren S (2013). The contextual brain: implications for fear conditioning, extinction and psychopathology. Nat Rev Neurosci.

[14] Euston DR (2012). The role of medial prefrontal cortex in memory and decision making. Neuron.

[15] Arnsten AF, Rubia K (2012). Neurobiological circuits regulating attention, cognitive control, motivation, and emotion: disruptions in neurodevelopmental psychiatric disorders. J Am Acad Child Adolesc Psychiatry.

[16] Otis JM (2017). Prefrontal cortex output circuits guide reward seeking through divergent cue encoding. Nature.

[17] Wang Y (2025). Integrating reproductive states and social cues in the control of sociosexual behaviors. Cell.

[18] Barbas H, Zikopoulos B (2007). The prefrontal cortex and flexible behavior. Neuroscientist.

[19] Ährlund-Richter S (2019). A whole-brain atlas of monosynaptic input targeting four different cell types in the medial prefrontal cortex of the mouse. Nat Neurosci.

[20] Lacagnina AF (2019). Distinct hippocampal engrams control extinction and relapse of fear memory. Nat Neurosci.

[21] Gu X (2022). Dynamic tripartite construct of interregional engram circuits underlies forgetting of extinction memory. Mol Psychiatry.

[22] Guenthner CJ (2013). Permanent genetic access to transiently active neurons via TRAP: targeted recombination in active populations. Neuron.

[23] DeNardo LA (2019). Temporal evolution of cortical ensembles promoting remote memory retrieval. Nat Neurosci.

[24] Sotres-Bayon F, Quirk GJ (2010). Prefrontal control of fear: more than just extinction. Curr Opin Neurobiol.

[25] Giustino TF, Maren S (2015). The role of the medial prefrontal cortex in the conditioning and extinction of fear. Front Behav Neurosci.

[26] Bouton ME (2021). Behavioral and neurobiological mechanisms of pavlovian and instrumental extinction learning. Physiol Rev.

[27] Anastasiades PG, Carter AG (2021). Circuit organization of the rodent medial prefrontal cortex. Trends Neurosci.

[28] Kim SY (2013). Diverging neural pathways assemble a behavioural state from separable features in anxiety. Nature.

[29] Nieh EH (2016). Inhibitory input from the lateral hypothalamus to the ventral tegmental area disinhibits dopamine neurons and promotes behavioral activation. Neuron.

[30] Schroeder A (2023). Inhibitory top-down projections from zona incerta mediate neocortical memory. Neuron.

[31] Tao C (2023). The medial preoptic area mediates depressive-like behaviors induced by ovarian hormone withdrawal through distinct GABAergic projections. Nat Neurosci.

[32] Maren S, Quirk GJ (2004). Neuronal signalling of fear memory. Nat Rev Neurosci.

[33] Cho JH (2013). Synaptic encoding of fear extinction in mPFC-amygdala circuits. Neuron.

[34] Li H (2022). Neurotensin orchestrates valence assignment in the amygdala. Nature.

[35] Yang T (2023). Plastic and stimulus-specific coding of salient events in the central amygdala. Nature.

[36] Morais-Silva G, Lobo MK (2024). Refining the circuits of drug addiction: the ventral pallidum. Curr Opin Neurobiol.

[37] Ahmadlou M (2021). A cell type-specific cortico-subcortical brain circuit for investigatory and novelty-seeking behavior. Science.

[38] Zhu M (2025). Transcriptomic and spatial GABAergic neuron subtypes in zona incerta mediate distinct innate behaviors. Nat Commun.

[39] Kolmac C, Mitrofanis J (1999). Distribution of various neurochemicals within the zona incerta: an immunocytochemical and histochemical study. Anat Embryol (Berl).

[40] Bouton ME (2006). Contextual and temporal modulation of extinction: behavioral and biological mechanisms. Biol Psychiatry.

[41] Myers KM, Davis M (2007). Mechanisms of fear extinction. Mol Psychiatry.

[42] Golbabaei A (2026). PV-dependent reorganization of prelimbic cortex sub-engrams during systems consolidation. Neuron.

[43] Lui JH (2021). Differential encoding in prefrontal cortex projection neuron classes across cognitive tasks. Cell.

[44] Muir J (2024). Isolation of psychedelic-responsive neurons underlying anxiolytic behavioral states. Science.

[45] Singh V, Ubaid S (2020). Role of silent information regulator 1 (SIRT1) in regulating oxidative stress and inflammation. Inflammation.

[46] Knight JR, Milner J (2012). SIRT1, metabolism and cancer. Curr Opin Oncol.

[47] Lei Y (2020). SIRT1 in forebrain excitatory neurons produces sexually dimorphic effects on depression-related behaviors and modulates neuronal excitability and synaptic transmission in the medial prefrontal cortex. Mol Psychiatry.

[48] Abdulkhaliq AA (2025). SIRT1 in aging and diseases. J Cell Biochem.

[49] Zhang XL (2021). Difference of pain vulnerability in adult and juvenile rodents: the role of SIRT1-mediated ClC-3 trafficking in sensory neurons. Pain.

[50] Shlomi S (2022). Imbalance in Sirt1 alternative splicing in response to chronic stress during the adolescence period in female mice. Int J Mol Sci.

[52] Markowitz S, Fanselow M (2020). Exposure therapy for post-traumatic stress disorder: factors of limited success and possible alternative treatment. Brain Sci.

[53] Bienvenu TCM (2021). The advent of fear conditioning as an animal model of post-traumatic stress disorder: learning from the past to shape the future of PTSD research. Neuron.

[54] Dunsmoor JE (2022). Laboratory models of post-traumatic stress disorder: the elusive bridge to translation. Neuron.

[55] Lin ZJ (2024). Stimulation of an entorhinal-hippocampal extinction circuit facilitates fear extinction in a post-traumatic stress disorder model. J Clin Invest.

[56] Sørensen AT (2016). A robust activity marking system for exploring active neuronal ensembles. Elife.

[57] Lesuis SL (2025). Stress disrupts engram ensembles in lateral amygdala to generalize threat memory in mice. Cell.

[58] Ramirez S (2015). Activating positive memory engrams suppresses depression-like behaviour. Nature.

[59] Corder G (2019). An amygdalar neural ensemble that encodes the unpleasantness of pain. Science.

[60] Oswell CS (2026). Mimicking opioid analgesia in cortical pain circuits. Nature.

[61] Stegemann A (2023). Prefrontal engrams of long-term fear memory perpetuate pain perception. Nat Neurosci.

[62] Russo SJ, Nestler EJ (2013). The brain reward circuitry in mood disorders. Nat Rev Neurosci.

[63] Morales M, Margolis EB (2017). Ventral tegmental area: cellular heterogeneity, connectivity and behaviour. Nat Rev Neurosci.

[64] Kesner AJ (2023). Hypothalamic supramammillary control of cognition and motivation. J Neurosci.

[65] Chen S (2020). A hypothalamic novelty signal modulates hippocampal memory. Nature.

[66] Farrell JS (2021). Supramammillary regulation of locomotion and hippocampal activity. Science.

[67] Li YD (2023). Activation of hypothalamic-enhanced adult-born neurons restores cognitive and affective function in Alzheimer’s disease. Cell Stem Cell.

[68] Pan WX, McNaughton N (2004). The supramammillary area: its organization, functions and relationship to the hippocampus. Prog Neurobiol.

[69] Tye KM (2018). Neural circuit motifs in valence processing. Neuron.

[70] Silva BA, Gräff J (2023). Face your fears: attenuating remote fear memories by reconsolidation-updating. Trends Cogn Sci.

[71] Tronson NC, Taylor JR (2007). Molecular mechanisms of memory reconsolidation. Nat Rev Neurosci.

[72] Park S (2026). Molecular mechanisms mediating engram ensemble retrievability state in mice. Neuron.

[73] Michán S (2010). SIRT1 is essential for normal cognitive function and synaptic plasticity. J Neurosci.

