# Supplementary material for: Extinction-recruited prefrontal-hypothalamic pathway encodes positive affect to sustain fear extinction in posttraumatic stress mouse model
Source: J Clin Invest. 2026 Aug 4;136(20):e206270. doi: 10.1172/JCI206270 (PMC13668643; doi:10.1172/JCI206270)
Supplement: Supplementary file 1 [file jci-136-206270-s113.pdf]

Supplemental Material for

Extinction-recruited prefrontal-hypothalamic pathway encodes positive affect to sustain fear extinction in post-traumatic stress mouse model

Ze-Jie Lin[1,2,†], Xin-Rong Wu[2,3,†], Ming-Yang Wei[2,3], Zheng-Kai Lao[2], Xiang Lan[1], Yan-Jiao Wu[2], Wei-Guang Li[2,4], Tian-Le Xu[2,5,*], Li-Na Huang[1,*], Xue Gu[1,*]

The file includes:

Supplemental Methods

Supplemental Figures 1 to 18

Supplemental Table 1

Supplemental Methods

Virus constructs.

The following viruses were used: AAV2/9-EF1α-DIO-hChR2(E123T/T159C)-mCherry, AAV2/9-EF1α-DIO-eNpHR3.0-mCherry, AAV2/9-EF1α-DIO-mCherry, AAV2/9-hSyn-DIO-hChR2(H134R)-EGFP, AAV2/9-hSyn-DIO-EGFP, AAV2/R-hSyn-Flp, AAV2/9-EF1α-CreOn-FlpOn-GCaMP6s, AAV2/9-hSyn-CreOn-FlpOn-EYFP, AAV2/R-EF1α-DIO-mCherry, AAV2/R-EF1α-DIO-EGFP, AAV2/R-EF1α-DIO-ChR2(H134R)-EYFP, AAV2/R-EF1α-DIO-Flp, AAV2/9-EF1α-fDIO-Ypet-P2A-mGFP, AAV2/9-EF1α-DIO-RVG, AAV2/9-EF1α-DIO-H2B-EGFP-TVA, RV-ENVA-ΔG-DsRed, AAV2/9-EF1α-DIO-EGFP-T2A-TK, HSV-ΔTK-LSL-tdTomato, AAV2/9-nEF1α-fDIO-hM4Di-EGFP, AAV2/R-hSyn-DIO-H2B-EGFP, AAV2/R-hSyn-DIO-H2B-mCherry, AAV2/9-TH-Flp, AAV2/9-RAM-ERT2CreERT2, AAV2/R-EF1α-DIO-hM3Dq-mCherry, AAV2/R-hSyn-Cre (all purchased from Brain VTA, Wuhan, China); AAV2/9-nEF1α-fDIO-GCaMP6m, AAV2/9-hSyn-DIO-ChrimsonR-mCherry, AAV2/R-EF1α-DIO-eNpHR3.0-mCherry, AAV2/R-EF1α-DIO-ChR2(H134R)-mCherry, and AAV2/R-RAM-d2TTA-TRE-Cre (from Brain Case, Shenzhen, China); AAV2/9-(H1-sgRNA.sp(mSIRT1))x3-CAG-DIO-mCherry and AAV2/9-H1-sgRNA.sp(NC)-CAG-DIO-mCherry (from Taitool Bioscience, Shanghai, China); AAV-hSyn-DIO-Sirt1-3xFLAG and AAV-hSyn-DIO-EGFP (from Obio Technology, Shanghai, China). All viruses were stored in aliquots at –80 °C until use. The viral titers for injection were >10^{12} viral particles per ml.

Stereotaxic surgery

Mice were anesthetized with 1.5–2.0% isoflurane in 100% oxygen and subsequently stabilized in a stereotactic apparatus with atraumatic ear bars (RWD Life Science), ensuring precise alignment on a digital stereotactic frame. Virus solutions were loaded into the tips of pipettes (Sutter Glass pipettes) and injected at the following coordinates (posterior to Bregma, AP; lateral to the midline, ML; below the Bregma, DV; in mm): mPFC: AP,

+1.75 mm; ML, ±0.35 mm; DV, –2.50 mm; MPO: AP, –0.20 mm; ML, ±0.25 mm; DV, –5.50 mm; AM: AP, –0.80 mm; ML, ±0.50 mm; DV, –3.60 mm; MD: AP, –1.20 mm; ML, ±0.50 mm; DV, –3.10 mm; LHA: AP, –1.40 mm; ML, ±1.20 mm; DV, –5.20 mm; ZI: AP, –1.80 mm; ML, ±1.0 mm; DV, –4.45 mm; SuM: AP, –2.80 mm; ML, ±0.50 mm; DV, –5.05 mm; PAG: AP, –3.80 mm; ML, ±0.50 mm; DV, –2.60 mm. After injection, the pipette was left in place for an additional 10 min to allow the injectant to diffuse adequately.

For fiber photometry and optogenetic experiments, ceramic fiber optic cannulas (200 μm in diameter, 0.37 numerical aperture (NA), Hangzhou Newdoon Technology) were implanted into following coordinates: mPFC: AP, +1.75 mm; ML, ±0.35 mm; DV, –2.40 mm; ZI: AP, –1.80 mm; ML, ±1.0 mm; DV, –4.35 mm; SuM: AP, –2.80 mm; ML, ±0.50 mm; DV, –4.95 mm. The ceramic fiber optic cannulas were positioned in place with acrylic dental cement and secured with skull screws.

For anterograde monosynaptic tracing, AAV-DIO-EGFP-T2A-TK was injected into the mPFC of FosTRAP2 mice. A week later, the mice received behavioral training. Two weeks later, HSV-ΔTK-tdTomato was injected into the same location. The histology experiments were performed five days after HSV injection.

For retrograde monosynaptic tracing, a 1:1 volume mixture of AAV-EF1α-DIO-RVG and AAV-EF1α-DIO-H2B-EGFP-TVA was injected into the mPFC of FosTRAP2 mice. A week later, the mice received behavioral training. Two weeks later, RV-ENVA-ΔG-DsRed was injected into the ZI or SuM area. The histology experiments were performed one week after rabies virus injection.

Fear conditioning, extinction, and memory retrieval

All auditory fear conditioning, extinction, and memory retrieval procedures were performed using the Ugo Basile Fear Conditioning System (UGO BASILE srl). Before fear conditioning, mice were handled and habituated to the conditioning chamber (17 cm × 17 cm × 25 cm) for three consecutive days. On day 1, mice were conditioned individually in context A (the chambers were equipped with stainless-steel electric shocking

grids and were cleaned with 75% ethanol; the chamber walls were covered with black-and-white checkered wallpaper) with five pure tones (CS; 4 kHz, 76 dB, 30 s each) delivered at variable intervals (20–180 s). Each tone was co-terminated with a foot shock (US; 0.75 mA, 2 s each). ANY-maze software (Stoelting Co.) was used to automatically control the delivery of tones and foot shocks. For extinction training, mice were presented with 12 CS presentations (4 kHz, 76 dB, 30 s each) without foot shock in context B (gray floor box) on days 2–4. On day 9, mice received eight CS-alone (30 s each) presentations either in context B for extinction retrieval or in context A for fear retrieval. Spontaneous recovery tests occurred 28 days after the cessation of extinction training. The movement of the mouse in chamber was recorded using a near-infrared camera and analyzed in real-time with ANY-maze software. A fear response was operationally defined as measurable behavioral freezing (more than 1-s cessation of movement), which was automatically scored and analyzed by ANY-maze software (version 7.2, Stoelting Co., USA).

Fiber photometry

The fiber photometry system (ThinkerTech, Nanjing, China) allowed for real-time excitation and recording of fluorescence from genetically encoded calcium indicators in freely moving mice. To minimize photo-bleaching, the laser power at the tip of the optical fiber was adjusted to a low level of 40–60 μW. The analog voltage signals were digitalized at 100 Hz using a Power 1401 digitizer and Spike2 software (CED, Cambridge, UK). Photometry data were further analyzed using MATLAB. The fluorescence change values ($\Delta F/F$) were calculated as $(F - F_0)/F_0$, where F_0 is the baseline fluorescence signal averaged over a 10-s time-window prior to the trigger events.

To record Ca^{2+} signals of SST neurons or Vglut2 neurons induced by optogenetic stimulation of mPFC extinction neurons, FosTRAP2::SST-Flp or FosTRAP2::Vglut2-Flp mice were injected with AAV-fDIO-GCaMP6m and AAV-DIO-ChrimsonR for expression in SST neurons or Vglut2 neurons and extinction neurons,

respectively. Recordings in SST or Vglut2 neurons were performed concurrently with ChrimsonR terminals being stimulated through the same optic fiber. Optogenetic stimulation was delivered ($\lambda = 638$ nm) at 20 Hz and fiber photometry recordings were made using an Arduino board running custom code synchronized to the photometry system.

Optogenetic manipulation

For photostimulation during behavioral assays, a 473-nm or 589-nm light-emitting diode (LED) (Hangzhou Newdoon Technology Co. Ltd) was connected to a patch cord with connectors on each end. The optic fiber implanted in the mouse was connected to the optic patch cord using ceramic mating sleeves. For the optogenetic activation experiments, blue light (473 nm, 20 Hz, 10-ms duration, 4–6 mW) was delivered during presentation of every 30-s CS or when the mice entered the stimulation chamber. For the optogenetic silencing experiments, the yellow light (589 nm, 8–10 mW) was delivered continuously.

Chemogenetic manipulation

For chemogenetic inhibition experiments, AAV-fDIO-hM4Di-EGFP was bilateral injected into ZI or SuM. After three weeks, mice were subjected to fear learning and extinction. clozapine N-oxide (CNO, 1 mg/kg) was injected intraperitoneally 30 min before behavioral tests. For chemogenetic activation experiments, Retro-DIO-hM3Dq-mCherry was injected into ZI or SuM. After three weeks to allow viral expression, CNO (1 mM) was applied into the cannulas with a microinjection pump (RWD Ltd.) at a rate of 0.2 μl/min and then an additional minute was given to allow the drug. For daily chemogenetic activation experiments, CNO was administered daily from day 10 to day 31 and was discontinued before the spontaneous recovery test.

In vivo electrophysiological recording

For in vivo optogenetic tagging of extinction neurons, FosTRAP2 mice were unilaterally injected with AAV-DIO-ChR2-EGFP at mPFC and custom-made optrodes (consisting of an optical fiber and 4 tetrodes) were implanted at the same coordinates where the virus was injected. The tip of the optic fiber was 300 μm above the tip of the electrodes. Each tetrode was made of four twisted fine platinum/iridium wires (12.5 μm diameter, California Fine Wire). Silver wires with two screws were attached to the skull as ground. After recovery from the surgery, the mice were habituated to the headstage and cables connected to the electrode on their heads for several days prior to electrophysiological recordings. Spiking activities were digitized at 30 kHz, band-pass filtered between 250 and 8000 Hz, and stored on a PC for further offline analysis.

Spike sorting and unit classification with optogenetic tagging

Data were exported to Offline Sorter (Plexon Inc.) and NeuroExplorer (Nex Technologies) for offline analysis. Spike waveforms were identified by threshold crossing and sorted into units (presumptive neurons) by principal component analysis (PCA). Waveforms with inter-spike intervals (ISIs) less than the refractory period (2 ms) were excluded. Only units showing spikes with a signal-to-noise ratio larger than 2 were considered as neurons and included. Cross-correlation histograms were plotted to ensure that no unit was discriminated against more than once on different channels.

For optical identification of mPFC extinction neurons, blue-light pulses (473 nm, 5-ms pulse duration, 1 Hz) were delivered at the end of each recording session. To assess whether these units were driven directly by ChR2 or indirectly by synaptic connections, we analyzed the onset latency relative to each light pulse. Only spikes with short spike latency (< 10 ms) and low jitter (< 3 ms) after light-pulse illumination were considered as being directly stimulated in this study. Only when the waveforms of laser-evoked and spontaneously generated spikes were highly similar (correlation coefficient >0.9), then they were considered to originate from the same unit.

Single unit firing analysis

To analyze mPFC neuronal activity during behavioral trials, z-scored peristimulus time histograms (PSTHs)

were calculated for each individual neuron. Spikes were divided into 500-ms or 100-ms bins for visualizing individual unit responses and comparing responses between groups during memory retrieval or water intake, respectively. A z-score was calculated for each bin relative to the prestimulus activity by subtracting average firing rate during baseline and by dividing the difference by the baseline standard deviation during memory retrieval or water intake, respectively. For a given neuron, firing rates during behavioral trials and during pre-event baseline were compared across eight trials to determine the significance of firing rate difference between these two conditions (paired Student's *t* test). Neurons exhibiting a significant increase in firing rate during behavioral trials (Ext Retr or Water intake) relative to baseline ($P < 0.05$) were defined as Ext Retr- or Water intake-responsive neurons.

Principal component analysis

To visualize event-locked dynamics (Ext Retr vs Water intake) in ZI- and SuM-projecting mPFC extinction neurons while enabling a fair cross-condition comparison, we fit a single global PCA to a global response matrix containing trial-averaged data for both ZI- and SuM- projections. For each projection, spike counts were trial-averaged in 0.5-s bins. To equalize dimensionality across groups, we randomly subsampled units from the larger group to match the smaller group on each iteration. For PCA fitting, averaged pathway-specific response matrices were concatenated along the unit dimension to yield a global response matrix. PCA was applied and the first two eigenvectors were extracted. Event- and pathway-specific low-dimensional trajectories were obtained by projecting the corresponding peri-event data segments onto the extracted eigenvectors. For each trajectory, total path length was computed as the mean of Euclidean distances between successive time bins in the 2-D PC space. The entire procedure was repeated 100 times and results were averaged to yield one estimate. For statistical comparison, only a random half of trials were included when constructing pseudopopulation activity for each response type. This process was repeated ten times to perform statistical comparisons across

ZI- and SuM- projecting groups. For visualization purposes, representative PCA trace was obtained by directly applying PCA on the global response matrix as previously described, without subsampling trials or unit counts.

Decoding analysis

We evaluated the separability of neural representations across conditions using linear discriminant analysis (LDA) implemented in MATLAB (fitcdiscr). We set DiscrimType='pseudoLinear', which fits an LDA classifier with a shared covariance matrix across classes and computes linear decision boundaries without regularization.

For each projection pathway (ZI- and SuM-projecting mPFC extinction neurons) and stimulus type (Ext Retr, Water intake), we constructed peri-event feature vectors by binning spike counts in 0.5-s windows and concatenating time bins within each trial. A LDA decoder was trained to discriminate post-stimulus activity from baseline. A four-fold cross-validation was then performed to assess the decoder's performance.

To enable fair decoding accuracy comparison between ZI- and SuM- projecting mPFC extinction neurons, on each iteration we randomly subsampled units from the larger group to match the unit count of the smaller group. This process was repeated 100 times and results were averaged to obtain one estimate. Only a random half of trials were included when constructing pseudopopulation activity for each projection type. This process was repeated ten times to perform statistical comparisons across ZI- and SuM- projecting groups. To obtain a chance-level decoding performance, the decoding procedure was repeated with shuffled class labels.

Sucrose preference (SP) test

Mice were single housed to acclimatize to two bottles of water for 24 h, including one bottle of 2% sucrose and one bottle of pure water. Animals were water deprived before the test and then exposed to the two bottles for 1 h. Bottle positions were switched after 30 min. For optogenetic manipulations, mice received light stimulation during the whole testing period. Sucrose preference was calculated as the proportion of 2% sucrose in the total

liquid consumed (pure water and sucrose).

Operant self-administration (OSA) test

Mice were first habituated to the operant behavioral chamber (Anilab) that has two nose-poke ports (active and inactive). A drop of sucrose water was delivered from the lickometer spout in the behavioral chamber every 10 s in the habituation sessions. Mice that readily learned to lick the drops were subjected to the FR = 1 (fixed ratio = 1) training for 3 days, during which the active nose-poke would trigger the delivery of a drop of sucrose water. After 3 days on the FR1 schedule, the same cohort of mice was tested for a 45-min session. Mice were returned to free-drinking following operant training.

Real-time place preference (RTPP) test

A two-chamber apparatus was used with different visual and textural cues for the real-time place preference test. An overhead video camera was used to trace the position of the mice (Cineplex, Plexon). Mice could freely explore between the two chambers through a small opening. Mice were first put into the non-stimulation chamber. Whenever the mouse entered the other chamber, LED light was delivered until the animal exited. The total duration of each trial was 15 minutes. Mice were returned to homecage after each test session. The stimulation chamber was randomly assigned to each animal and balanced for the whole group.

Open field test

Mice were placed in an open field arena (40 cm × 40 cm × 30 cm). A video-tracking system (EthoVision 3.0, Noldus) was used to measure the locomotor activity of the animal. The total distance and time spent in the center zone (20 cm × 20 cm) were measured over 15 minutes.

Elevated plus maze test

The apparatus consisted of a central square (7 cm × 7 cm), closed arms with walls (30 cm × 7 cm × 15 cm), and two open arms without walls (30 cm × 7 cm). Mice were placed into the central area and allowed to explore for 12 minutes. The time spent in the open arms was recorded by the video-tracking system (EthoVision 3.0, Noldus).

Single prolonged stress (SPS) model

A PTSD mouse model was established using the single prolonged stress (SPS) paradigm. Briefly, the mice were restrained for 2 h in 50 mL polypropylene conical tubes with a nose hole for ventilation. Subsequently, the mice were forced to swim for 20 min in a plastic tub (20 cm diameter, 30 cm height) filled with water (24 ± 1 °C, 20 cm depth). Then, they were dried and allowed to recuperate for 15 min before being exposed to isoflurane and anesthetized until the characteristics of rapid breathing and loss of responses to toe and tail pinch were evident. After that, the mice were left undisturbed in the homecage for 7 days.

Activity-dependent neuronal labeling

Recombination was induced with 4-hydroxytamoxifen (4-OHT, Sigma-Aldrich, Catalog no. H6278). 4-OHT was dissolved at 20 mg/ml in ethanol by shaking at 37°C. Corn oil (Sigma-Aldrich, Catalog no. C8267) was then added for a final concentration of 10 mg/ml 4-OHT, and the ethanol was evaporated by vacuum under centrifugation. All injections were delivered intraperitoneally (i.p.). Activity-dependent neuronal labeling was induced by a single intraperitoneal injection of 4-OHT (50 mg/kg mice) administered before the third extinction session, thereby labeling extinction-tagged neurons. Mice were then returned to the vivarium with a regular 12 h light-dark cycle for the remainder of the experiment.

To label SuM-projecting extinction neurons, we used the robust activity marking (RAM) activity tagging

system, which is a modified Tet-Off system dependent on doxycycline (Dox). Mice were microinjected with AAV virus on a Dox diet (MedChemExpress, Catalog no. HY-N0565, 40 mg/kg in the chow). To tag neurons active during extinction, mice were removed from Dox for training.

Single nucleus RNA-sequencing

Single-nucleus dissociation

The mice were anesthetized and were performed a trans-cardiac perfusion with ice-cold N-methyl-D-glucamine-artificial cerebrospinal fluid (NMDG-ACSF) containing the following components (in mM): 93 N-methyl-D-glucamine, 2.5 KCl, 1.2 NaH_2PO_4, 30 $NaHCO_3$, 20 HEPES, 25 D-glucose, 5 sodium ascorbate, 2 Thiourea, 3 Sodium pyruvate, 10 $MgSO_4$, 11.9 N-acetyl-L-cysteine, 1 $CaCl_2$, and 6 ml HCl (pH 7.35–7.45), continually bubbled with O_2 and CO_2 (95% O_2/5% CO_2, v/v). Coronal brain slices (400 μm thick) containing regions of the mPFC were cut using a vibratome (Leica VT1000S, Germany), then promptly transferred to ice-cold NMDG-ACSF and incubated for 15 min. TTX (1 μM), CNQX (20 μM; Sigma-Aldrich, Catalog no. C239), and D-APV (100 μM; Sigma-Aldrich, Catalog no. A8054) were added in this step. The micro-dissected tissues were promptly frozen in liquid nitrogen and then stored at -80 °C until use. Frozen tissues were softened in the A buffer, and sieved through a 70 μm cell strainer. Triton X-100 was added to the sample homogenate to achieve a final concentration of 0.1%. The sample was incubated on ice for 5 minutes, and then large impurities were removed using a 40 μm cell strainer. After centrifugation, 1 mL of C Buffer was added to the precipitate, and the cell nuclei precipitate was gently resuspended. Then, 500 μL of G2 buffer was slowly added to the bottom of the LoBind Tube, and 500 μL of G1 buffer was slowly and gently added to the upper layer of the G2 buffer without disturbing the boundary liquid surface. Resuspended cell nuclei were slowly added to the G1 buffer surface. After centrifugation, the cell nuclei aggregated at the bottom of the tube. The cell nuclei were resuspended in an appropriate amount of 1 × PBS with 0.5% BSA. Then, dissociated single nuclei were stained

with AO/PI for quantity and agglomeration rate assessment using Countstar Fluorescence Cell Analyzer. The concentrations of the nuclei were adjusted to approximately 1,000 nuclei/μL for snRNA-seq.

Single-nucleus RNA sequencing

The snRNA-seq libraries were generated using the 10×Genomics Chromium Controller Instrument and Chromium Single Cell 3' V3 Reagent Kits (10×Genomics, Pleasanton, CA). Briefly, nuclei were concentrated to approximately 1000 nuclei/μL and loaded into each channel to generate single-cell Gel Bead-In-Emulsions (GEMs). After the RT step, GEMs were broken and barcoded-cDNA was purified and amplified. The amplified barcoded cDNA was fragmented, A-tailed, ligated with adaptors and index PCR amplified. The final libraries were quantified using the Qubit High Sensitivity DNA assay (Thermo Fisher Scientific) and the size distribution of the libraries was determined using a High Sensitivity DNA chip on a Bioanalyzer 2200 (Agilent). All libraries were sequenced by DNBSEQ-T7 on a 150 bp paired-end run.

snRNA-seq data processing and main cell type clustering

Raw sequencing data were processed using Cell Ranger v8.0.1 (10x Genomics). Reads were aligned to a custom mouse reference genome based on GRCm39 with default settings. The genes of EGFP and mCherry were manually added to enable detection of SuM-projecting mPFC extinction neurons or ZI-projecting mPFC extinction neurons respectively.

Downstream analysis was conducted on R server (v4.2.2). Filtered feature-barcode matrices from Cell Ranger were imported into Seurat (v5.1.0) and converted into Seurat objects. To avoid potential technical noise, poor-quality cells were removed according to the number of detected genes, the ratio of genes to UMIs and the proportion of mitochondrial transcripts in total counts. (nFeature > 200, log10(genes/UMIs) > 0.8 & percent.mt < 5%). Data were normalized using the LogNormalize method, and ~2,000 highly variable genes were identified. ScaleData function was applied to scale the expression across each matrix. Potential doublets were estimated and filtered out via DoubletFinder (v2.0.4) with sample-specific pK values determined by parameter sweeping

and expected doublet rates estimated from the number of captured nuclei. After doublet removal, all datasets were merged into an individual Seurat object and scaled again. Dimension reducing methods including PCA, UMAP, and tSNE were then conducted to observe the distribution of cells from each sample, where top 20 variable dimensions were included. Batch effects across samples were minimal, therefore no additional integration methods (e.g., Harmony) were applied to maintain the potential alterations between neurons. Cell clusters were identified by specific marker genes from FindAllClusters function. The appropriate resolution (resolution = 0.5) was determined using Clustree (v0.5.1), in which all non-neuronal cell types could be separated.

Re-clustering of neurons and visualization

Neuronal clusters were isolated, scaled, and re-clustered (resolution = 0.2), resulting in 14 neuronal clusters. According to published markers, 14 clusters were re-allocated into a refined taxonomy of cortical excitatory and inhibitory neuron subtypes (L2/3, L5-1, L5-2, L5-3, L6, Sst/Pvalb, Vip). Reporter-positive nuclei were identified by UMI detection of the EGFP and mCherry transgenes ($\geqslant$1 UMI). Differential expression analyses between EGFP$^+$ and mCherry$^+$ neurons were performed using FindMarkers function, followed by removal of predicted genes and pseudogenes based on Ensembl biotypes of org.Mm.eg.db (v3.16.0). Functional enrichment analyses were conducted using clusterProfiler (v4.6.2) with significance thresholds of $P < 0.05$, whose results were visualized by enrichplot (v1.18.4). Dimensional reduction plots, dot plots, violin plots, or feature visualization on dimensional reduction plots were drawn via DimPlot, DotPlot, VlnPlot or FeaturePlot function embedded in Seurat. Volcano plots were drawn by ggplot2 (v3.4.4). Heatmaps were drawn by Heatmap function in ComplexHeatmap (v2.15.4).

Slice electrophysiology

Mice were deeply anesthetized and subsequently decapitated. Brains were quickly dissected and chilled in ice-

cold N-methyl-D-glucamine-artificial cerebrospinal fluid (NMDG-ACSF) containing the following components (in mM): 93 N-methyl-D-glucamine, 2.5 KCl, 1.2 NaH_2PO_4, 30 $NaHCO_3$, 20 HEPES, 25 D-glucose, 5 sodium ascorbate, 2 Thiourea, 3 Sodium pyruvate, 10 $MgSO_4$, 11.9 N-acetyl-L-cysteine, 1 $CaCl_2$, and 6 ml HCl (pH 7.35–7.45), continually bubbled with O_2 and CO_2 (95% O_2/5% CO_2, v/v). Coronal brain slices (300 μm thick) containing regions were cut with a vibratome (Leica VT1000S, Germany). Slices were initially recovered for 5–10 min in NMDG-ACSF at 31 °C. They were then transferred to another ACSF solution (in mM): 125 NaCl, 2.5 KCl, 12.5 D-glucose, 1 $MgCl_2$, 2 $CaCl_2$, 1.25 NaH_2PO_4, and 25 $NaHCO_3$ (pH 7.35–7.45) for an additional 2 hours of recovery at 31 °C. The slice was subsequently transferred to a recording chamber and continuously superfused with ACSF at a rate of 1–2 ml per minute. All solutions were continually bubbled with O_2 and CO_2 (95% O_2/5% CO_2, v/v).

Neurons were patched under visual guidance using infrared differential-interference contrast microscopy (BX51WI, Olympus), and an optiMOS camera (QImaging). Whole-cell patch-clamp recordings were performed using an Axon 200B amplifier (Molecular Devices). Membrane currents and potentials were sampled and analyzed using a Digidata 1440 interface and a personal computer running Clampex and Clampfit software (pCLAMP 10.5, Molecular Devices). Optical stimulation of ChR2- or NpHR-expressing neurons was conducted using a collimated LED (Lumen Dynamics Group Inc) with peak wavelengths of 473 or 589 nm, respectively. The LED was connected to an Axon 200B amplifier to initiate photostimulation. The brain slice in the recording chamber was illuminated through a 40 × water-immersion objective lens (LUMPLFLN 40XW, Olympus). To verify the functional potency of NpHR-mediated optogenetic inhibition, yellow light was delivered to generate outward photocurrents under voltage-clamp mode, which promoted membrane hyperpolarization to reduce spikes produced by current injection under current-clamp mode. The functional potency of the ChR2-expressing virus was validated by measuring the number of action potentials evoked by using 20 Hz of blue-light stimulation. The spiking activity and membranous properties of cell populations were quantified using an

internal solution comprising the following concentrations (in mM): 145 potassium gluconate, 5 NaCl, 10 HEPES, 2 MgATP, 0.1 Na_2GTP, 0.2 EGTA, and 1 $MgCl_2$ (280–300 mOsm, pH 7.2 with KOH). Subsequent data analysis was performed using the MiniAnalysis Program (Version 6.0.1, Synaptosoft).

Sparse labeling and morphology reconstruction

For the ZI- and SuM-projecting mPFC extinction neurons sparse labeling in mPFC, 100 nl AAV-EF1a-fDIO-Ypet-P2A-mGFP (diluted to ~1 × 10^9 viral genomes per ml) was injected into the mPFC of FosTRAP2 mice. At the same time, 200 nL Retro-AAV-DIO-Flp (5 × 10^{12} viral genomes per ml) was injected into the ZI or SuM. Before the third extinction session, 4-OHT was administered, thereby labeling ZI- or SuM-projecting mPFC extinction neurons. Three weeks after the 4-OHT injection, the mice were killed and the brain tissues were processed for imaging. Brains were sectioned coronally at 300 μm thickness using a vibratome (VT1000S, Leica). Fluorescently labeled neurons were imaged using a confocal microscope (FV3000, Olympus). For three-dimensional reconstruction, z-stack images were acquired.

CRISPR/Cas9-mediated gene knockout

The CRISPR-associated endonuclease Cas9 from Streptococcus pyogenes (SpCas9) was employed to induce indels in the *Sirt1* (NC_93759). Corresponding single guide RNAs (sgRNAs) for *Sirt1* gene were designed and produced using the GRCm38 (Mus musculus) reference genome by Taitool Bioscience. Ten candidate sgRNAs were selected for *Sirt1* gene according to computed specificity and efficiency scores (CHOPCHOP). These sgRNAs were cloned into a 2-in-1 reporter vector (CRPT001, Taitool), which co-expresses a single sgRNA and a cut-activated EGFP reporter. The sgRNA target sequence was positioned ahead of the EGFP ORF within the vector. The reporter vectors were co-transfected with a Cas9-expressing plasmid into 293T cells. Functional Cas9/sgRNA complexes cut the target on the reporter vector and restored EGFP expression. The top three

sgRNAs were selected and cloned into an adeno-associated virus (AAV) vector tandemly. Three sgRNAs, together with their respective promoters and scaffold sequences, were tandemly cloned into an AAV vector. The chosen sgRNAs were as follows: 5'-CGGACGAGCCGCTCCGCAAG-3', 5'-TGGCCGGGGACGGAGACAAT-3', and 5'-CCATGGCGGCCGCCGCGGAA-3' for the *Sirt1* gene targeting exon 1 of the forward strand, exon 1 of the forward strand, and exon 1 of the reverse strand of the *Sirt1* coding region, respectively. A non-targeting negative control guide was designed with a scrambled sequence with no known targets in the genome (5'-GTATTACTGATATTGGTGGG-3'). sgRNAs for *Sirt1* was cloned into the pAAV2-(H1-sgRNA.sp(MCS))x3-CAG-DIO-mCherry-WPRE-pA vector (AC610-C, Taitool). These plasmids were co-transfected into HEK293 cells with AAV9 Cap and Rep plasmids and adenovirus helper plasmid to produce recombinant AAV vectors. Purified AAV vectors were subsequently delivered in vivo via stereotaxic injection.

In situ hybridization

Fluorescent in situ hybridization was conducted using the ACDBio V2 RNAScope kit (Advanced Cell Diagnostics). The following products were employed: RNAscope Multiplex Fluorescent Detection Reagent V2, Pretreatment Reagents, RNAscope Wash Buffer Reagents, probes for Slc17a7 (416631-C2), Slc32a1 (319191-C3), Slc17a6 (319171-C2), Pvalb (421931-C1) and Sst (404631-C2), along with the PerkinElmer TSA Plus Fluorescence Palette Kit. Mice were deeply anesthetized and transcardially perfused with 1 × PBS before being fixed in 4% paraformaldehyde for 24 hours at 4 °C. The brains were then transferred to 30% sucrose dissolved in PBS until they sank to the bottom of the container. After dehydration, brain tissue was embedded in optimal cutting temperature compound (OCT) and frozen at –80 °C. Coronal brain slices were sectioned at a thickness of 16-µm using a cryostat (Leica CM 1950). Slides were baked for 1 hour at 60 °C. Subsequently, 5–8 drops of Hydrogen Peroxide were added to cover the entire section, followed by an incubation for 10 min at room

temperature. The slides were then washed three times in distilled water. The tissues were brought to Target Retrieval Reagent and maintained for 5 min at 99 °C. Following this, the slides were washed three times in distilled water. They were then transferred to 100% alcohol for 3 min. Approximately 5 drops of Protease III were added to each section, and the samples were incubated for 30 min at 40 °C. Excess liquid was removed from the slides, and corresponding probes were added to entirely cover each slide at 40 °C for two hours. To amplify the signals, tissues were sequentially immersed in AMP1, AMP2, and AMP3 at 40 °C. AMP1 and AMP2 were incubated for 30 min each, while AMP3 was incubated for 15 min. After each incubation, the appropriate HRP channel was chosen, and 4–6 drops of HRP-C1 or HRP-C2 or HRP-C3 were added to entirely cover each slide at 40 °C for 15 min, followed by incubation with HRP blocker for 15 min at 40 °C. For a specific HRP channel, corresponding diluted Opal™ Dye was added to each slide and incubated for 30 min at 40 °C. Between all steps, slides were washed twice in 1× RNAscope wash buffer for 2 min. Subsequently, the slides were incubated in ACDBio DAPI for 10 min, washed, dried for 20 min, cover-slipped with mounting media, and left to dry overnight before imaging. Confocal images of RNAscope-labeled slices were acquired using a SLIDEVIEW microscope (VS200, Olympus).

Histology and fluorescent immunostaining

Animals were deeply anesthetized with 1% sodium pentobarbital and were transcardially perfused with 1 × PBS, followed by 4% paraformaldehyde in 1 × PBS. Brains were extracted and post-fixed overnight at 4°C in 4% paraformaldehyde and 30-μm coronal sections were collected using a vibratome (VT1000S, Leica). After a 15-min incubation in 4,6-Diamidino-2-phenylindole dihydrochloride hydrate (DAPI) solution (1: 2000), sections were washed three times (15 min each time) in PBS with 0.1% Tween-20. Slides were mounted in the dark with glass coverslips using mounting media. All fluorescent images were collected by taking serial z-stack images through 10 × or 20 × objectives of a confocal microscope (FV3000, Olympus).

For the c-Fos staining, brain slices were washed with 1 × PBS and were then blocked with 10% normal donkey serum in 1 × PBS with 0.3% Triton X-100 (PBST) for 1 h, after which they were incubated overnight at 4°C with rabbit anti-c-Fos (1:500, Cell Signaling Technology, catalog no. 2250S). Sections were then washed with PBS with 0.1% Tween-20, incubated in 2% normal donkey serum for 10 min, and then incubated for 2 h with Alexa Fluor 568 donkey anti-rabbit IgG (ThermoFisher Scientific, USA; catalog no. A10042). Sections were washed in 1 × PBS with 0.1% Tween-20, mounted onto slides, and cover slipped with ProLong Gold Antifade Mountant (Invitrogen). Quantification was performed by counting the number of c-Fos-positive cells. All counts were performed blind with respect to treatment groups.

For labeling SST, PV, TH, glutamate, SIRT1, or FLAG, mice were perfused under deep anesthesia with PBS followed by 4% PFA. The brains were post-fixed in PFA for 2 h and stored in a 30% sucrose solution for 1–2 days at 4 °C. Brain sections (40 μm) were prepared with a cryostat (CM1900, Leica). Slices were incubated in the blocking solution with goat anti-somatostatin (SST) (1:500, Santa Cruz, catalog no. sc-7819), mouse anti-parvalbumin (PV) (1:500, Sigma, catalog no. MAB1572), mouse anti-tyrosine hydroxylase (TH) (1:500, Sigma, catalog no. MAB318), rabbit anti-glutamate (1:500, Sigma, catalog no. G6642), rabbit anti-SIRT1 (1:1000, Sigma, catalog no. 07-131) or mouse anti-FLAG (1:1000, Sigma, catalog no. F1804) at 4 °C overnight and then with the secondary antibody (Alexa Fluor 488 donkey anti-goat IgG, Alexa Fluor 647 donkey anti-goat IgG, Alexa Fluor 647 donkey anti-mouse IgG, Alexa Fluor 488 donkey anti-rabbit IgG, Alexa Fluor 568 donkey anti-rabbit IgG, Alexa Fluor 647 donkey anti-rabbit IgG) or Alexa Fluor 488 donkey anti-mouse IgG (all 1:400, Thermo Fisher Scientific) for 2 h. Slices were washed in 1 × PBS with 0.1% Tween-20, mounted onto slides, and covered with coverslips with ProLong Gold Antifade Mountant (Invitrogen).

Supplemental Figures and Legends

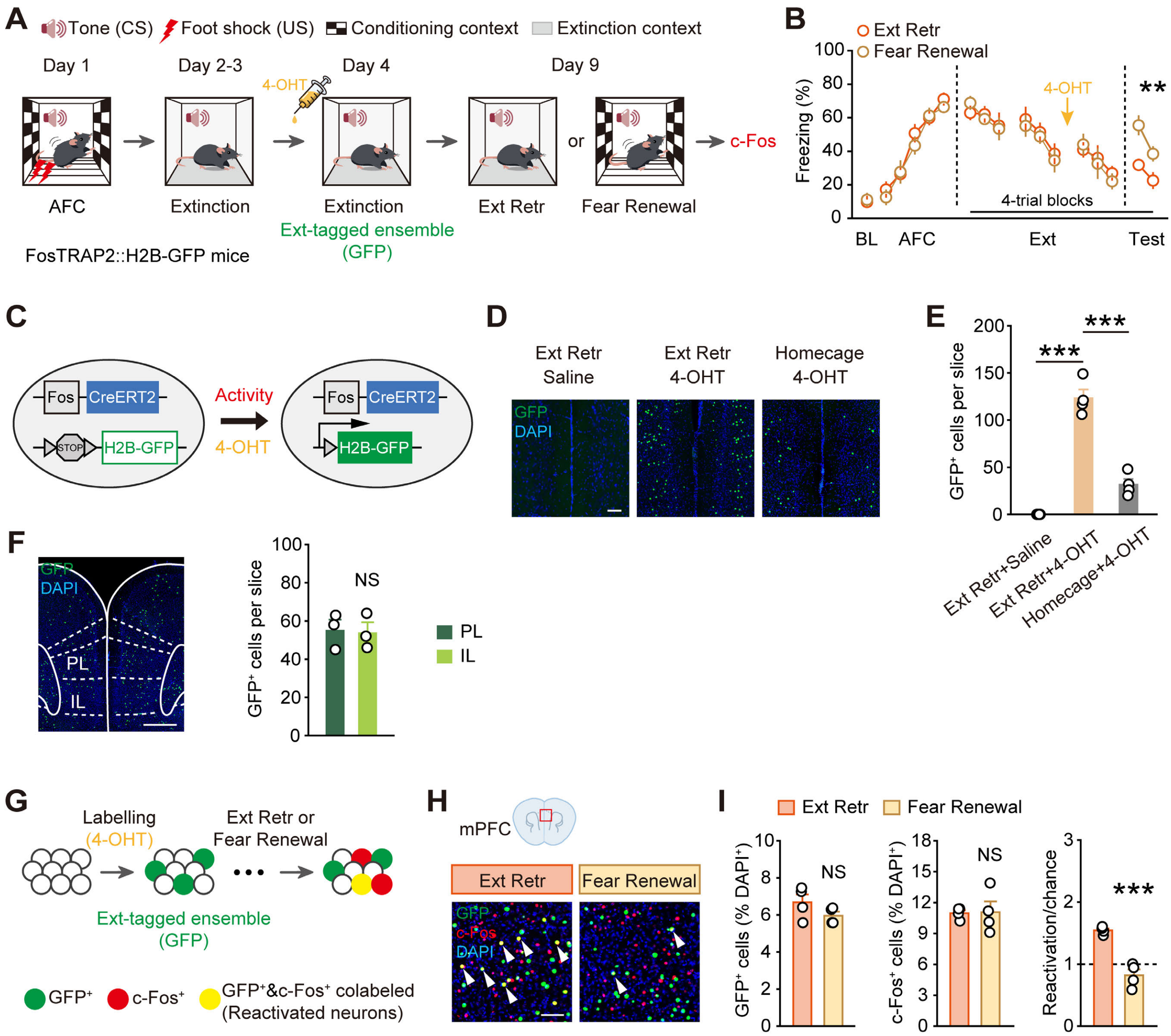

Supplemental Figure 1. Genetic capture and tagging of extinction ensembles in mPFC. (**A**) Schematics of the behavioral procedure. (**B**) Freezing responses to the context only (baseline, BL) or CS. Freezing response during AFC was calculated by percent freezing time to CS in individual trials; those during extinction training and memory test were calculated by the average freezing responses of four consecutive trials. Statistics are as follows: main effect of behavior, conditioning, $F_{1,12} = 0.3855$, $P = 0.5463$; extinction learning, $F_{1,12} = 0.3311$, $P = 0.5756$; memory test, $F_{1,12} = 15.00$, $P = 0.0022$. n = 7 mice per group. (**C**) Principles of TRAP labeling. The TRAP system uses the *Fos* gene locus to drive the expression of tamoxifen-inducible CreERT2, which enables recombination of a transgenically expressed Cre-dependent effector. In the presence of 4-OHT, CreERT2 recombination can occur in active neurons. (**D**) Representative images of EGFP expression. Scale bar, 200 μm. (**E**) Quantification of EGFP$^+$ cells. n = 4 mice per group. (**F**) Representative image of GFP expression and quantification of GFP$^+$ cells. Scale bar, 500 μm. n = 3 mice per group. (**G**) Genetic design to investigate extinction-tagged neurons and activated neurons during extinction retrieval and fear renewal. (**H**) Representative images of GFP$^+$ and c-Fos$^+$ immunofluorescence in mPFC. The white arrowheads denote colabeled GFP$^+$/c-Fos$^+$ neurons. Scale bar, 100 μm. (**I**) Quantitative statistics of TRAPed neurons, c-Fos$^+$

neurons, and reactivated neurons. The dashed line referred to the value as 1. n = 4 mice per group. Data are mean ± SEM. NS, no significant difference, $^{**}P < 0.01$, $^{***}P < 0.001$. Two-way repeated measures ANOVA (**B**), one-way ANOVA with Tukey's multiple-comparison test (**E**), and unpaired Student's *t* test (**F** and **I**).

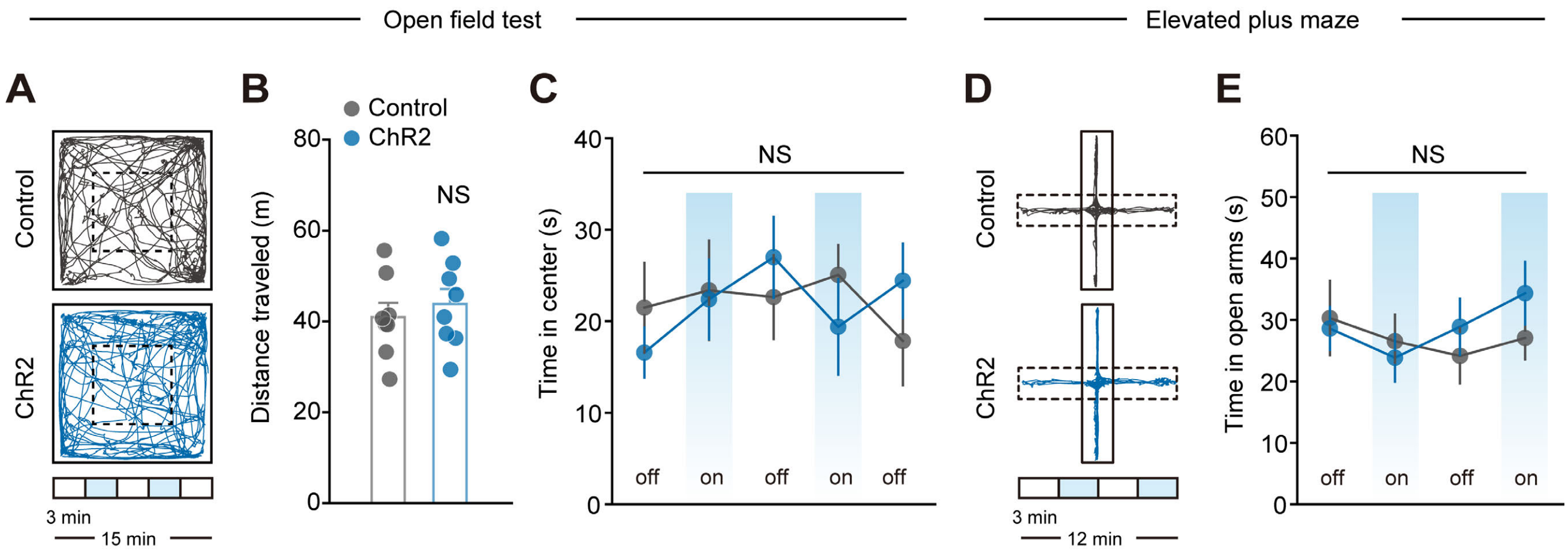

Supplemental Figure 2. Stimulation of extinction ensembles has no effect on locomotor activity. (**A**-**C**) The locomotor activity (**A**), total distances traveled (**B**), and total time in the center in a 3-min laser-off and laser-on period (**C**) for Control group and ChR2 group. n = 8 mice per group. (**D** and **E**) The locomotor activity (**D**) and time in open arms in a 3-min laser-off and laser-on period (**E**) for Control group and ChR2 group. n = 8 mice per group. Data are mean ± SEM. NS, no significant difference. Unpaired Student's *t* test (**B**) and repeated measures two-way ANOVA with Sidak's multiple-comparisons test in (**C** and **E**).

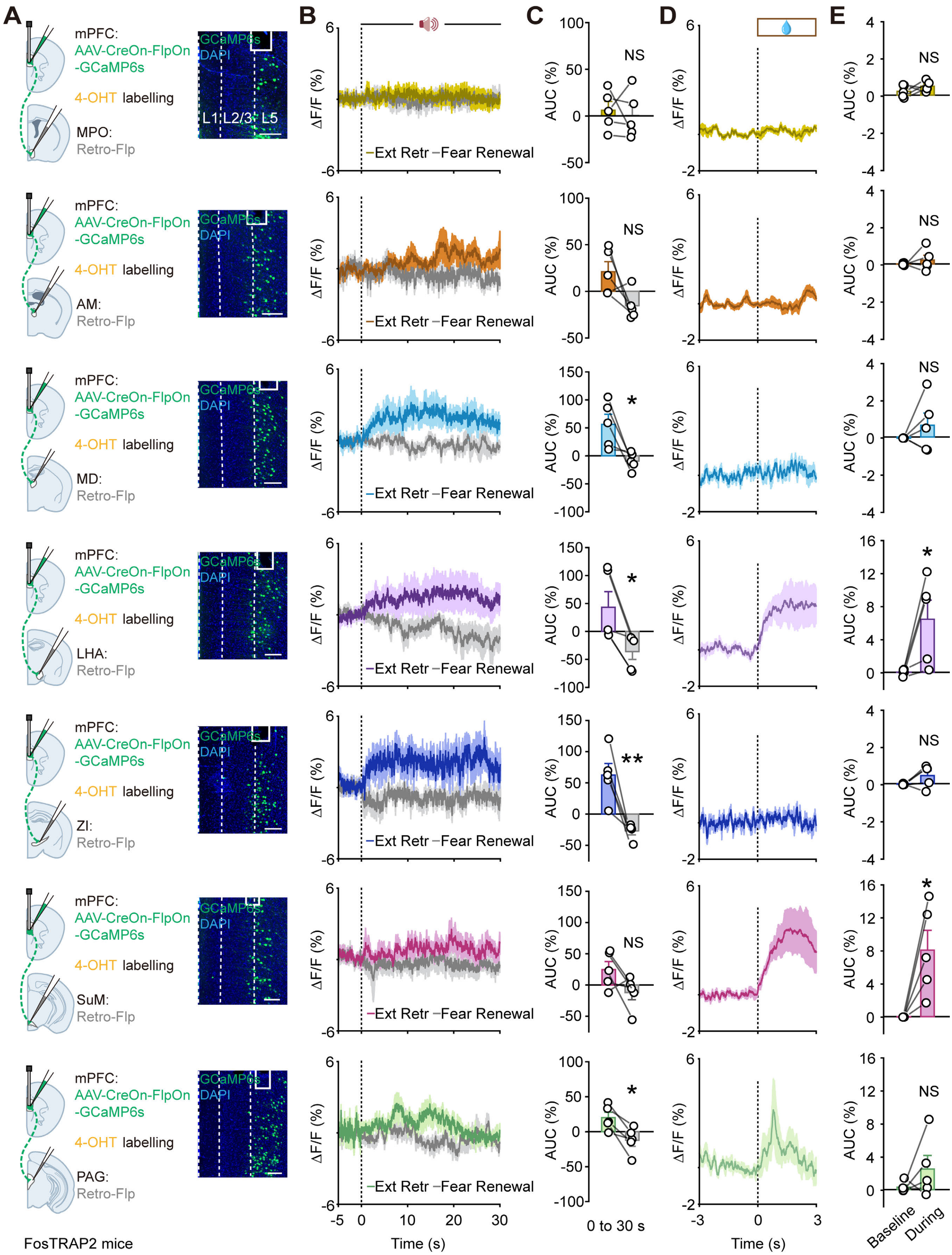

Supplemental Figure 3. Calcium signal of different downstream-projecting mPFC extinction ensembles during memory test and water intake. (**A**) Schematics of experimental design and representative images of

GCaMP6s expression in mPFC. Scale bars, 200 μm. (**B-E**) Average GCaMP signals and area under the curve (AUC) of neuronal calcium responses during extinction retrieval and fear renewal (**B** and **C**) and water intake (**D** and **E**). n = 5 mice per group. Data are mean ± SEM. NS, no significant difference, *P < 0.05, **P < 0.01. Paired Student's *t* test (**C** and **E**).

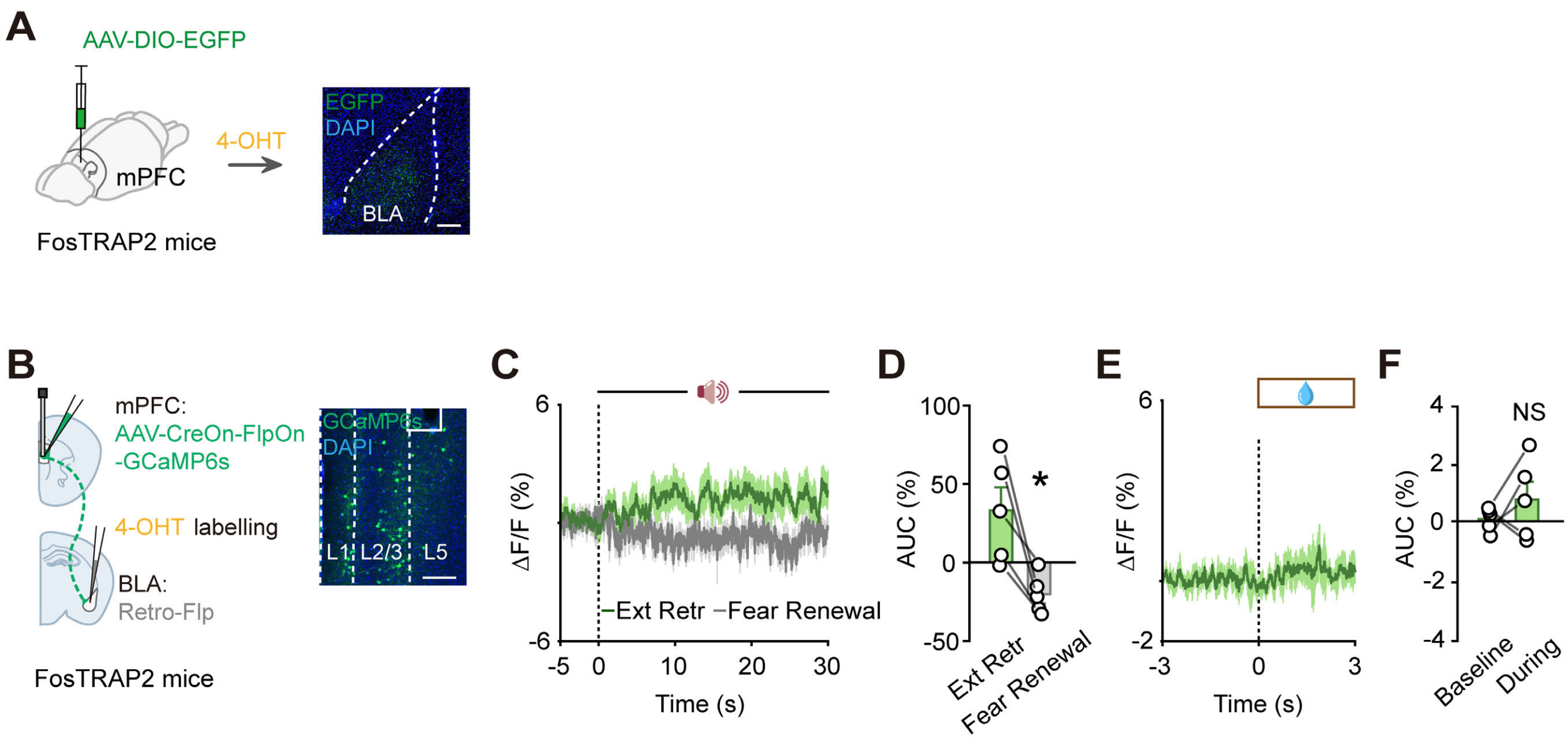

Supplemental Figure 4. Calcium signal of BLA-projecting mPFC extinction ensembles during memory test and water intake. (**A**) Projection targets of mPFC extinction ensembles in BLA. Scale bar, 200 µm. BLA, basolateral amygdala. (**B**) Schematics of experimental design and representative image of GCaMP6s expression in mPFC. Scale bar, 200 µm. (**C-F**) Average GCaMP signals and AUC of neuronal calcium responses during extinction retrieval and fear renewal (**C** and **D**) and water intake (**E** and **F**). n = 5 mice per group. Data are mean ± SEM. NS, no significant difference, *$P < 0.05$. Paired Student's t test (**D** and **F**).

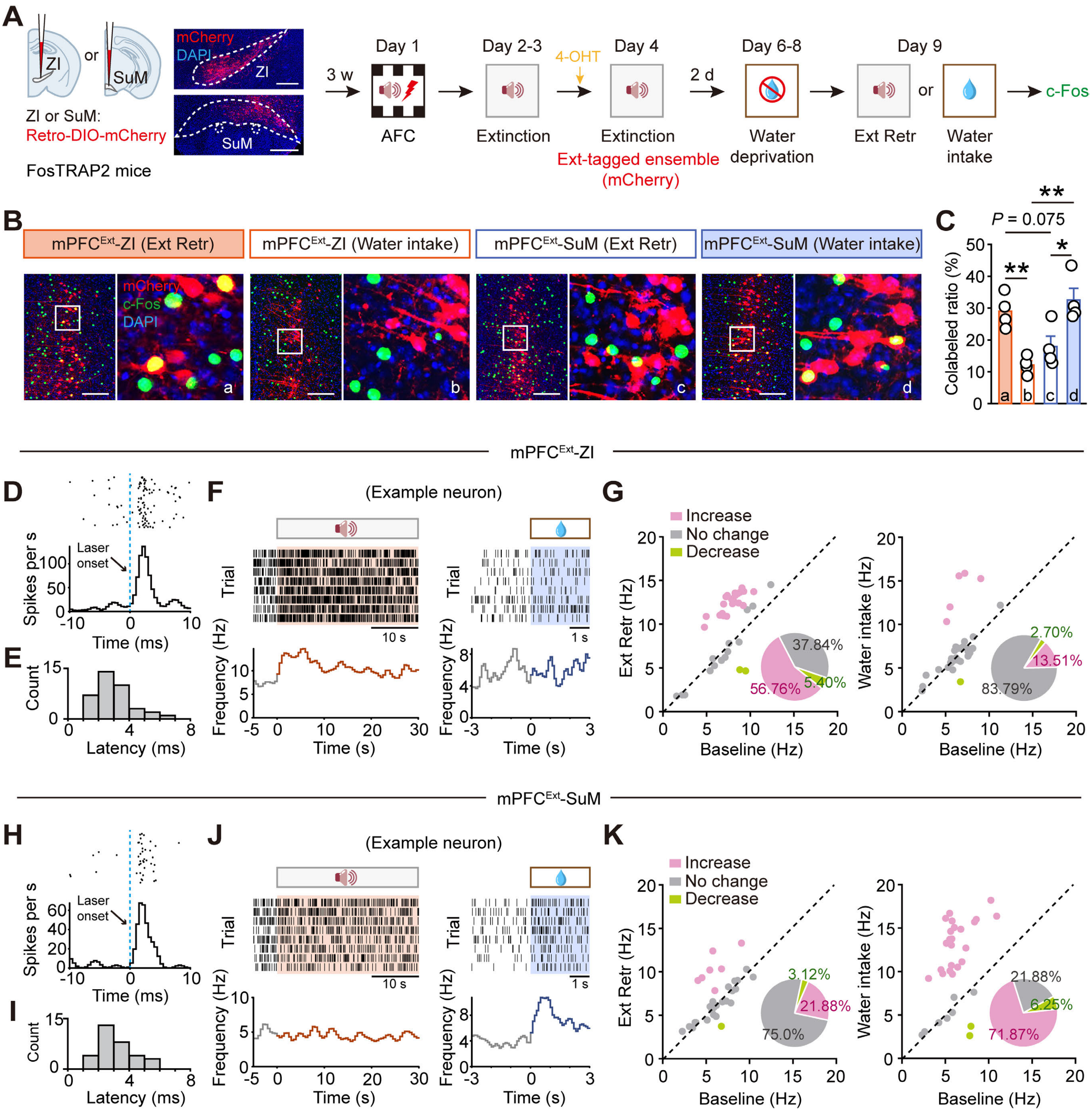

Supplemental Figure 5. Different ZI- or SuM-projecting mPFC extinction ensembles are involved in extinction retrieval and positive affective experience. (**A**) Schematics of experimental design. Representative images showing mCherry expression in ZI or SuM, scale bars, 500 μm. (**B**) Representative images of mCherry$^+$ (red) and c-Fos$^+$ (green) immunofluorescence in mPFC. Scale bars, 200 μm. (**C**) Colabeled ratio of mCherry$^+$ and c-Fos$^+$ immunofluorescence. n = 4 mice per group. (**D** and **H**) Sample of peri-event raster plots and PSTH for light-evoked spike latency of ChR2-tagged ZI-projecting (**D**) or SuM-projecting (**H**) mPFC extinction neuron. (**E** and **I**) Plot showing short latencies of light-evoked spike for all ChR2-tagged ZI-projecting (**E**) or SuM-projecting (**I**) mPFC extinction ensembles. (**F** and **J**) Raster plot (top) and PSTH (bottom) for spike responses of an example ChR2-tagged ZI-projecting (**F**) or SuM-projecting (**J**) mPFC extinction neuron during Ext Retr (left) and Water intake (right). The orange or blue box marks the duration of stimulation. (**G** and **K**) Scatter plots of the firing rates of all ChR2-tagged ZI-projecting (**G**) or SuM-projecting (**K**) mPFC extinction ensembles during Ext Retr and Water intake. Pie charts summarize the fractions of the populations. Data are

mean ± SEM. *$P < 0.05$, **$P < 0.01$. One-way ANOVA with Tukey's multiple-comparison test (**C**).

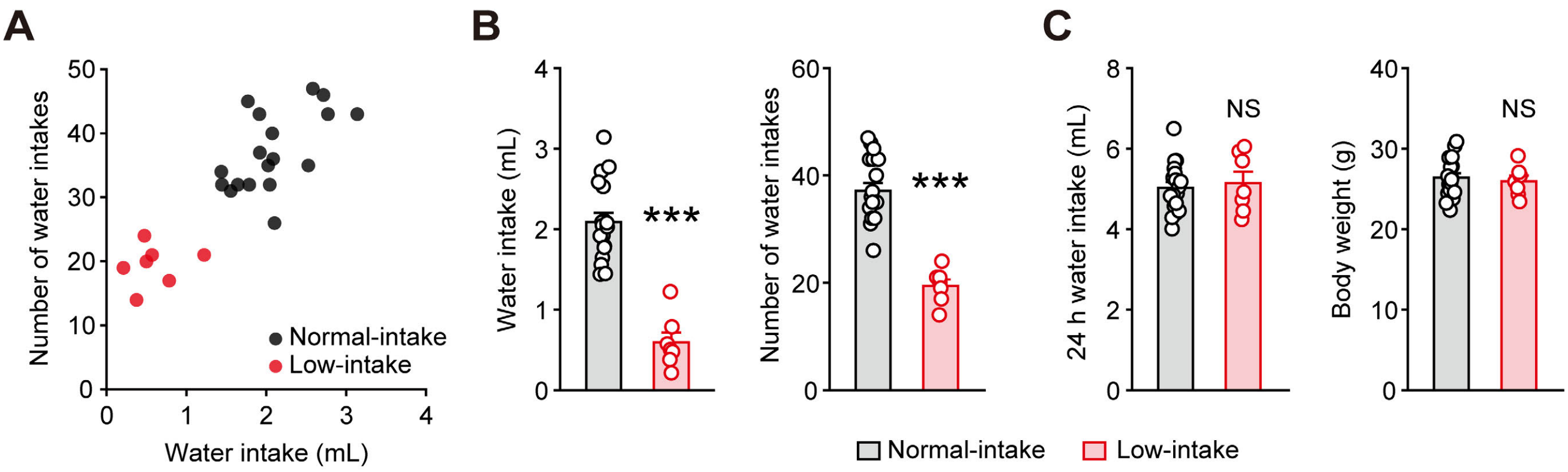

Supplemental Figure 6. Baseline parameters of normal-intake mice and low-intake mice. (**A**) Unsupervised *K*-means clustering of water consumption behavior reveals two distinct subgroups of mice. Normal-intake group, n = 18 mice; Low-intake group, n = 7 mice. (**B**) Plots showing water intake and numbers of water intake between normal-intake mice and low-intake mice. (**C**) The baseline level of 24-hour water intake and body weight of normal-intake mice and low-intake mice. Data are mean ± SEM. NS, no significant difference, ****P* < 0.001. Unpaired Student's *t* test (**B** and **C**).

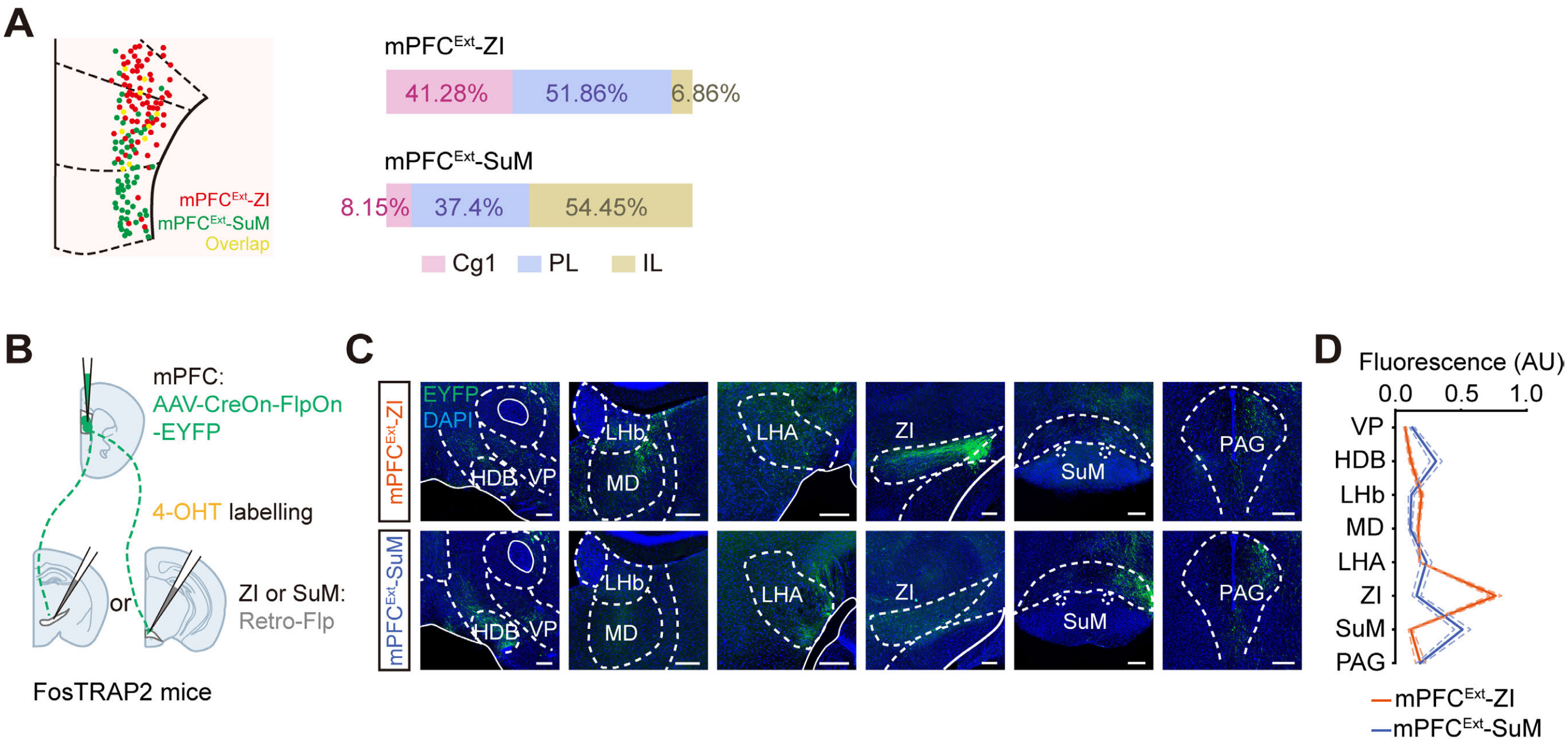

Supplemental Figure 7. Distribution of ZI- or SuM-projecting mPFC extinction ensembles and downstream fluorescence of mPFC projector. (**A**) Distribution of ZI- or SuM-projecting mPFC extinction ensembles in subregions of mPFC. Cg1, cingulate cortex, area 1; PL, prelimbic cortex; IL, infralimbic cortex. (**B**) Schematics of experimental design. (**C**) Representative images of downstream fluorescence of ZI- or SuM-projecting mPFC extinction ensembles. Scale bars, 200 μm. (**D**) Quantification of fluorescence in the downstream brain regions originating from mPFC^{Ext}-ZI or mPFC^{Ext}-SuM neurons. Data are mean ± SEM.

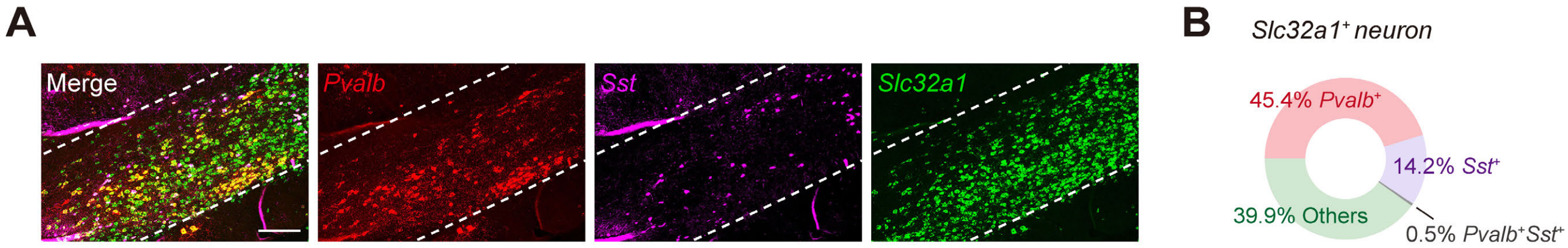

Supplemental Figure 8. Expression of *Pvalb* and *Sst* in *Slc32a1*$^+$ GABAergic neurons. (**A**) Triple-color RNAscope in ZI showing *Pvalb* mRNA (red), *Sst* mRNA (purple), and *Slc32a1* (vesicular GABA transporter, vGAT; green). Scale bar, 200 μm. **(B)** Quantification of proportions of *Slc32a1*$^+$ *neurons* co-expressing *Pvalb* and *Sst*.

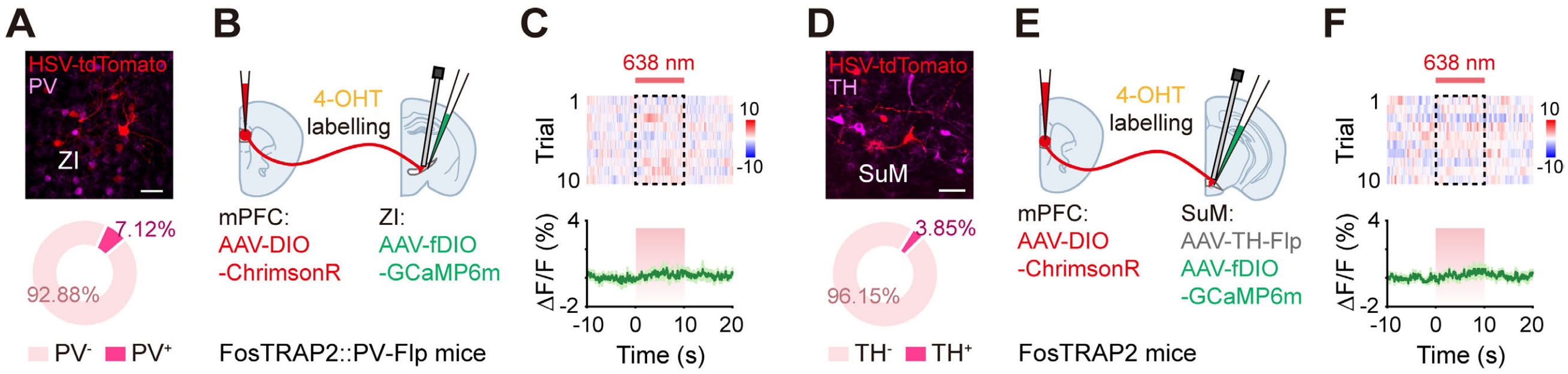

Supplemental Figure 9. Validation of neuronal types. (**A** and **D**) Representative images and colocalization quantification of HSV-tdTomato and PV immunofluorescence in ZI (**A**) or HSV-tdTomato and TH immunofluorescence in SuM (**D**). Scale bars, 50 μm. (**B** and **E**) Schematic of viral injection for fiber photometry. (**C** and **F**) Representative heatmaps and averaged fluorescence in response to optogenetic stimulation in ZI PV neurons (**C**) or SuM dopaminergic neurons (**F**). Data are mean ± SEM.

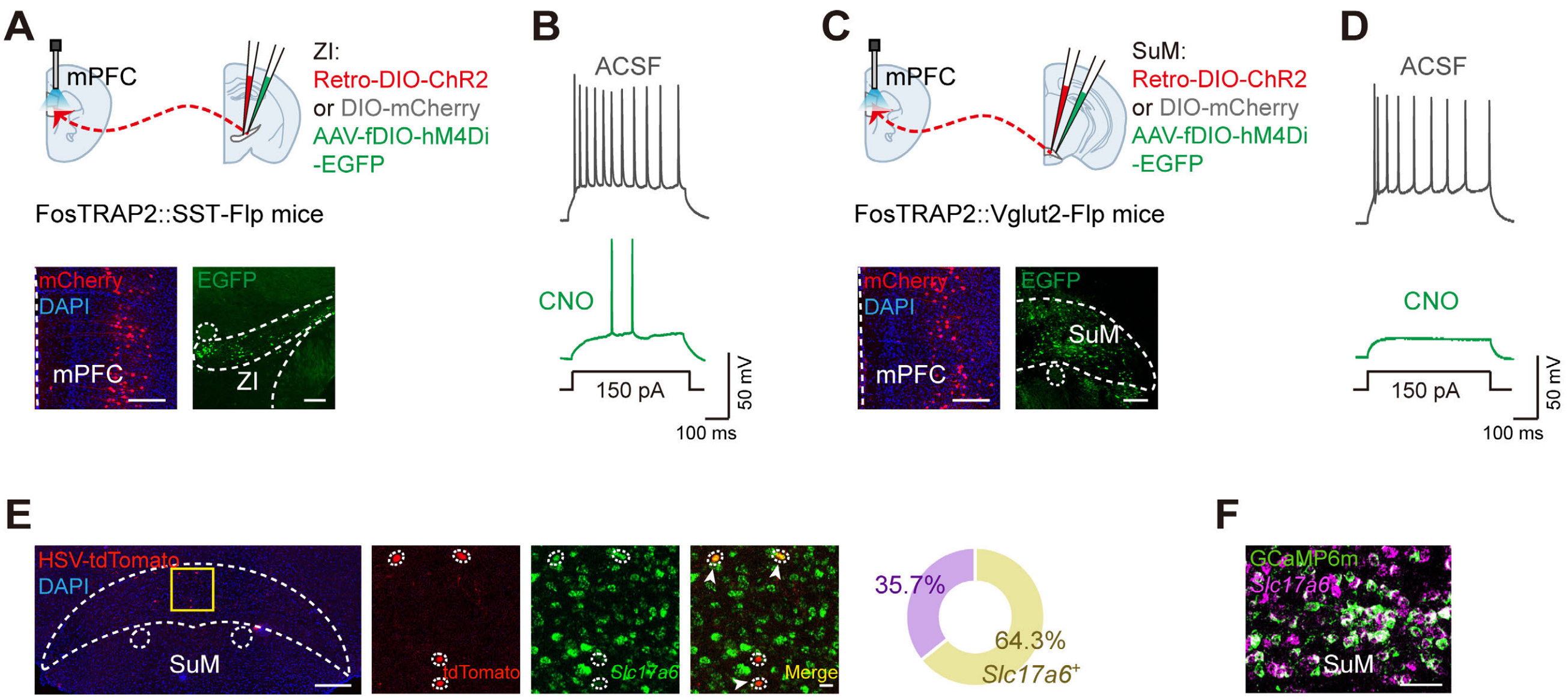

Supplemental Figure 10. Functional validation and histological verification. (**A** and **C**) Schematics of experimental design and representative images of mCherry and EGFP expression. Scale bars, 200 µm. (**B** and **D**) Functional validation for chemogenetic inhibition of ZI^{SST} neurons or SuM^{Glu} neurons. (**E**) HSV-tdTomato expression and RNAscope in SuM showing *Slc17a6* (vesicular glutamate transporter 2, VGLUT2). Scale bars, 200 µm (low-magnification) and 20 µm (high-magnification). Quantification of colocalization of HSV-tdTomato and *Slc17a6*. (**F**) Representative images of GCaMP6m expression and *Slc17a6* in SuM. Scale bar, 50 µm.

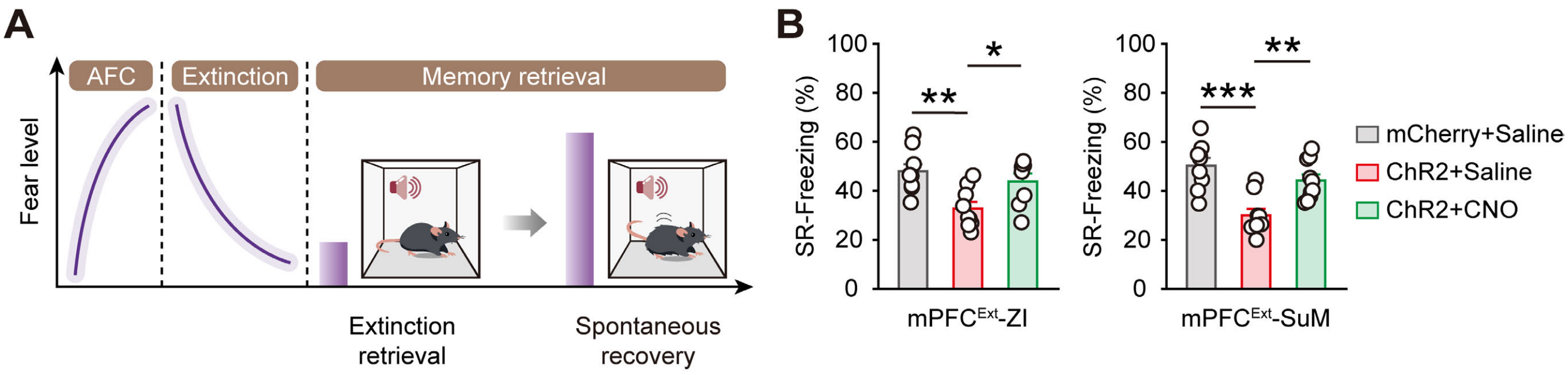

Supplemental Figure 11. Manipulation of mPFC^{Ext}-ZI^{SST} or mPFC^{Ext}-SuM^{Glu} pathways on spontaneous recovery. (**A**) Schematics of spontaneous recovery. Compared to the extinction retrieval, the extinguished fear levels relapsed after a relatively long-term period following fear extinction. (**B**) Effects of manipulating mPFC^{Ext}-ZI^{SST} or mPFC^{Ext}-SuM^{Glu} pathways on SR test. mPFC^{Ext}-ZI^{SST}: mCherry+Saline group, n = 9 mice; ChR2+Saline group, n = 9 mice; ChR2+CNO group = 8 mice. mPFC^{Ext}-SuM^{Glu}: mCherry+Saline group, n = 9 mice; ChR2+Saline group, n = 9 mice; ChR2+CNO group = 10 mice. The data shown in this panel were obtained from the same experiments illustrated in Figure 5J and Figure 6J. Laser stimulation was delivered during the spontaneous recovery test. Data are mean ± SEM. *$P < 0.05$, **$P < 0.01$, ***$P < 0.001$. one-way ANOVA with Bonferroni's multiple-comparison test (**B**)

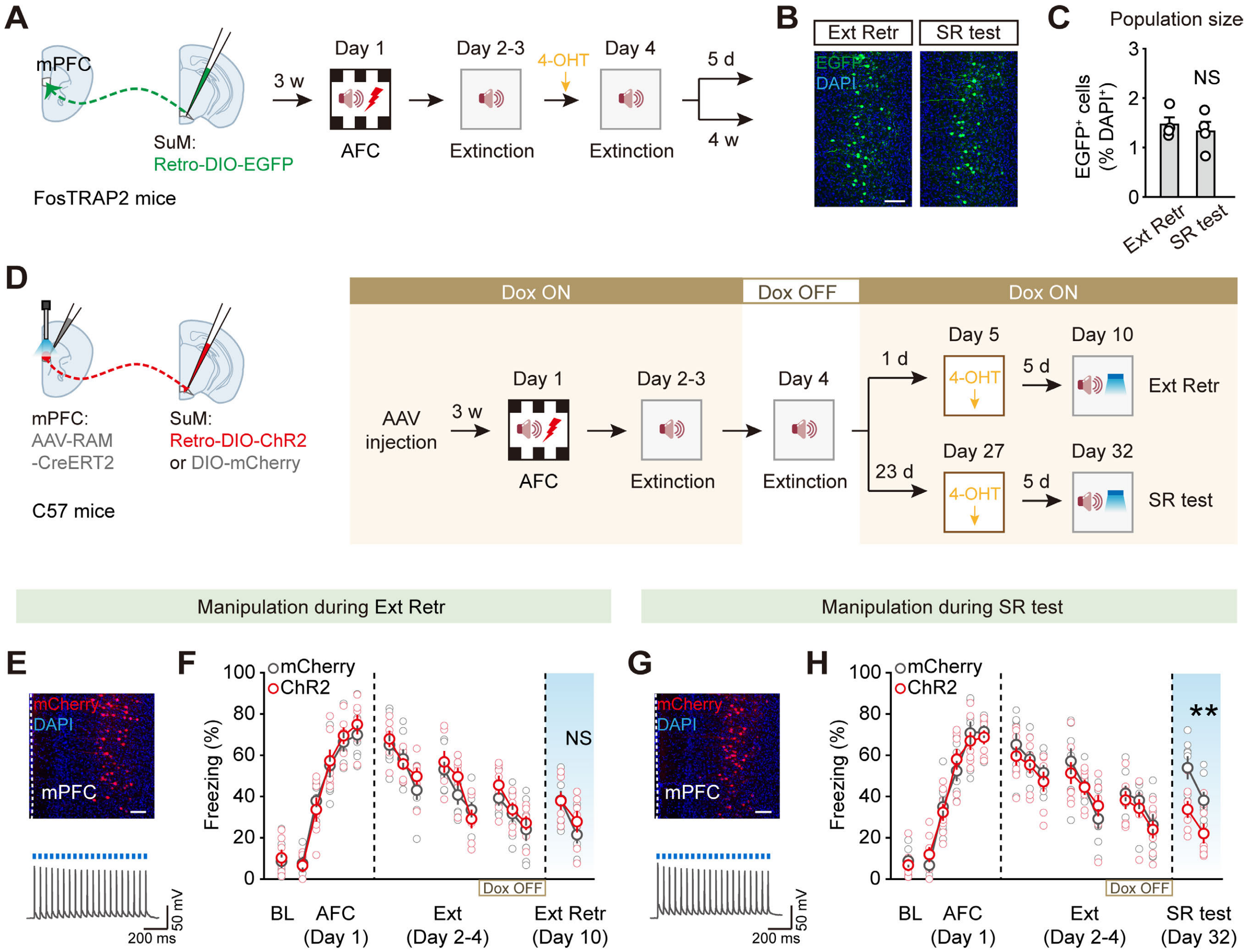

Supplemental Figure 12. Neuronal labeling and manipulation during extinction retrieval and spontaneous recovery. (**A**) Schematics of AAV injections and schematics of experimental design. (**B**) Representative image of virus expression. Scale bar, 100 μm. (**C**) The Ext Retr group and SR group displayed similar percentages of tagged neurons. n = 4 mice per group. (**D**) Schematics of experimental design. AAVs expressing Dox-dependent CreERT2 and Cre-dependent ChR2 or mCherry were microinjected in mPFC and SuM. The mice were kept on Dox for the duration of the experiment except for extinction training on Day 4 to tag the neurons with CreERT2. Five days before memory retrieval, mice were injected with 4-OHT to induce ChR2 expression in activated neurons. (**E** and **G**) Representative images of mCherry expression in mPFC and functional validation for optogenetic activation of mPFC neurons. Scale bars, 100 μm. (**F**) Effects of stimulating SuM-projecting mPFC^{Ext} neurons on extinction retrieval. Time courses of freezing responses to the CS. Statistics are as follows: main effect of AAV, conditioning, $F_{1,13} = 0.1118$, $P = 0.7434$; extinction training, $F_{1,13} = 0.8769$, $P = 0.3661$; extinction retrieval, $F_{1,13} = 0.5196$, $P = 0.4838$. mCherry group, n = 8 mice, ChR2 group, n = 7 mice. (**H**) Effects of stimulating SuM-projecting mPFC^{Ext} neurons on spontaneous recovery. Time courses of freezing responses to the CS. Statistics are as follows: main effect of AAV, conditioning, $F_{1,14} = 0.01289$, $P = 0.9112$; extinction training, $F_{1,14} = 0.9283$, $P = 0.3516$; spontaneous recovery, $F_{1,14} = 11.82$, $P = 0.0040$. n = 8 mice per group. Data are mean ± SEM. NS, no significant difference, $^{**}P < 0.01$. Unpaired Student's t test (**C**) and two-way repeated measures ANOVA (**F** and **H**).

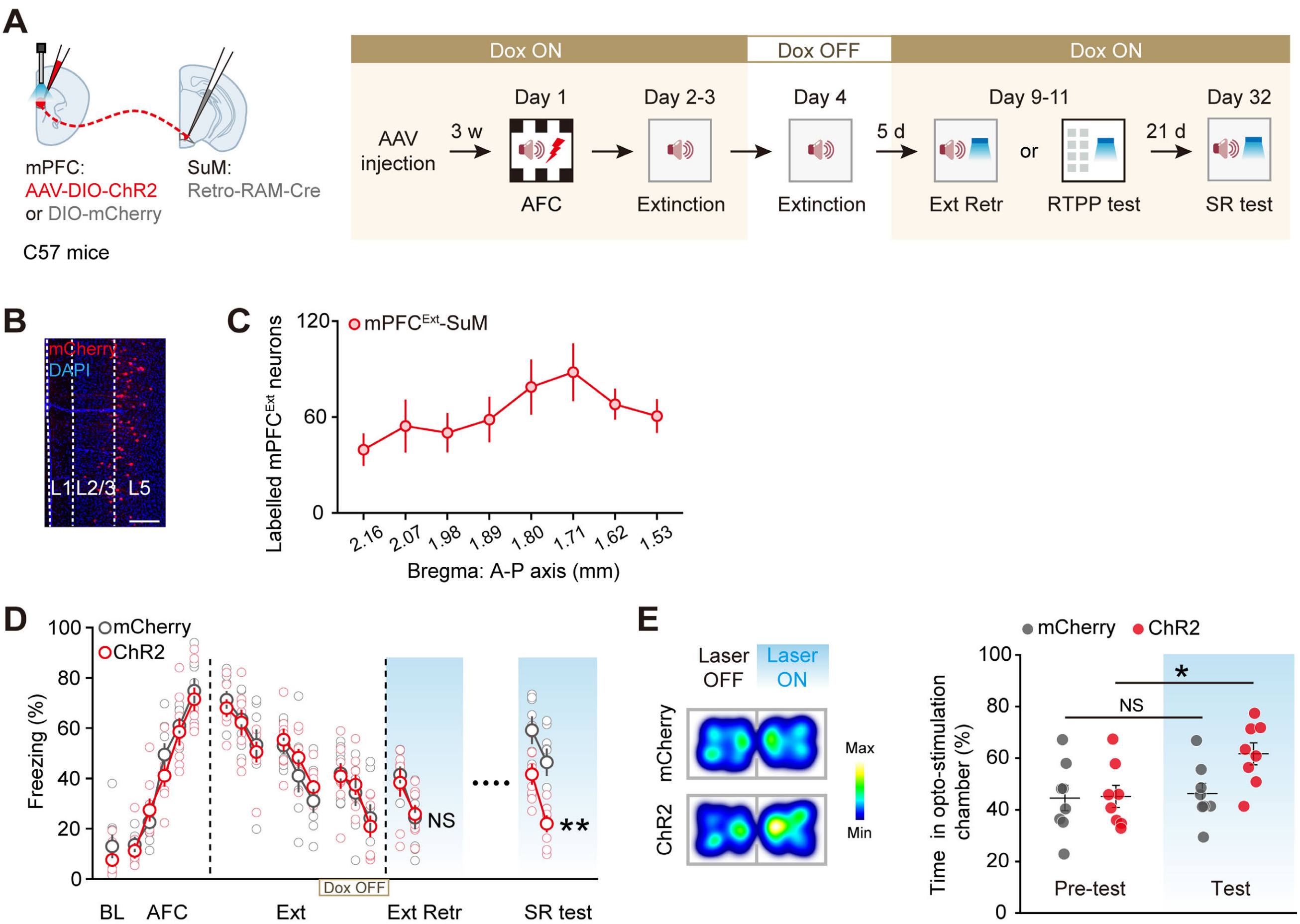

Supplemental Figure 13. Verification of the RAM system. (**A**) Schematics of AAV injections and schematics of experimental design. (**B**) Representative image of mCherry expression. Scale bar, 500 µm. (**C**) Number and distribution of SuM-projecting mPFC extinction ensembles marked by the RAM system. (**D**) Effects of activating mPFC^{Ext}–SuM projectors on extinction retrieval and spontaneous recovery. Statistics are as follows: conditioning, $F_{1,14} = 0.3242$, $P = 0.5781$; extinction training, $F_{1,14} = 0.04520$, $P = 0.8347$; extinction retrieval, $F_{1,14} = 0.03128$, $P = 0.8621$; spontaneous recovery, $F_{1,14} = 13.17$, $P = 0.0027$; n = 8 mice per group. (**E**) Effects of activating mPFC^{Ext}–SuM projectors on real-time place preference. Heatmaps of the locomotor activity and the percentage of time in opto-stimulation chamber. n = 8 mice per group. Data are mean ± SEM. NS, no significant difference, *$P < 0.05$, **$P < 0.01$. Two-way repeated measures ANOVA (**D**) and paired Student's *t*-test (**E**).

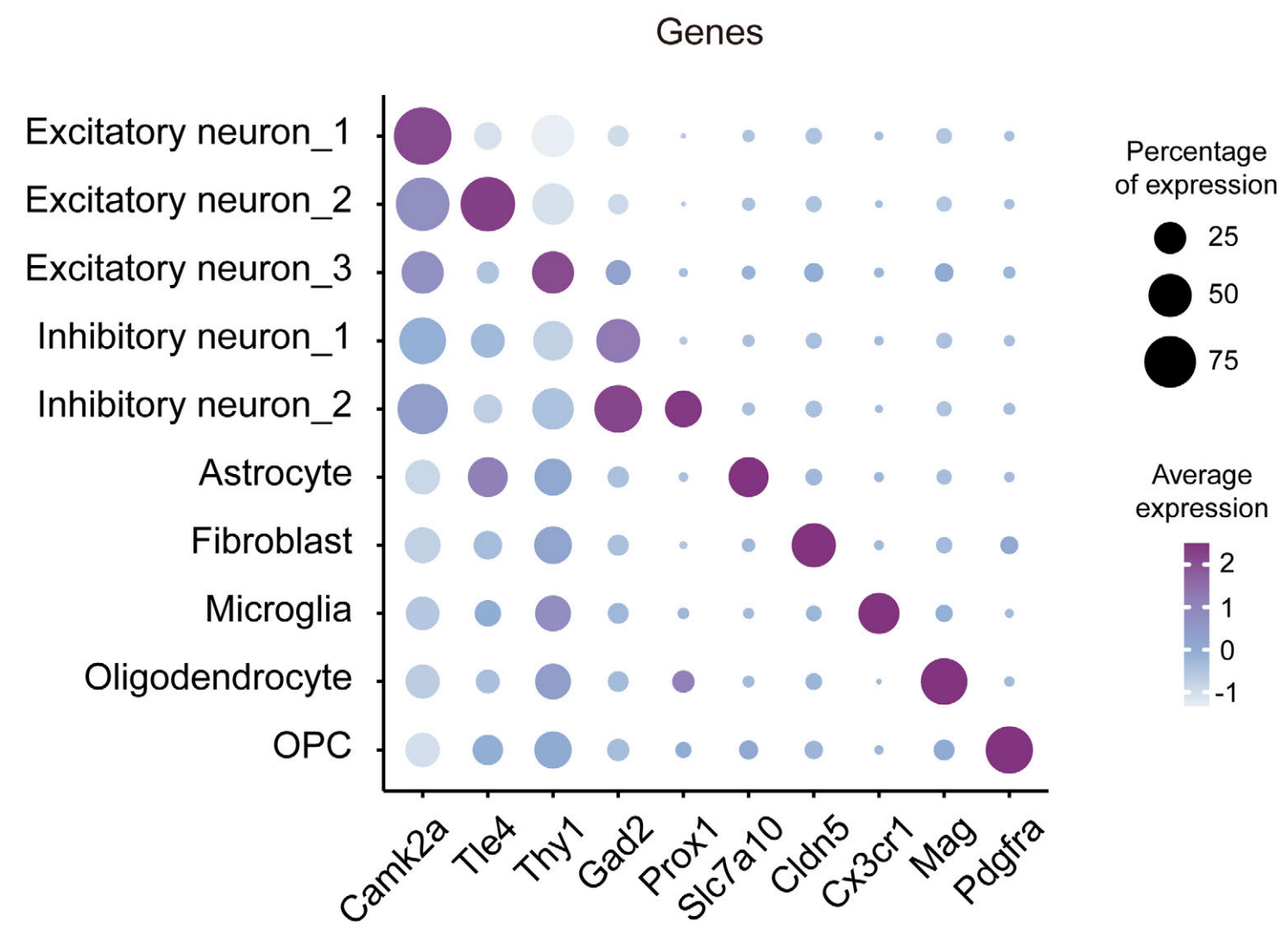

Supplemental Figure 14. Quality control and validation of snRNA-seq. Dot plot showing the expression patterns of marker genes across 10 cell types.

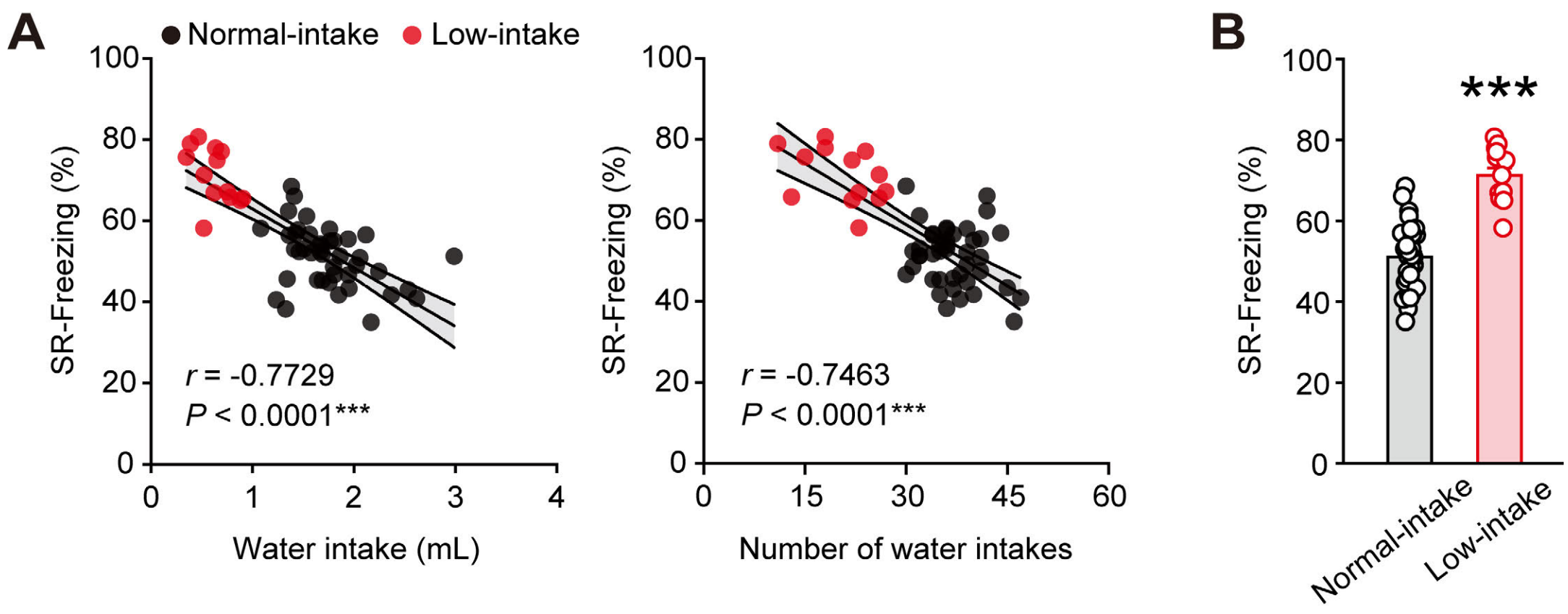

Supplemental Figure 15. Low-intake mice display increased freezing during spontaneous recovery. (**A**) Plots showing the correlation of water intake and freezing level during spontaneous recovery (SR). Circles represent individual mice, solid lines denote the linear regressions, and shaded regions show the 95% confidence intervals. (**B**) Plots showing SR freezing level between normal-intake mice and low-intake mice. Data are mean ± SEM. ***$P < 0.001$. Linear regression (**A**) and unpaired Student's *t* test (**B**).

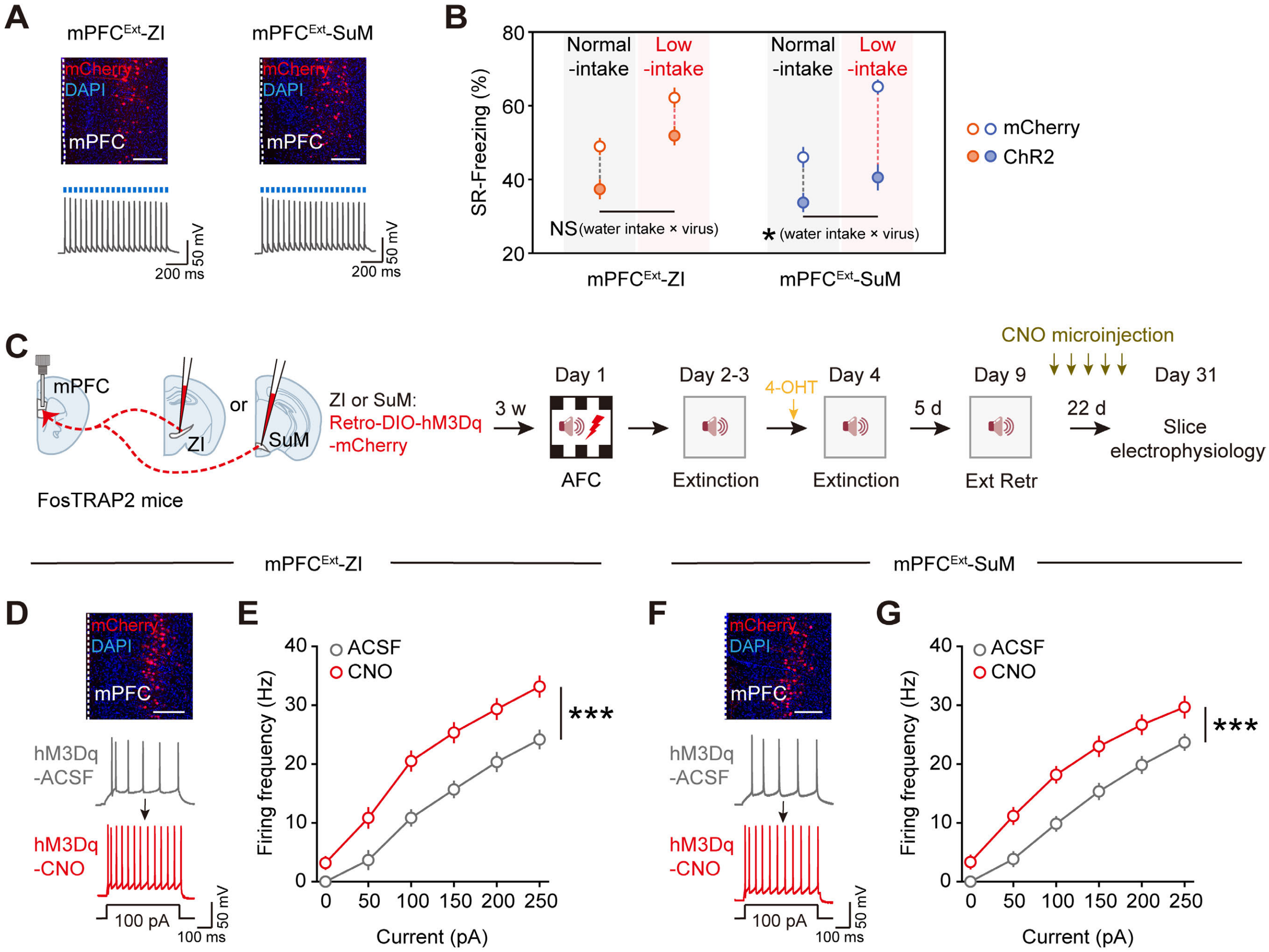

Supplemental Figure 16. Functional validation for optogenetic and chemogenetic activation of mPFC neurons. (**A**) Representative images of mCherry expression in mPFC and functional validation for optogenetic activation of mPFC neurons. Scale bars, 200 µm. (**B**) Effects of activating mPFC^{Ext}–ZI or mPFC^{Ext}–SuM projectors on SR test in normal-intake mice and low-intake mice. Statistics are as follows: mPFC^{Ext}–ZI: water intake × virus $F_{1,42} = 0.05855$, $P = 0.8100$. mPFC^{Ext}–SuM: water intake × virus $F_{1,42} = 4.445$, $P = 0.0410$. (**C**) Schematics of AAV injections and schematics of experimental design. (**D** and **F**) Representative images of mCherry expression in mPFC and functional validation for chemogenetic activation of mPFC neurons. Scale bars, 200 µm. (**E** and **G**) AP frequencies of mPFC tagged neurons. n = 12 cells per group. Data are mean ± SEM. NS, no significant difference, *$P < 0.05$, ***$P < 0.001$. Two-way ANOVA (**B**) and two-way repeated measures ANOVA (**E** and **G**).

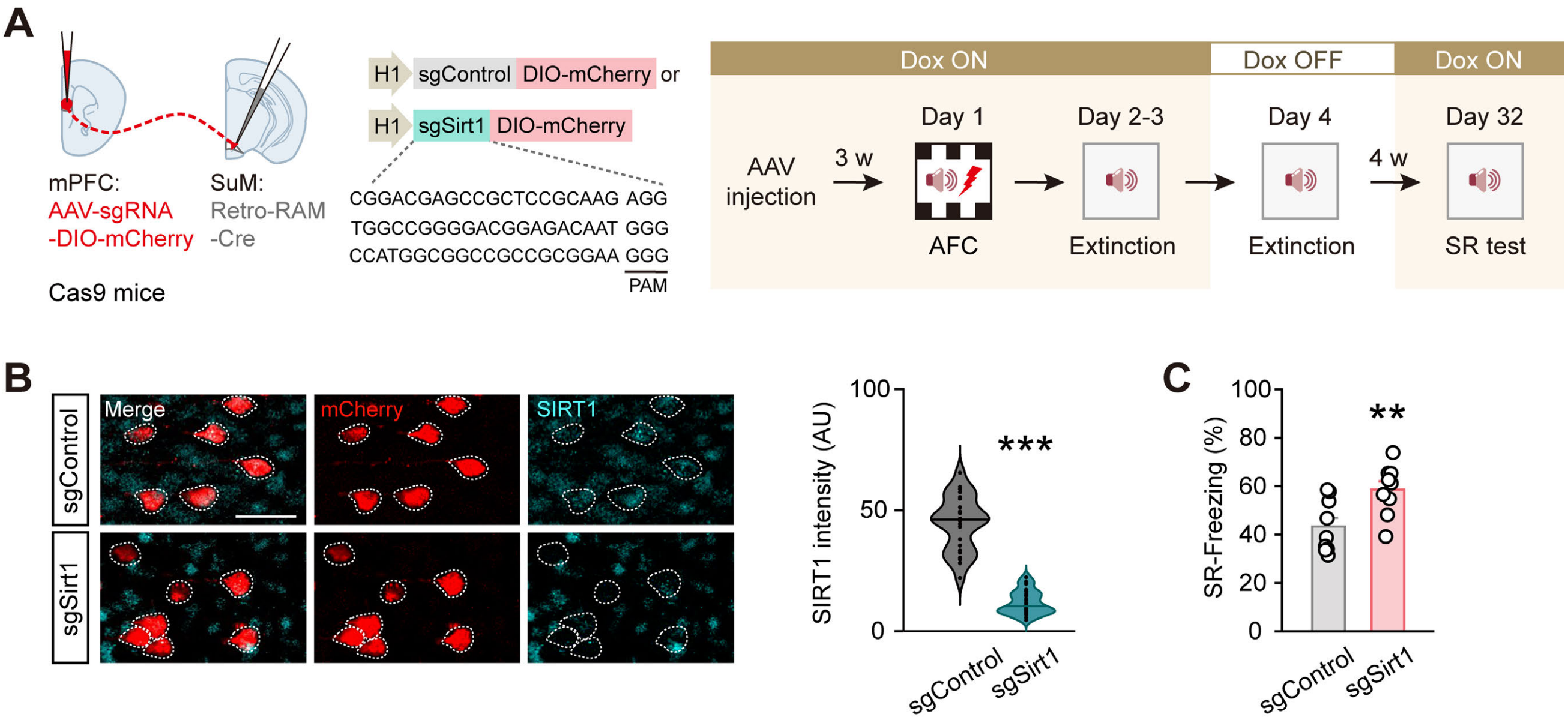

Supplemental Figure 17. The effect of *Sirt1* knockout on spontaneous recovery in non-stressed mice. (**A**) Loss-of-function experiment and schematics of AAV bilateral injection. The viral construct used for CRISPR-Cas9-mediated conditional knockout of *Sirt1* in the SuM-projecting mPFC^{Ext} neurons. (**B**) Representative images and quantification of SIRT1 intensity in the SuM-projecting mPFC^{Ext} neurons. Scale bar, 50 μm. n = 24 sections from 6 mice per group. (**C**) Effects of CRISPR-Cas9-mediated conditional knockout of *Sirt1* in the SuM-projecting mPFC^{Ext} neurons on SR test. sgControl group, n = 9 mice; sgSirt1 group, n = 9 mice. Data are mean ± SEM. $^{**}P < 0.01$, $^{***}P < 0.001$. Unpaired Student's *t* test (**B** and **C**).

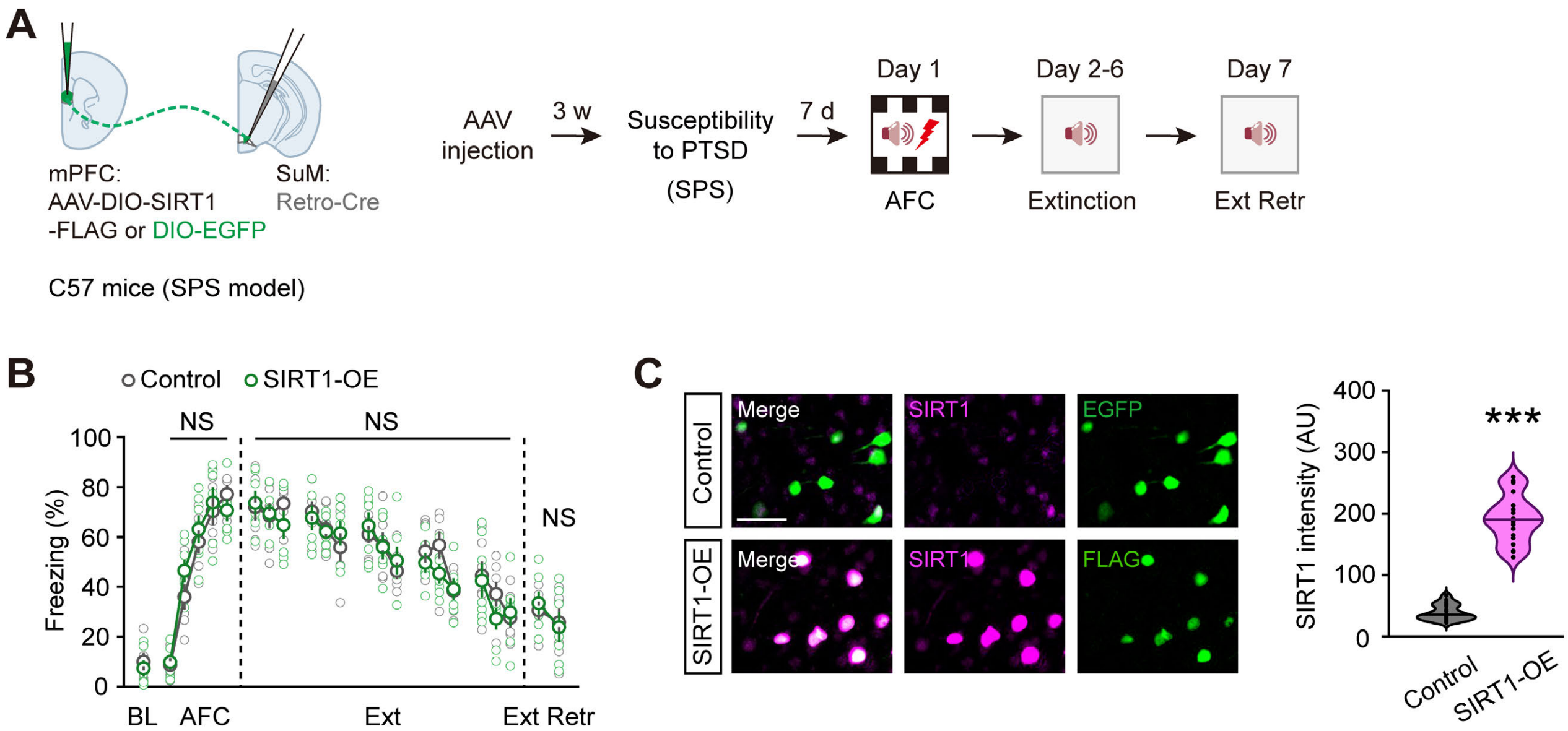

Supplemental Figure 18. Effects of SIRT1 overexpression in the SuM-projecting mPFC neurons on fear conditioning, extinction and extinction retrieval. (**A**) Gain-of-function experiment and schematics of AAV injection. (**B**) Time courses of freezing responses to the CS. Statistics are as follows: main effect of treatment, conditioning, $F_{1,12} = 1.307$, $P = 0.2752$; extinction training, $F_{1,12} = 0.8565$, $P = 0.3729$; extinction retrieval, $F_{1,12} = 0.007229$, $P = 0.9336$. n = 7 mice per group. (**C**) Representative images and quantification of SIRT1 overexpression efficiency in the SuM-projecting mPFC neurons. Scale bar, 50 µm. n = 20 sections from 5 mice per group. Data are mean ± SEM. NS, no significant difference, ***P < 0.001. Two-way repeated measures ANOVA (**B**) and unpaired Student's t test (**C**).

Supplemental Table 1. Detailed statistical information

Figure	Sample	Number (Female/Male)	Statistic method	Statistic results
Figure 1C	mCherry, NpHR	n = 7 (0/7),<br>n = 7 (0/7)	Two-way repeated measures ANOVA	$F_{1,12}$ = 6.502, P = 0.0255
Figure 1D	mCherry, NpHR	n = 7 (0/7),<br>n = 7 (0/7)	Unpaired Student's *t* test	t = 3.290, df = 12, P = 0.0065
Figure 1E	mCherry, NpHR	n = 7 (0/7),<br>n = 7 (0/7)	Unpaired Student's *t* test	t = 4.234, df = 12, P = 0.0012
Figure 1F	mCherry, ChR2	n = 8 (0/8),<br>n = 9 (0/9)	Two-way repeated measures ANOVA	$F_{1,15}$ = 8.857, P = 0.0094
Figure 1G	mCherry, ChR2	n = 8 (0/8),<br>n = 9 (0/9)	Paired Student's *t*-test; Two-way repeated measures ANOVA	Pre-test vs Test (mCherry): t = 0.3260, df = 7, P = 0.7540; Pre-test vs Test (ChR2): t = 4.481, df = 8, P = 0.0021; Pre-test vs Test: $F_{1,15}$ = 6.271, P = 0.0243
Figure 3H	mPFC^{Ext}-ZI, mPFC^{Ext}-SuM	n = 10 (decoding iteration),<br>n = 10 (decoding iteration)	Unpaired Student's *t* test	t = 5.742, df = 18, P < 0.0001
Figure 3I	mPFC^{Ext}-ZI, mPFC^{Ext}-SuM	n = 10 (decoding iteration),<br>n = 10 (decoding iteration)	Unpaired Student's *t* test	t = 18.33, df = 18, P < 0.0001
Figure 3J	mPFC^{Ext}-ZI, mPFC^{Ext}-SuM	n = 10 (decoding iteration),<br>n = 10 (decoding iteration)	Unpaired Student's *t* test	t = 5.622, df = 18, P < 0.0001
Figure 3K	mPFC^{Ext}-ZI, mPFC^{Ext}-SuM	n = 10 (decoding iteration),<br>n = 10 (decoding iteration)	Unpaired Student's *t* test	t = 3.016, df = 18, P = 0.0074
Figure 4F	mPFC^{Ext}-ZI, mPFC^{Ext}-SuM	n = 11 (neuron),<br>n = 9 (neuron)	Two-way repeated measures ANOVA	$F_{1,18}$ = 1.032, P = 0.3232
Figure 4G	mPFC^{Ext}-ZI, mPFC^{Ext}-SuM	n = 11 (neuron),<br>n = 9 (neuron)	Unpaired Student's *t* test	t = 3.376, df = 18, P = 0.0034
Figure 4H	mPFC^{Ext}-ZI, mPFC^{Ext}-SuM	n = 11 (neuron),<br>n = 9 (neuron)	Unpaired Student's *t* test	t = 2.332, df = 18, P = 0.0315
Figure 4L	mPFC^{Ext}-ZI, mPFC^{Ext}-SuM	n = 5 (0/5),<br>n = 5 (0/5)	Unpaired Student's *t* test	Ipsilateral:<br>ORB: t = 4.372, df = 8, P = 0.0024; Pir: t = 2.810, df = 8, P = 0.028; CM: t =

				6.464, df = 8, P = 0.0002; BLA: t = 2.683, df = 8, P = 0.0278; Contralateral: Pir: t = 2.372, df = 8, P = 0.0451;
Figure 5G	mCherry, NpHR	n = 9 (3/6), n = 9 (3/6)	Mixed-degin ANOVA	virus $F_{1,14}$ = 4.679, P = 0.048; sex $F_{1,14}$ = 0.003, P = 0.96; virus × sex $F_{1,14}$ = 0.277, P = 0.607;
Figure 5H	mCherry, NpHR	n = 9 (3/6), n = 9 (3/6)	Two-way ANOVA	virus $F_{1,14}$ = 0.7591, P = 0.3983; sex $F_{1,14}$ = 0.01289, P = 0.9112; virus × sex $F_{1,27}$ = 0.1337, P = 0.7201
Figure 5J	mCherry+Saline, ChR2+Saline, ChR2+CNO	n = 9 (0/9), n = 9 (0/9), n = 8 (0/8)	Two-way repeated measures ANOVA	mCherry+Saline vs. ChR2+Saline: extinction retrieval $F_{1,16}$ = 9.046, P = 0.0083; spontaneous recovery $F_{1,16}$ = 14.46, P = 0.0016;<br><br>ChR2+Saline vs. ChR2+CNO: extinction retrieval $F_{1,15}$ = 7.300, P = 0.0164, spontaneous recovery $F_{1,15}$ = 6.794, P = 0.0198
Figure 5L	mCherry+Saline, ChR2+Saline, ChR2+CNO	n = 9 (0/9), n = 9 (0/9), n = 8 (0/8)	Two-way repeated measures ANOVA	$F_{1,23}$ = 0.3204, P = 0.5768
Figure 6G	mCherry, NpHR	n = 8 (0/8), n = 9 (0/9)	Two-way repeated measures ANOVA	$F_{1,15}$ = 0.0359, P = 0.8523
Figure 6H	mCherry, NpHR	n = 8 (0/8), n = 9 (0/9)	Unpaired Student's *t* test	t = 2.587, df = 15, P = 0.0206
Figure 6J	mCherry+Saline, ChR2+Saline, ChR2+CNO	n = 9 (0/9), n = 9 (0/9), n = 10 (0/10)	Two-way repeated measures ANOVA	mCherry+Saline vs. ChR2+Saline: extinction retrieval $F_{1,16}$ = 0.5645, P = 0.4634; spontaneous recovery $F_{1,16}$ = 24.33, P = 0.0002;<br><br>ChR2+Saline vs. ChR2+CNO: extinction retrieval $F_{1,17}$ = 0.0081, P

				= 0.9294, spontaneous recovery $F_{1,17}$ = 14.92, P = 0.0012
Figure 6L	mCherry+Saline,<br>ChR2+Saline,<br>ChR2+CNO	n = 9 (0/9),<br>n = 9 (0/9),<br>n = 10 (0/10)	Paired Student's *t*-test	Pre-test vs Test (mCherry+Saline): t = 0.1931, df = 8, P = 0.8517; Pre-test vs Test (ChR2+Saline): t = 2.931, df = 8, P = 0.0190; Pre-test vs Test (ChR2+CNO): t = 1.191, df = 9, P = 0.2640
Figure 8B	$mPFC^{Ext}$–ZI(mCherry),<br>$mPFC^{Ext}$–ZI(ChR2),<br>$mPFC^{Ext}$–SuM(mCherry),<br>$mPFC^{Ext}$–SuM(ChR2)	n = 15 (5/10),<br>n = 16 (7/9),<br>n = 15 (7/8),<br>n = 17 (6/11)	Two-way ANOVA	$mPFC^{Ext}$–ZI (mCherry vs ChR2):<br>virus $F_{1,27}$ = 11.95, P = 0.0018; sex $F_{1,27}$ = 0.09692, P = 0.7579; virus × sex $F_{1,27}$ = 0.3736, P = 0.5461;<br>$mPFC^{Ext}$–SuM (mCherry vs ChR2):<br>virus $F_{1,28}$ = 9.974, P = 0.0038; sex $F_{1,28}$ = 1.758, P = 0.1956; virus × sex $F_{1,28}$ = 1.143, P = 0.2942
Figure 8C	$mPFC^{Ext}$–ZI(mCherry),<br>$mPFC^{Ext}$–ZI(ChR2),<br>$mPFC^{Ext}$–SuM(mCherry),<br>$mPFC^{Ext}$–SuM(ChR2)	n = 15 (5/10),<br>n = 16 (7/9),<br>n = 15 (7/8),<br>n = 17 (6/11)	Three-way ANOVA	virus $F_{1,55}$ = 21.82, P<0.0001;<br>region $F_{1,55}$ = 1.149, P = 0.2885;<br>sex $F_{1,55}$ = 0.5265, P = 0.4712;<br>virus × region $F_{1,55}$ = 0.03062, P = 0.8617;<br>virus × sex $F_{1,55}$ = 0.1106, P = 0.7407;<br>sex × region $F_{1,55}$ = 1.350, P = 0.2503;<br>sex × virus × region $F_{1,55}$ = 1.415, P = 0.2393
Figure 8D	$mPFC^{Ext}$–ZI(mCherry),<br>$mPFC^{Ext}$–ZI(ChR2),<br>$mPFC^{Ext}$–SuM(mCherry),<br>$mPFC^{Ext}$–SuM(ChR2)	n = 7 (3/4),<br>n = 8 (4/4),<br>n = 7 (4/3),<br>n = 7 (4/3)	Two-way ANOVA	$mPFC^{Ext}$–ZI (mCherry vs ChR2):<br>virus $F_{1,11}$ = 7.87, P = 0.0171; sex $F_{1,11}$ = 0.2182, P = 0.6496; virus × sex $F_{1,11}$ = 0.1708, P =

				0.6873; mPFC^{Ext}–SuM (mCherry vs ChR2): virus $F_{1,10}$ = 37.02, P = 0.0001; sex $F_{1,10}$ = 0.0112, P = 0.9178; virus × sex $F_{1,10}$ = 0.6761, P = 0.4301
Figure 8E	mPFC^{Ext}–ZI(mCherry), mPFC^{Ext}–ZI(ChR2), mPFC^{Ext}–SuM(mCherry), mPFC^{Ext}–SuM(ChR2)	n = 7 (3/4), n = 8 (4/4), n = 7 (4/3), n = 7 (4/3)	Three-way ANOVA	virus $F_{1,21}$ = 40.70, P<0.0001; region $F_{1,21}$ = 2.352, P = 0.1400; sex $F_{1,21}$ = 0.05776, P = 0.8124; virus × region $F_{1,21}$ = 6.550, P = 0.0183; virus × sex $F_{1,21}$ = 0.1040, P = 0.7502; sex × region $F_{1,21}$ = 0.1567, P = 0.6962; sex × virus × region $F_{1,21}$ = 0.7840, P = 0.3859
Figure 8G	mPFC^{Ext}–ZI(mCherry), mPFC^{Ext}–ZI(hM3Dq), mPFC^{Ext}–SuM(mCherry), mPFC^{Ext}–SuM(hM3Dq)	n = 8 (4/4), n = 9 (5/4), n = 9 (5/4), n = 9 (4/5)	Two-way ANOVA	mPFC^{Ext}–ZI (mCherry vs hM3Dq): virus $F_{1,13}$ = 0.05008, P = 0.8264; sex $F_{1,13}$ = 0.07332, P = 0.7908; virus × sex $F_{1,13}$ = 0.1128, P = 0.7423; mPFC^{Ext}–SuM (mCherry vs hM3Dq): virus $F_{1,14}$ = 9.555, P = 0.0080; sex $F_{1,14}$ = 0.05114, P = 0.8244; virus × sex $F_{1,14}$ = 0.1820, P = 0.6761
Figure 8I	mPFC^{Ext}–SuM(mCherry), mPFC^{Ext}–SuM(hM3Dq)	n = 20 (section), n = 20 (section)	Unpaired Student's *t* test	t = 12.43, df = 38, P < 0.0001
Figure 9B	Control, SPS	n = 9 (0/9), n = 9 (0/9)	Two-way repeated measures ANOVA	extinction training (Day 2–Day 4) $F_{1,16}$ = 65.41, P < 0.0001; extinction retrieval $F_{1,16}$ = 0.02955, P = 0.8657.

Figure 9C	Control, SPS	n = 9 (0/9),<br>n = 9 (0/9)	Unpaired Student's *t* test	t = 3.439, df = 16, P = 0.0034
Figure 9F	sgControl, sgSirt1	n = 24 (section),<br>n = 24 (section)	Unpaired Student's *t* test	t = 10.01, df = 46, P < 0.0001
Figure 9G	sgControl, sgSirt1	n = 8 (4/4),<br>n = 9 (4/5)	Two-way ANOVA	virus $F_{1,13}$ = 7.172, P = 0.0190; sex $F_{1,13}$ = 0.1393, P = 0.7150; virus × sex $F_{1,13}$ = 0.7158, P = 0.4128;
Figure 9I	Control, SIRT1-OE	n = 24 (section),<br>n = 24 (section)	Unpaired Student's *t* test	t = 18.37, df = 46, P < 0.0001
Figure 9J	Control, SIRT1-OE	n = 8 (4/4),<br>n = 8 (4/5)	Two-way ANOVA	virus $F_{1,12}$ = 31.72, P = 0.0001; sex $F_{1,12}$ = 1.190, P = 0.2968; virus × sex $F_{1,12}$ = 2.063, P = 0.1765;
Supplemental Figure 1B	Ext Retr, Fear Renewal	n = 7 (3/4),<br>n = 7 (4/3)	Mixed-degin ANOVA	memory test $F_{1,10}$ = 12.344, P = 0.006; sex $F_{1,10}$ = 0.007, P = 0.937; memory test × sex $F_{1,10}$ = 0.007, P = 0.936
Supplemental Figure 1E	Ext Retr+Saline, Ext Retr+4-OHT, Homecage+4-OHT	n = 4 (0/4),<br>n = 4 (0/4),<br>n = 4 (0/4)	One-way ANOVA with Tukey's multiple-comparison test	Ext Retr+Saline vs Ext Retr+4-OHT: P < 0.0001; Ext Retr+4-OHT vs Homecage+4-OHT: P < 0.0001
Supplemental Figure 1F	PL, IL	n = 3 (0/3),<br>n = 3 (0/3)	Unpaired Student's *t* test	t = 0.1769, df = 4, P = 0.8681
Supplemental Figure 1I	Ext Retr, Fear Renewal	n = 4 (0/4),<br>n = 4 (0/4)	Unpaired Student's *t* test	GFP^+(%$DAPI^+$): t = 1.491, df = 6, P = 0.1867; c-Fos^+(%$DAPI^+$): t = 0.08844, df = 6, P = 0.9324; Reactivation/chance: t = 7.214, df = 6, P = 0.0004
Supplemental Figure 2B	Control, ChR2	n = 8 (0/8),<br>n = 8 (0/8)	Unpaired Student's *t* test	t = 0.6148, df = 14, P = 0.5485
Supplemental Figure 2C	Control, ChR2	n = 8 (0/8),<br>n = 8 (0/8)	Two-way repeated measures ANOVA	virus $F_{1,35}$ = 0.002424, P = 0.9610
Supplemental Figure 2E	Control, ChR2	n = 8 (0/8),<br>n = 8 (0/8)		virus $F_{1,35}$ = 0.9504, P = 0.3363
Supplemental Figure 3C	Ext Retr, Fear Renewal	n = 5 (0/5) per group	Paired Student's *t*-test	MPO: t = 0.4397, df = 4, P = 0.6829; AM: t = 2.348, df = 4, P = 0.0787; MD: t = 2.874, df = 4, P = 0.0453; LHA: t = 3.671,

				df = 4, P = 0.0214; ZI: t = 4.902, df = 4, P = 0.0080; SuM: t = 1.921, df = 4, P = 0.1272; PAG: t = 4.215, df = 4, P = 0.0135
Supplemental Figure 3E	Baseline, During	n = 5 (0/5) per group	Paired Student's *t*-test	MPO: t = 1.169, df = 4, P = 0.3074; AM: t = 0.7306, df = 4, P = 0.5055; MD: t = 1.048, df = 4, P = 0.3538; LHA: t = 2.832, df = 4, P = 0.0472; ZI: t = 1.768, df = 4, P = 0.1518; SuM: t = 3.369, df = 4, P = 0.0281; PAG: t = 1.261, df = 4, P = 0.2759
Supplemental Figure 4D	Ext Retr, Fear Renewal	n = 5 (0/5) per group	Paired Student's *t*-test	BLA: t = 4.235, df = 4, P = 0.0133
Supplemental Figure 4F	Baseline, During	n = 5 (0/5) per group	Paired Student's *t*-test	BLA: t = 1.008, df = 4, P = 0.3707
Supplemental Figure 5C	$mPFC^{Ext}$–ZI(Ext Retr), $mPFC^{Ext}$–ZI(Water intake), $mPFC^{Ext}$–SuM(Ext Retr), $mPFC^{Ext}$–SuM(Water intake)	n = 4 (0/4), n = 4 (0/4), n = 4 (0/4), n = 4 (0/4)	One-way ANOVA with Tukey's multiple-comparison test	$mPFC^{Ext}$–ZI(Ext Retr) vs $mPFC^{Ext}$–ZI(Water intake): P = 0.0065; $mPFC^{Ext}$–ZI(Ext Retr) vs $mPFC^{Ext}$–SuM(Ext Retr): P = 0.0752; $mPFC^{Ext}$–ZI(Water intake) vs $mPFC^{Ext}$–SuM(Water intake): P = 0.0016; $mPFC^{Ext}$–SuM(Ext Retr) vs $mPFC^{Ext}$–SuM(Water intake): P = 0.0172
Supplemental Figure 6B	Normal-intake, Low-intake	n = 18 (0/18), n = 7 (0/7)	Unpaired Student's *t* test	Water intake (mL): t = 7.480, df = 23, P < 0.0001; Number of water intakes: t = 7.228, df = 23, P < 0.0001
Supplemental Figure 6C	Normal-intake, Low-intake	n = 18 (0/18), n = 7 (0/7)	Unpaired Student's *t* test	24 h water intake (mL): t = 0.4199, df = 23, P = 0.6785; Body weight (g): t = 0.4480, df = 23, P < 0.6583
Supplemental Figure 11B	$mPFC^{Ext}$–ZI(mCherry+Saline,	n = 9 (0/9), n = 9 (0/9),	One-way ANOVA with Bonferroni's	$mPFC^{Ext}$–ZI(mCherry+Saline) vs

	ChR2+Saline, ChR2+CNO); mPFC^{Ext}–SuM(mCherry+Saline, ChR2+Saline, ChR2+CNO)	n = 8 (0/8); n = 9 (0/9), n = 9 (0/9), n = 10 (0/10)	multiple-comparison test	mPFC^{Ext}–ZI(ChR2+Saline): P = 0.0037; mPFC^{Ext}–ZI(ChR2+Saline) vs mPFC^{Ext}–ZI(ChR2+CNO): P = 0.0482; mPFC^{Ext}–SuM(mCherry+Saline) vs mPFC^{Ext}–SuM(ChR2+Saline): P < 0.0001; mPFC^{Ext}–SuM(ChR2+Saline) vs mPFC^{Ext}–SuM(ChR2+CNO): P = 0.0037
Supplemental Figure 12C	Ext Retr, SR test	n = 4 (0/4), n = 4 (0/4)	Unpaired Student's *t* test	t = 0.5855, df = 6, P = 0.5795
Supplemental Figure 12F	mCherry, ChR2	n = 8 (0/8), n = 7 (0/7)	Two-way repeated measures ANOVA	$F_{1,13}$ = 0.5196, P = 0.4838
Supplemental Figure 12H	mCherry, ChR2	n = 8 (0/8), n = 8 (0/8)	Two-way repeated measures ANOVA	$F_{1,14}$ = 11.82, P = 0.0040
Supplemental Figure 13D	mCherry, ChR2	n = 8 (0/8), n = 8 (0/8)	Two-way repeated measures ANOVA	Extinction retrieval $F_{1,14}$ = 0.03128, P = 0.8621; Spontaneous recovery $F_{1,14}$ = 13.17, P = 0.0027
Supplemental Figure 13E	mCherry, ChR2	n = 8 (0/8), n = 8 (0/8)	Paired Student's *t*-test; Two-way repeated measures ANOVA	Pre-test vs Test (mCherry): t = 0.2873, df = 7, P = 0.7822; Pre-test vs Test (ChR2): t = 2.598, df = 7, P = 0.0355
Supplemental Figure 15B	Normal-intake, Low-intake	n = 42 (19/23), n = 13 (5/8)	Two-way ANOVA	virus $F_{1,51}$ = 71.45, P < 0.0001; sex $F_{1,51}$ = 0.2943, P = 0.5899; virus × sex $F_{1,51}$ = 0.04931, P = 0.8252
Supplemental Figure 16B	mPFC^{Ext}–ZI: Normal-intake(mCherry), Normal-intake(ChR2), Low-intake(mCherry), Low-intake(ChR2); mPFC^{Ext}–SuM: Normal-intake(mCherry),	n = 15 (5/10), n = 16 (7/9), n = 7 (3/4), n = 8 (4/4); n = 15 (7/8), n = 17 (6/11), n = 7 (4/3), n = 7 (4/3);	Three-way ANOVA	mPFC^{Ext}–ZI: virus $F_{1,38}$ = 15.99, P = 0.0003; water intake $F_{1,38}$ = 24.82, P < 0.0001; sex $F_{1,38}$ = 0.01327, P = 0.9089; virus ×water intake $F_{1,38}$ = 0.08614, P = 0.7707; virus × sex $F_{1,38}$ =

	Normal-intake(ChR2),<br>Low-intake(mCherry),<br>Low-intake(ChR2);			0.01134, P = 0.9157;<br>sex ×water intake $F_{1,38}$ = 0.2517, P = 0.6188;<br>sex × virus ×water intake $F_{1,38}$ = 0.4256, P = 0.5181<br><br>mPFC^{Ext}–SuM:<br>virus $F_{1,38}$ = 35.20, P < 0.0001;<br>water intake $F_{1,38}$ = 17.37, P = 0.0002;<br>sex $F_{1,38}$ = 0.5086, P = 0.4801;<br>virus ×water intake $F_{1,38}$ = 4.830, P = 0.0341;<br>virus × sex $F_{1,38}$ = 0.006849, P = 0.9345;<br>sex ×water intake $F_{1,38}$ = 0.7303, P = 0.3981;<br>sex × virus ×water intake $F_{1,38}$ = 1.396, P = 0.2448
Supplemental Figure 16E	ACSF, CNO	n = 12 (neuron),<br>n = 12 (neuron)	Two-way repeated measures ANOVA	drug $F_{1,66}$ = 608.8, P < 0.0001
Supplemental Figure 16G	ACSF, CNO	n = 12 (neuron),<br>n = 12 (neuron)	Two-way repeated measures ANOVA	drug $F_{1,66}$ = 497.1, P < 0.0001
Supplemental Figure 17B	sgControl, sgSirt1	n = 24 (section),<br>n = 24 (section)	Unpaired Student's *t* test	t = 12.99, df = 46, P < 0.0001
Supplemental Figure 17C	sgControl, sgSirt1	n = 9 (0/9),<br>n = 9 (0/9)	Unpaired Student's *t* test	t = 3.030, df = 16, P < 0.0080
Supplemental Figure 18B	Control, SIRT1-OE	n = 7 (0/7),<br>n = 7 (0/7)	Two-way repeated measures ANOVA	fear conditioning $F_{1,12}$ = 1.307, P = 0.2752;<br>extinction training $F_{1,12}$ = 0.8565, P = 0.3729;<br>extinction retrieval $F_{1,12}$ = 0.007229, P = 0.9336
Supplemental Figure 18C	Control, SIRT1-OE	n = 20 (section),<br>n = 20 (section)	Unpaired Student's *t* test	t = 16.72, df = 38, P < 0.0001
